# 5-Nitrofuran-Tagged Oxazolyl Pyrazolopiperidines: Synthesis and Activity against ESKAPE Pathogens

**DOI:** 10.3390/molecules28186491

**Published:** 2023-09-07

**Authors:** Elizaveta Rogacheva, Lyudmila Kraeva, Alexey Lukin, Lyubov Vinogradova, Kristina Komarova, Mikhail Chudinov, Maxim Gureev, Evgeny Chupakhin

**Affiliations:** 1 Pasteur Institute of Epidemiology and Microbiology, Saint Petersburg 197101, Russia; elizvla@yandex.ru (E.R.);; 2 Lomonosov Institute of Fine Chemical Technologies, MIREA—Russian Technological University, Moscow 119454, Russia; 3 Laboratory of Bio- and Chemoinformatics, I. M. Sechenov First Moscow State Medical University, Moscow 119991, Russia; 4 Molecular Modeling Laboratory, HSE University, Saint-Petersburg 190121, Russia; 5 Institute of Living Systems, Immanuel Kant Baltic Federal University, Kaliningrad 236041, Russia

**Keywords:** tetrahydropyrazolopyridine, 5-nitrofuran derivatives, ESKAPE pathogens, antibacterial testing, nitroreductase, flexible docking, strained ligand–protein interactions

## Abstract

A series of eight 5-nitrofuran-tagged oxazolyl tetrahydropyrazolopyridines (THPPs) has been prepared in six stages with excellent regioselectivity. The testing of these compounds against pathogens of the ESKAPE panel showed a good activity of lead compound 1-(2-methoxyethyl)-5-(5-nitro-2-furoyl)-3-(1,3-oxazol-5-yl)-4,5,6,7-tetrahydro-1*H*-pyrazolo[4,3-c] pyridine (**13g**), which is superior to nitrofurantoin. These results confirmed the benefit of combining a THPP scaffold with a nitrofuran warhead. Certain structure–activity relationships were established in the course of this study which were rationalized by the induced-fit docking experiments in silico.

## 1. Introduction

**Figure 1:**
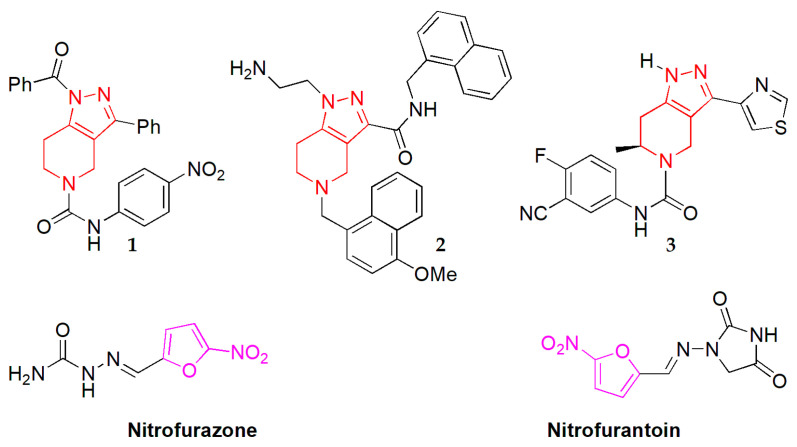
THPP-based active compounds (THPP scaffold marked as red) and nitrofuran-based medicines (5-nitrofuranoyl moiety marked as magenta).

There are a number of reasons, both medicinal and economical, for developing the new types of antibacterial agents [1]. Most of the antibacterial drugs used belong to several groups. The chemical structures and the drug action mechanism of each group are similar and are aimed at a certain biological target—an enzyme specific for some kind of pathogen. The high genetic variability of bacteria allows them to quickly adapt to the drug and often resistance spreads to the entire group. The use of more toxic compounds is limited by their low selectivity. Moreover, the upsurge of resistance to certain chemical structure strains sometimes occurs faster than the creation of drugs based on this structure. The development of new, structure-based antibiotics is rendered unprofitable by this process. Discovering new types of selective antibacterial agents that target previously unexplored biological pathways is a crucial and pressing objective. Doing so would enable us to overcome the challenge of resistance, which has greatly altered the effectiveness of existing antibiotics. Therefore, it is imperative that we focus on developing innovative antibacterial drugs that are capable of effectively combating drug-resistant bacteria. Modern medicinal chemistry trends are developing polypharmacological drugs with complex, multi-targeting effects [2,3,4,5,6]. Based on recent studies and previously acquired data from sources [7,8,9], we can now suggest pharmacophore structures derived from a combination of molecular fragments with varying antibiotic properties. Compounds derived from tetrahydropyrazolopyridine (THPP) have demonstrated a very wide spectrum of biological activity such as different enzyme inhibition [10,11,12,13], antiviral [14,15], and antimicrobial [16,17,18] activity. This molecule presents three potential sites for modification, offering a tremendous opportunity for innovative design. 1-Benzoyl-*N*-(4-nitrophenyl)-3-phenyl-6,7-dihydro-1*H*-pyrazolo[4,3-c]pyridine-5(4*H*)-carboxamide (**1**) (Figure 1) inhibits the pantothenate synthetase of *Mycobacterium tuberculosis* (MTB) and growth of MTB with an MIC of 26.7 mM [18]. 1-(2-Aminoethyl)-5-((4-methoxynaphthalen-1-yl)methyl)-*N*-(naphthalen-1-ylmethyl)-4,5,6,7-tetrahydro-1*H*-pyrazolo[4,3-c]pyridine-3-carboxamide (**2**) exhibits antitriposomal activity in the nanomolar range due to the inhibition of protein–protein interactions in parasite cells [19]. (6*S*)-*N*-(3-cyano-4-fluorophenyl)-6-methyl-3-(1,3-thiazol-4-yl)-1,4,6,7-tetrahydro-5*H*-pyrazolo[4,3-*c*]pyridine-5-carboxamide (**3**) blocks hepatitis B viral capsid assembly [15].

**Scheme 1:**
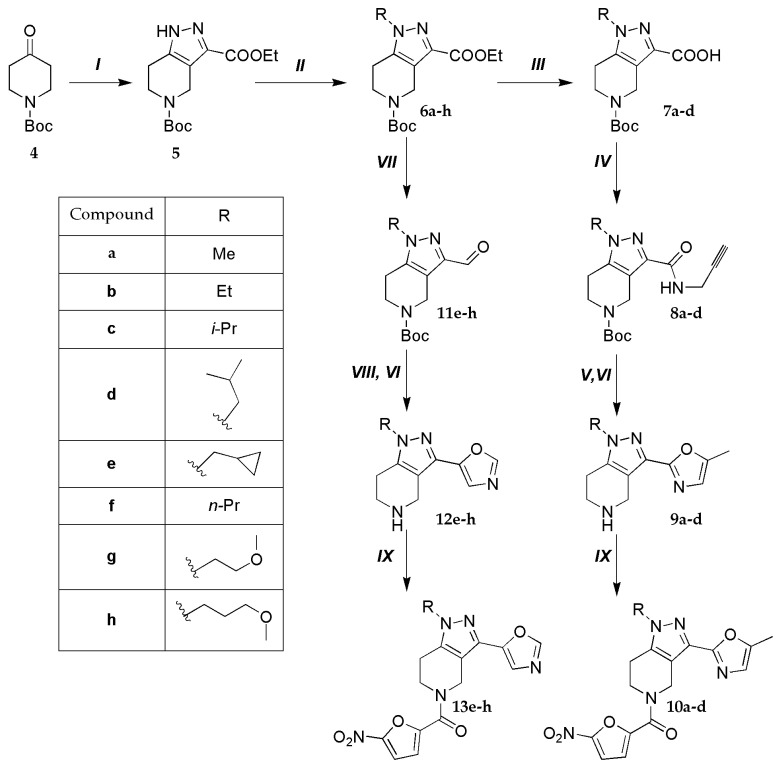
Synthesis of target compounds. Reagents and conditions: (I) diethyl oxalate, THF, LDA, −78 °C → rt, then AcOH, hydrazine, 52% over 2 steps; (II) NaH, toluene, alkyl halide, rt, 58–92%; (III) KOH, MeOH/water, rt, 86–95%; (IV) propargyl amine, CDI, DCM, rt, 79–82%; (V) Cs_2_CO_3_, DMSO, 100 °C, 58–70%; (VI) HCl, dioxane, rt; (VII) LiAlH_4_, THF, 0 °C, then MnO_2_, DCM, rt, 36–61% over 2 steps; (VIII) TosMic, K_2_CO_3_, MeOH, reflux, 38–52%; (IX) 5-nitro-2-furoic acid, CDI, DMF, TEA, rt, 39–62%.

The 5-nitrofuran-2-yl moiety is an old reliable warhead. Its mechanism of action involves the reduction of the nitro group by nitroreductase in the bacterial cell wall to form free radicals that can react with bacterial targets [20]. Unfortunately, this mechanism suggests the significant toxicity of 5-nitrofuran derivatives to human cells. Some famous antimicrobials such as nitrofurazone or nitrofurantoin (Figure 1) are built around this warhead. Finally, oxazole is a privileged structure found in many drugs. The introduction of an oxazole fragment during optimization is a common technique [21]. Can the combination of these three structural elements give a new impulse to antimicrobial therapy? To answer this question, we synthesized several compounds according to Scheme 1.

## 2. Results and Discussion

### 2.1. Chemistry

Tetrahydropyrazolopyridine (**5**) was prepared according to the published procedure [22]. The pyrazole ring of **5** was N-alkylated by various alkyl halides with excellent regioselectivity (more than 99% of N1-isomer) using sodium hydride in toluene to give pyrazole-fused piperidines **6a**–**h**. Previously described alkylation methods [17] do not show such high regioselectivity. Compounds **6a**–**d** were converted to the corresponding carboxylic acids **7a**–**d** with KOH in an aqueous methanol solution and then to the propargyl amides **8a**–**d**. Cyclization of **8** in the next step was carried out in the presence of cesium carbonate in DMSO at 100 °C with 60–70% yields. Esters **6e**–**h** were converted to the aldehydes **11e**–**h** by LiAlH_4_ reduction and subsequent oxidation with MnO_2_. Aldehydes **11e**–**h** were treated with TosMic in the potassium carbonate presence. All 3-oxazolyl-THPP’s were then BOC deprotected and the resulting amine salts **9a**–**d** and **12e**–**h** were acylated by 5-nitro-2-furoic acid to give target compounds **10a**–**d** and **13e**–**h**.

### 2.2. Activity against ESKAPE Pathogens

Nitrofurans **10a**–**d** and **13e**–**h** were tested against Gram-positive (*S. aureus* and *E. faecium*) or Gram-negative (*P. aeruginosa*, *A. baumannii*, *K. pneumoniae*, *E. cloacae*) pathogens of the so-called ESKAPE panel [23]. These pathogens embody the top five bacterial families with high capacity to obtain multi-drug resistance and which are one of the most important global health threats urgently in need of new antibiotic research. There were two clinically used antibiotics—nitrofurantoin and ciprofloxacin—that were used as positive controls and comparators. The compounds were initially screened at a single concentration to determine the presence and the diameter of the bacterial growth inhibition zone around the drug-treated disk. Those compounds that displayed growth inhibition were tested in serial dilution mode to determine the minimum inhibitory concentration (MIC) (Table 1).

Important observations can be made from the data presented in Table 1: compound **10** does not show any activity, unlike **13**, and the MIC of the lead compound **13g** is superior to the comparators for most cases. Molecular modeling was carried out to elucidate the decrease in activity compared to nitrofurantoin.

### 2.3. Molecular Modeling

Calculations were carried out for the three most active compounds: LK01509 (**13g**), LK01513 (**13f**), and LK01514 (**13h**) (Table 2). Two proteins were chosen as potential targets for the discovered nitrofuran-based ligands: NfsB from *E. coli* and NfsA from oxygen-insensitive NADPH nitroreductase (PDB ids: 1YKI, 7NB9). The binding poses of the ligands were predicted using the induced-fit docking method (IFD). This technique, unlike molecular docking, assumes protein structural flexibility. Gibbs free energy (ΔG) was estimated for each ligand-binding pose in the presence of an implicit solvent. Calculations were carried out using the MM-GBSA method. The strain energy value distribution, which reflects the majority of strained protein–ligand interactions, is of particular importance. This characteristic can explain the lack of proper protein interaction and, as a result, lower activity.

Based on the IFD data, it is clear that NfsB (1YKI) is the preferred target, as all three compounds have the highest predicted binding affinity, both in terms of the scoring function and in terms of Gibbs free energy and strain energy in the ligand–protein complex. Activity level distinction is further complicated by the fact that substances with a low level of activity assessed empirically have more favorable energy parameters for binding to NfsB. The strain energy value is an exception, indicating that the ligand–protein complex has the potential for conformational lability. It is important to understand that lower strain energy values result in a more stable ligand–protein combination with less conformational lability.

However, the investigation of the actual packing of the examined compounds in the active cavity of the proteins reveals all: there is no reproduction of the requisite pharmacophore properties in the case of compounds LK01513/14. Even with high-scoring function rates, this produces a false-positive outcome.

**Figure 2:**
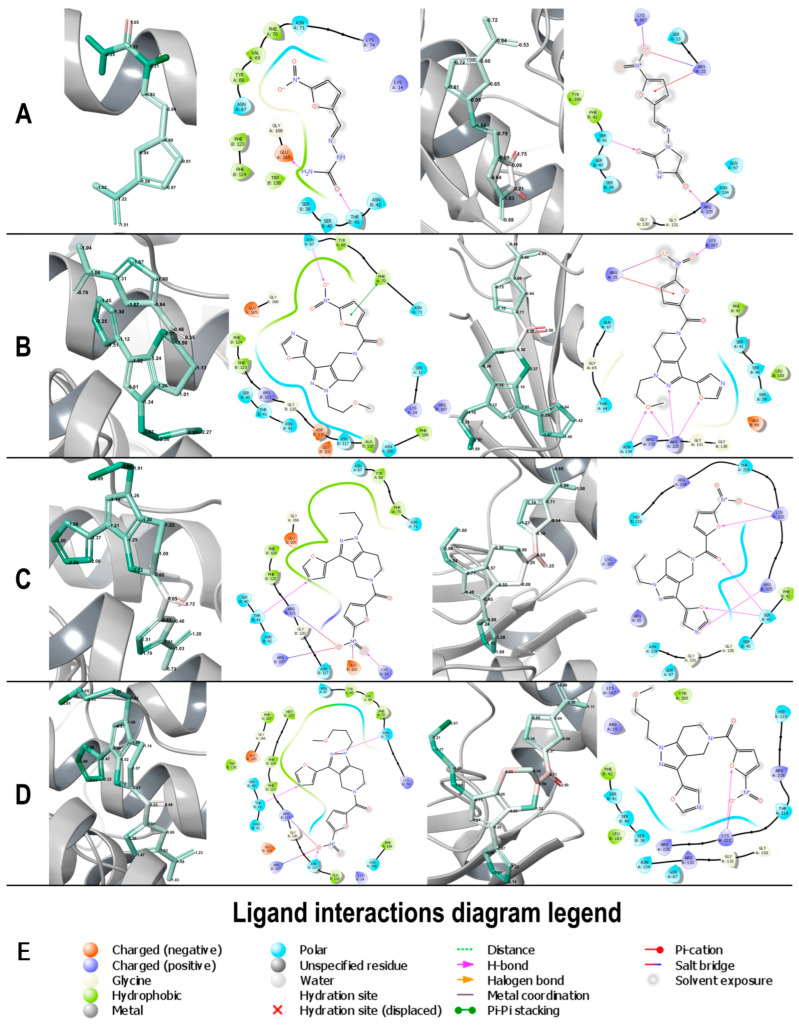
Three-dimensional model of interaction with per-atom ΔG assignment and ligand interaction diagram: (**A**) nitrofurazone—NfsB protein (left) and nitrofurantoin—NfsA (right); (**B**) LK01509—NfsB protein (left) and NfsA (right); (**C**) LK01513—NfsB protein (left) and NfsA (right); (**D**) LK01514—NfsB protein (left) and NfsA (right); (**E**) ligand interaction diagram legend.

In turn, the LK01509 compound in association with NfsB replicates all required interactions in the same manner, as the control compound (nitrofurazone) does. Less active LK01513/14, on the other hand, binds in an inverted manner: it is not orientated to the active site’s matching lysins (Lys14/74) but inside the protein, producing a salt bridge with Arg107/121 (Figure 2).

The chemical LK01509 binds at the same level as the reference compound with nitroreductase NfsA (7NB9). This demonstrates the possible target specificity of the investigated chemical (albeit it is less active than NfsB). The structures of LK01513/14 produce poorer results, with stronger strained contacts and a higher free energy (ΔG). Binding pose research also revealed that the interaction profile of LK01513/14 compounds was inaccurate (see ligand interaction diagrams in Figure 2).

On the basis of the performed calculations, we can conclude that the compound LK01509 can be used—NfsB protein as the primary target and NfsA protein as the alternative target—due to the similarity in binding profiles with the control compounds. Activity loss is linked to the imbalanced interaction potential of the N-linked aliphatic substituent of the pyrazolopyridine scaffold. In the case of LK01509, LK01513, and LK01514, the sidechain per-atom binding potential is −5.83, −6.84, and −9.78 kcal/mol, given by lipophilic interactions (see Figure 2, left).The same situation is found with NfsA. The growing role of lipophilic interactions with following amino acids leads to a binding pose rearrangement with target activity loss following.

## 3. Materials and Methods

### 3.1. Chemistry

All reactions were conducted in oven-dried glassware in an atmosphere of nitrogen. Melting points were measured with a Buchi B-520 melting point apparatus and were not corrected. The NMR spectra were recorded on a Bruker MSL-300 spectrometer at 25 °C (^1^H: 300 MHz; ^13^C: 75 MHz; chemical shifts are reported as parts per million (δ, ppm)) in dimethyl sulfoxide (DMSO)-*d*_6_, CDCl_3_ or D_2_O; the residual solvent peaks were used as internal standards: 7.28 ppm for ^1^H in CDCl_3_, 2.50 ppm for ^1^H in DMSO-*d*_6_ and 4.79 ppm for ^1^H in D_2_O, 40.01 and 77.02 ppm for ^13^C in DMSO-*d*_6_ and CDCl_3_. Copies of the NMR spectra of synthesized compounds are presented in Supplementary Materials. Mass spectra were recorded using the Shimadzu LCMS-2020 system with ESI. High-resolution mass spectra (HRMS) were recorded using a Bruker microTOF spectrometer (ionization by electrospray, positive ions detection). Analytical thin-layer chromatography was carried out on Sorbfil UV-254 silica gel plates (Imid Ltd., Krasnodar, Russia) using appropriate mixtures of solvent. The compounds were visualized with short-wavelength UV light. Column chromatography was performed on silica gel 60 (230–400 mesh). All reagents and solvents were obtained from commercial sources and used without purification. 5-tert-butyl 3-ethyl 1,4,6,7-tetrahydro-5*H*-pyrazolo[4,3-c]pyridine-3,5-dicarboxylate (**5**) was synthesized according to the procedure reported by Arrington et al. [22] with 52% yield over two steps.

#### 3.1.1. General Procedure for the Synthesis of Compounds **6a**–**h**

To the suspension of the 60% sodium hydride (62.5 mmol, 2.5 g) in anhydrous toluene (150 mL), 5-tert-butyl 3-ethyl 1,4,6,7-tetrahydro-5*H*-pyrazolo[4,3-c] pyridine-3,5-dicarboxylate (**5**) (33.9 mmol, 10 g) was added followed by alkyl halide (42.4 mmol). The reaction progress was monitored by TLC (chloroform/methanol 97:3). After stirring at room temperature for 6 h, the reaction mass was washed with water (2 × 50 mL), then 5% aqueous potassium carbonate solution (50 mL) and 5% aqueous citric acid solution (50 mL), dried over anhydrous Na_2_SO_4_, filtered, and concentrated by rotary evaporation under vacuum.

*5-tert-butyl 3-ethyl 1-methyl-1,4,6,7-tetrahydro-5H-pyrazolo[4,3-c]pyridine-3,5-dicarboxylate* (**6a**). Yield 10 g (95%), white solid, m.p. 124–124.5 °C. ^1^H NMR (300 MHz, CDCl_3_) δ 4.58 (s, 2H), 4.37 (q, *J* = 7.1 Hz, 2H), 3.80 (s, 3H), 3.70 (t, *J* = 5.7 Hz, 2H), 2.66 (t, *J* = 5.6 Hz, 2H), 1.46 (s, 9H), 1.37 (t, *J* = 7.1 Hz, 3H); ^13^C NMR (75 MHz, CDCl_3_) δ 162.5, 155.1, 138.7, 138.1, 117.8, 80.3, 60.8, 41.5, 40.1, 36.5, 28.5, 22.0, 14.5. LCMS (ESI): *m*/*z* (M + H^+^) calcd, 310.2; found, 310.4.

*5-tert-butyl 3-ethyl 1-ethyl-1,4,6,7-tetrahydro-5H-pyrazolo[4,3-c]pyridine-3,5-dicarboxylate* (**6b**). Yield 9.4 g (86%), white solid, m.p. 168–169 °C. ^1^H NMR (300 MHz, DMSO-*d*_6_) δ 4.47 (s, 2H), 4.25 (q, *J* = 7.0 Hz, 2H), 4.09 (q, *J* = 7.2 Hz, 2H), 3.61 (t, *J* = 5.5 Hz, 2H), 2.71 (t, *J* = 5.3 Hz, 2H), 1.41 (s, 9H), 1.35–1.24 (m, 6H); ^13^C NMR (75 MHz, DMSO-*d*_6_) δ 161.98, 154.01, 138.01, 136.20, 116.67, 79.21, 59.99, 44.14, 41.13, 40.34, 28.01, 21.00, 14.90, 13.80. LCMS (ESI): *m*/*z* (M + H^+^) calcd, 324.2; found, 324.4.

*5-tert-butyl 3-ethyl 1-(propan-2-yl)-1,4,6,7-tetrahydro-5H-pyrazolo[4,3-c]pyridine-3,5-dicarboxylate* (**6c**). Yield 8.6 g (75%), white solid, m.p. 119–120 °C. ^1^H NMR (300 MHz, DMSO-*d*_6_) δ 4.56–4.45 (m, 3H), 4.26 (q, *J* = 7.1 Hz, 2H), 3.62 (t, *J* = 5.7 Hz, 2H), 2.73 (t, *J* = 5.5 Hz, 2H), 1.42 (s, 9H), 1.38 (d, *J* = 6.6 Hz, 6H), 1.29 (t, *J* = 7.1 Hz, 3H); ^13^C NMR (75 MHz, DMSO-*d*_6_) δ 161.86, 153.97, 137.39, 136.63, 116.41, 79.09, 59.84, 50.40, 40.96, 27.95, 22.03, 21.17, 14.16. LCMS (ESI): *m*/*z* (M + H^+^) calcd, 338.2; found, 338.4.

*5-tert-butyl 3-ethyl 1-(2-methylpropyl)-1,4,6,7-tetrahydro-5H-pyrazolo[4,3-c]pyridine-3,5-dicarboxylate* (**6d**). Yield 9.3 g (78%), clear oil. ^1^H NMR (300 MHz, DMSO-*d*_6_) δ 4.48 (s, 2H), 4.25 (q, *J* = 7.1 Hz, 2H), 3.87 (d, *J* = 7.4 Hz, 2H), 3.60 (t, *J* = 5.7 Hz, 2H), 2.69 (t, *J* = 5.6 Hz, 2H), 2.10 (hept, *J* = 6.8 Hz, 1H), 1.41 (s, 9H), 1.29 (t, *J* = 7.1 Hz, 3H), 0.84 (d, *J* = 6.7 Hz, 6H); ^13^C NMR (75 MHz, DMSO-*d*_6_) δ 161.82, 154.04, 138.59, 136.87, 116.28, 79.13, 59.90, 55.94, 40.72, 28.84, 27.95, 21.42, 19.50, 14.12. LCMS (ESI): *m*/*z* (M + H^+^) calcd, 352.2; found, 352.4.

*5-tert-butyl 3-ethyl 1-(cyclopropylmethyl)-1,4,6,7-tetrahydro-5H-pyrazolo[4,3-c]pyridine-3,5-dicarboxylate* (**6e**). Yield 7.9 g (67%), clear oil. ^1^H NMR (300 MHz, DMSO-*d*_6_) δ 4.49 (s, 2H), 4.26 (q, *J* = 7.1 Hz, 2H), 3.96 (d, *J* = 7.0 Hz, 2H), 3.62 (t, *J* = 5.6 Hz, 2H), 2.73 (t, *J* = 5.5 Hz, 2H), 1.42 (s, 9H), 1.29 (t, *J* = 7.1 Hz, 3H), 1.25–1.15 (m, 1H), 0.55–0.46 (m, 2H), 0.38–0.30 (m, 2H). ^13^C NMR (75 MHz, DMSO-*d*_6_) δ 161.92, 154.07, 138.05, 136.76, 116.69, 79.21, 59.99, 53.56, 41.20, 40.62, 28.00, 21.36, 14.21, 11.11, 3.65. LCMS (ESI): *m*/*z* (M + H^+^) calcd, 350.2; found, 350.4.

*5-tert-butyl 3-ethyl 1-propyl-1,4,6,7-tetrahydro-5H-pyrazolo[4,3-c]pyridine-3,5-dicarboxylate* (**6f**). Yield 10.4 g (91%), white solid, m.p. 64–65 °C. ^1^H NMR (300 MHz, DMSO-*d*_6_) δ 4.59 (s, 2H), 4.37 (q, *J* = 7.1 Hz, 2H), 4.01 (t, *J* = 7.3 Hz, 2H), 3.70 (t, *J* = 5.4 Hz, 2H), 2.66 (t, *J* = 5.1 Hz, 2H), 1.84 (h, *J* = 7.3 Hz, 2H), 1.47 (s, 9H), 1.37 (t, *J* = 7.1 Hz, 3H), 0.89 (t, *J* = 7.4 Hz, 3H). ^13^C NMR (75 MHz, DMSO-*d*_6_) δ 162.26, 154.43, 138.76, 137.17, 116.84, 79.56, 60.35, 50.90, 41.54, 40.98, 28.35, 21.51, 14.55. LCMS (ESI): *m*/*z* (M + H^+^) calcd, 338.2; found, 338.4.

*5-tert-butyl 3-ethyl 1-(2-methoxyethyl)-1,4,6,7-tetrahydro-5H-pyrazolo[4,3-c]pyridine-3,5-dicarboxylate* (**6g**). Yield 8.15 g (68%), clear oil. ^1^H NMR (300 MHz, DMSO-*d*_6_) δ 4.48 (s, 2H), 4.32–4.17 (m, 4H), 3.65 (t, *J* = 5.2 Hz, 2H), 3.60 (t, *J* = 5.6 Hz, 2H), 3.20 (s, 3H), 2.71 (t, *J* = 5.4 Hz, 2H), 1.41 (s, 9H), 1.29 (t, *J* = 7.1 Hz, 3H). ^13^C NMR (75 MHz, DMSO-*d*_6_) δ 162.21, 154.42, 139.63, 137.47, 116.78, 79.54, 71.00, 60.37, 58.48, 49.47, 41.50, 39.86, 28.33, 21.68, 14.52. LCMS (ESI): *m*/*z* (M + H^+^) calcd, 354.2; found, 354.6.

*5-tert-butyl 3-ethyl 1-(2-methoxypropyl)-1,4,6,7-tetrahydro-5H-pyrazolo[4,3-c]pyridine-3,5-dicarboxylate* (**6h**). Yield 7.2 g (58%), clear oil. ^1^H NMR (300 MHz, DMSO-*d*_6_) δ 4.49 (s, 2H), 4.26 (q, *J* = 7.1 Hz, 2H), 4.10 (t, *J* = 6.9 Hz, 2H), 3.61 (t, *J* = 5.6 Hz, 2H), 3.25 (t, *J* = 6.0 Hz, 2H), 3.21 (s, 3H), 2.69 (t, *J* = 5.3 Hz, 2H), 2.03–1.90 (m, 2H), 1.42 (s, 9H), 1.29 (t, *J* = 7.1 Hz, 3H). ^13^C NMR (75 MHz, DMSO-*d*_6_) δ 162.23, 154.42, 138.83, 137.45, 116.81, 79.50, 71.00, 60.38, 58.37, 50.49, 41.50, 41.07, 29.69, 28.33, 21.68, 14.53. LCMS (ESI): *m*/*z* (M + H^+^) calcd, 368.2; found, 368.4.

#### 3.1.2. Synthesis of Compounds **9a**–**d**

*1-methyl-3-(5-methyl-1,3-oxazol-2-yl)-4,5,6,7-tetrahydro-1H-pyrazolo [4,3-c]pyridinehydrochloride* (**9a**). A 50 mL round-bottomed flask was charged with a solution of compound **6a** (5.0 g, 16.16 mmol) in methanol (30 mL). A solution of KOH (2.26 g, 40.4 mmol) in water (6.0 mL) was added and the resulting mixture was stirred at room temperature for 12 h. Methanol was removed in vacuo and the residue was dissolved in water (100 mL). The aqueous solution was extracted with ethyl acetate (3 × 50 mL) and the organic extracts were discarded. The pH of the aqueous phase was carefully adjusted to 5.0 with 5% aqueous HCl and the solution was again extracted with ethyl acetate (3 × 50 mL). The combined organic extracts were dried over anhydrous Na_2_SO_4_, filtered, and concentrated in vacuo to give 2.98 g (65%, assuming analytical purity) of product **7a** as a white solid, which was used in the next step without further purification.

To a solution of 2.98 g (10.6 mmol) of **7a** in 25 mL of CH_2_Cl_2_, 1.89 g (11.6 mmol) of N,N-carbonyldiimidazole was added and stirred for 30 min at room temperature. Then, 0.70 g (13 mmol) of propargylamine was added dropwise to the reaction mixture and stirred overnight. The reaction mixture was poured into water, the organic layer was separated and washed sequentially with 5% aqueous citric acid solution (2 × 50 mL) and 10% aqueous K_2_CO_3_ solution (2 × 50 mL). The organic phase was dried over anhydrous Na_2_SO_4_ and the solvent was removed on a vacuum rotary evaporator to give 1.49 g (44%, assuming analytical purity) of amide **8a** as a colorless oil, which was used in the next step without further purification.

To a solution of 1.49 g (4.7 mmol) of amide **8a** in absolute DMSO (10 mL), Cs_2_CO_3_ (3.00 g, 9.2 mmol) was added and stirred for 2 h at 100 °C. The reaction mixture was poured into 100 mL of water and extracted with EtOAc (3 × 50 mL), and the organic phases were combined and washed with 5% aqueous citric acid solution (2 × 50 mL) followed 10% aqueous K_2_CO_3_ solution (2 × 50 mL). The organic phase was dried over anhydrous Na_2_SO_4_ and the solvent was removed on a vacuum rotary evaporator. Column chromatography on silica gel using 0 → 5% MeOH in CHCl_3_ as eluent afforded the target compound. Fractions containing the target compound were combined and evaporated under vacuum. The residue was dissolved in 10 mL of 1,4-dioxane and 5 mL of a 4 N solution of HCl in 1,4-dioxane was added dropwise. The precipitate that formed was filtered off, washed with ether, and dried to give compound **9a**. Yield 0.84 g (56%, 16% over 3 steps), white solid, m.p. 219–220 °C. ^1^H NMR (300 MHz, D_2_O) δ 7.21 (s, 1H), 4.50 (s, 2H), 3.87 (s, 3H), 3.63 (t, *J* = 5.8 Hz, 2H), 3.13 (t, *J* = 5.8 Hz, 2H), 2.43 (s, 3H); ^13^C NMR (75 MHz, DMSO) δ 155.17, 148.68, 136.93, 135.02, 123.92, 108.85, 39.99, 39.70, 36.50, 18.52, 10.73. LCMS (ESI): *m*/*z* (M + H^+^) calcd, 219.2; found, 219.4.

*1-ethyl-3-(5-methyl-1,3-oxazol-2-yl)-4,5,6,7-tetrahydro-1H-pyrazolo[4,3-c]pyridine hydrochloride* (**9b**). Prepared using **6b** as described for **6a**. Yield 0.68 g (38% over 3 steps), white solid, m.p. 215–216 °C. ^1^H NMR (300 MHz, DMSO-*d*_6_) δ 9.98 (s, 2H), 6.97 (d, *J* = 1.0 Hz, 1H), 4.21 (s, 2H), 4.12 (q, *J* = 7.2 Hz, 2H), 3.38 (d, *J* = 5.0 Hz, 2H), 3.02 (t, *J* = 5.8 Hz, 2H), 2.35 (s, 3H), 1.33 (t, *J* = 7.2 Hz, 3H); ^13^C NMR (75 MHz, DMSO-*d*_6_) δ 155.10, 148.46, 135.99, 135.12, 123.81, 108.66, 44.00, 39.90, 39.64, 18.33, 14.98, 10.53. LCMS (ESI): *m*/*z* (M + H^+^) calcd, 233.2; found, 233.4.

*3-(5-methyl-1,3-oxazol-2-yl)-1-(propan-2-yl)-4,5,6,7-tetrahydro-1H-pyrazolo[4,3-c]pyridine hydrochloride* (**9c**). Prepared using **6c** as described for **6a**. Yield 0.77 g (35% over 3 steps, approx. 14% dioxane), white solid, m.p. 261–266 °C. ^1^H NMR (300 MHz, DMSO-*d*_6_) δ 9.96 (s, 2H), 6.97 (s, 1H), 4.54 (q, *J* = 6.4 Hz, 1H), 4.21 (s, 2H), 3.37 (s, 2H), 3.04 (t, *J* = 5.3 Hz, 2H), 2.36 (s, 3H), 1.39 (d, *J* = 6.5 Hz, 6H); ^13^C NMR (75 MHz, DMSO-*d*_6_) δ 155.19, 148.38, 135.48, 135.04, 123.77, 108.50, 50.34, 39.87, 39.62, 22.17, 18.42, 10.54. LCMS (ESI): *m*/*z* (M + H^+^) calcd, 247.2; found, 247.2.

*3-(5-methyl-1,3-oxazol-2-yl)-1-(2-methylpropyl)-4,5,6,7-tetrahydro-1H-pyrazolo[4,3-c]pyridine hydrochloride* (**9d**). Prepared using **6d** as described for **6a**. Yield 0.85 g (39% over 3 steps, approx. 6% dioxane), white solid, m.p. 121–122 °C. ^1^H NMR (300 MHz, D_2_O) δ 7.01 (s, 1H), 4.49 (s, 2H), 3.95 (d, *J* = 7.5 Hz, 2H), 3.62 (t, *J* = 6.0 Hz, 2H), 3.13 (t, *J* = 6.0 Hz, 2H), 2.40 (s, 3H), 2.19 (hept, *J* = 6.6 Hz, 1H), 0.88 (d, *J* = 6.5 Hz, 6H); ^13^C NMR (75 MHz, D_2_O) δ 155.61, 151.58, 137.84, 134.99, 121.98, 109.00, 57.13, 41.55, 40.95, 29.66, 19.45, 19.39, 10.59. LCMS (ESI): *m*/*z* (M + H^+^) calcd, 261.2; found, 261.4.

#### 3.1.3. General Procedure for the Synthesis of Compounds **11e**–**h**

*tert-butyl 1-(cyclopropylmethyl)-3-formyl-1,4,6,7-tetrahydro-5H-pyrazolo[4,3-c]pyridine-5-carboxylate* (**11e**). A total of 5 g (14.3 mmol) of ester **6e** was dissolved in 50 mL of anhydrous tetrahydrofuran and LiAlH_4_ (13.1 mmol, 0.5 g) was added in portions at 0 °C. The reaction progress was monitored by TLC (chloroform/methanol 98:2) and it took about 1 h. After the reaction was completed, 0.5 mL of H_2_O, 0.5 mL of 15% NaOH aqueous solution, and 1.5 mL of H_2_O were successively added dropwise. The mixture was stirred for 2 h. The formed precipitate was filtered off, the filtrate was evaporated under vacuum. The residue was dissolved in 25 mL of dry methylene chloride and 2.5 g (28.7 mmol) of MnO_2_ was added. The mixture was stirred overnight, then filtered, and the solvent was evaporated under vacuum. Column chromatography on silica gel using 0 → 5% MeOH in CHCl_3_ as eluent afforded the corresponding aldehyde **11e**. Yield 2.66 g (61%), clear oil. ^1^H NMR (300 MHz, DMSO-*d*_6_) δ 9.87 (s, 1H), 4.50 (s, 2H), 4.01 (d, *J* = 7.1 Hz, 2H), 3.62 (t, *J* = 5.7 Hz, 2H), 2.76 (t, *J* = 5.7 Hz, 2H), 1.42 (s, 9H), 1.29–1.20 (m, 1H), 0.56–0.48 (m, 2H), 0.41–0.33 (m, 2H); ^13^C NMR (75 MHz, DMSO-*d*_6_) δ 187.24, 154.01, 145.09, 138.88, 114.98, 79.30, 53.77, 40.59, 28.02, 22.76, 21.33, 10.95, 3.66. LCMS (ESI): *m*/*z* (M + H^+^) calcd, 306.2; found, 306.4.

*tert-butyl 3-formyl-1-propyl-1,4,6,7-tetrahydro-5H-pyrazolo[4,3-c]pyridine-5-carboxylate* (**11f**). Yield 2.43 g (58%), clear oil. ^1^H NMR (300 MHz, DMSO-*d*_6_) δ 9.85 (s, 1H), 4.49 (s, 2H), 4.07 (t, *J* = 7.1 Hz, 2H), 3.62 (t, *J* = 5.7 Hz, 2H), 2.73 (t, *J* = 5.6 Hz, 2H), 1.79 (h, *J* = 7.3 Hz, 2H), 1.41 (s, 9H), 0.85 (t, *J* = 7.4 Hz, 3H). ^13^C NMR (75 MHz, DMSO-*d*_6_) δ 187.22, 154.01, 145.18, 139.12, 114.84, 79.40, 50.77, 40.95, 28.02, 21.35, 11.28. LCMS (ESI): *m*/*z* (M + H^+^) calcd, 294.2; found, 294.4.

*tert-butyl 3-formyl-1-(2-methoxyethyl)-1,4,6,7-tetrahydro-5H-pyrazolo[4,3-c]pyridine-5-carboxylate* (**11g**). Yield 2.12 g (48%), clear oil. ^1^H NMR (300 MHz, DMSO-*d*_6_) δ 9.86 (s, 1H), 4.49 (s, 2H), 4.29 (t, *J* = 5.1 Hz, 2H), 3.69 (t, *J* = 5.1 Hz, 2H), 3.61 (t, *J* = 5.7 Hz, 2H), 3.21 (s, 3H), 2.74 (t, *J* = 5.5 Hz, 2H), 1.41 (s, 9H).; ^13^C NMR (75 MHz, DMSO-*d*_6_) δ 187.20, 154.02, 145.35, 139.99, 114.74, 79.27, 70.42, 58.10, 49.31, 40.63, 27.99, 21.34. LCMS (ESI): *m*/*z* (M + H^+^) calcd, 310.2; found, 310.2.

*tert-butyl 3-formyl-1-(3-methoxypropyl)-1,4,6,7-tetrahydro-5H-pyrazolo[4,3-c]pyridine-5-carboxylate* (**11h**). Yield 1.66 g (36%), clear oil. ^1^H NMR (300 MHz, DMSO-*d*_6_) δ 9.86 (s, 1H), 4.49 (s, 2H), 4.15 (t, *J* = 7.0 Hz, 2H), 3.62 (t, *J* = 5.7 Hz, 2H), 3.28 (t, *J* = 6.0 Hz, 2H), 3.22 (s, 3H), 2.72 (t, *J* = 5.6 Hz, 2H), 2.01 (p, *J* = 6.5 Hz, 2H), 1.42 (s, 9H); ^13^C NMR (75 MHz, DMSO-*d*_6_) δ 187.23, 154.02, 145.27, 139.44, 114.79, 79.28, 68.46, 57.87, 46.27, 40.63, 29.21, 28.00, 21.02. LCMS (ESI): *m*/*z* (M + H^+^) calcd, 324.2; found, 324.6.

#### 3.1.4. General Procedure for the Synthesis of Compounds **12e**–**h**

To a solution of aldehyde **11** (3.2 mmol) in 25 mL of dry MeOH was added 1.81 g (13 mmol) of K_2_CO_3_ and TosMic (0.83 g, 4.2 mmol) and stirred for 6 h at reflux. The reaction mixture was poured into 100 mL of water and extracted with EtOAc (2 × 50 mL). The organic phases were combined and washed with 5% aqueous citric acid solution (2 × 50 mL) and 10% aqueous K_2_CO_3_ solution (2 × 50 mL). The organic phase was dried over anhydrous Na_2_SO_4_ and evaporated under vacuum. Column chromatography on silica gel using 0 → 5% MeOH in CHCl_3_ as eluent afforded target oxazoles. Fractions containing the target compound were combined and evaporated under vacuum. The residue was dissolved in 10 mL of 1,4-dioxane and a 4N solution of HCl in 1,4-dioxane was added dropwise. The precipitate that formed was filtered off, washed with ether, and dried.

*1-(cyclopropylmethyl)-3-(1,3-oxazol-5-yl)-4,5,6,7-tetrahydro-1H-pyrazolo[4,3-c]pyridine hydrochloride* (**12e**). Yield 0.43 g (48%), white solid, m.p. 126–127 °C. ^1^H NMR (300 MHz, DMSO-*d*_6_) δ 8.29 (s, 1H), 7.36 (s, 1H), 4.43 (s, 2H), 3.98 (d, *J* = 7.0 Hz, 2H), 3.61 (t, *J* = 6.2 Hz, 2H), 3.13 (t, *J* = 6.1 Hz, 2H), 1.27–1.19 (m, 1H), 0.63–0.54 (m, 2H), 0.40–0.32 (m, 2H); ^13^C NMR (75 MHz, DMSO-*d*_6_) δ 151.65, 145.28, 135.95, 135.22, 122.10, 107.01, 53.22, 40.08, 39.13, 18.71, 11.37, 3.79. LCMS (ESI): *m*/*z* (M + H^+^) calcd, 245.1; found, 245.2.

*3-(1,3-oxazol-5-yl)-1-propyl-4,5,6,7-tetrahydro-1H-pyrazolo[4,3-c]pyridine hydrochloride* (**12f**). Yield 0.45 g (45%, approx. 13% dioxane), white solid, m.p. 142–143 °C. ^1^H NMR (300 MHz, DMSO-*d*_6_) δ 10.00 (s, 2H), 8.44 (s, 1H), 7.42 (s, 1H), 4.22 (s, 2H), 4.03 (t, *J* = 6.9 Hz, 2H), 3.37 (d, *J* = 5.9 Hz, 2H), 3.01 (t, *J* = 5.7 Hz, 2H), 1.75 (h, *J* = 7.2 Hz, 2H), 0.85 (t, *J* = 7.4 Hz, 3H).; ^13^C NMR (75 MHz, DMSO-*d*_6_) δ 151.56, 145.25, 136.24, 135.21, 122.05, 106.70, 50.29, 40.07, 39.12, 23.00, 18.55, 10.99. LCMS (ESI): *m*/*z* (M + H^+^) calcd, 233.1; found, 233.4.

*1-(2-methoxyethyl)-3-(1,3-oxazol-5-yl)-4,5,6,7-tetrahydro-1H-pyrazolo[4,3-c]pyridine hydrochloride* (**12g**). Yield 0.35 g (30%, approx. 20% dioxane), white solid, m.p. 119–120 °C. ^1^H NMR (300 MHz, DMSO-*d*_6_) δ 10.03 (s, 2H), 8.46 (s, 1H), 7.43 (s, 1H), 4.28–4.18 (m, 4H), 3.65 (t, *J* = 5.0 Hz, 2H), 3.35 (d, *J* = 4.5 Hz, 2H), 3.20 (s, 3H), 3.01 (t, *J* = 5.7 Hz, 2H); ^13^C NMR (75 MHz, DMSO-*d*_6_) δ 151.95, 145.54, 137.39, 135.85, 122.53, 107.14, 71.05, 58.65, 49.34, 40.38, 39.34, 18.97. LCMS (ESI): *m*/*z* (M + H^+^) calcd, 249.1; found, 249.2.

*1-(3-methoxypropyl)-3-(1,3-oxazol-5-yl)-4,5,6,7-tetrahydro-1H-pyrazolo[4,3-c]pyridine hydrochloride* (**12h**). Yield 0.4 g (36%, approx. 14% dioxane), white solid, m.p. 84–85 °C. ^1^H NMR (300 MHz, DMSO-*d*_6_) δ 10.12 (s, 2H), 8.45 (s, 1H), 7.42 (s, 1H), 4.20 (s, 2H), 4.08 (t, *J* = 6.7 Hz, 2H), 3.23 (t, *J* = 6.0 Hz, 2H), 3.21 (s, 3H), 2.99 (t, *J* = 5.4 Hz, 2H), 1.99–1.90 (m, 2H); ^13^C NMR (75 MHz, DMSO-*d*_6_) δ 151.70, 145.31, 136.61, 135.53, 122.19, 106.76, 68.49, 58.13, 45.82, 40.22, 39.24, 29.69, 18.52. LCMS (ESI): *m*/*z* (M + H^+^) calcd, 263.2; found, 263.4.

#### 3.1.5. General Procedure for the Synthesis of Compounds **10a**–**d** and **13e**–**h**

To a solution of 5-nitro-2-furoic acid (0.1 g, 0.6 mmol) in dry DMF (5 mL) CDI (0.12 g, 0.70 mmol) was added and the mixture was stirred at r.t. for 30 min. This one was added dropwise to the mixture of hydrochloride **9** (for **10** synthesis) or **12** (for **13**) (0.7 mmol) and triethylamine (0.1 mL, 0.8 mmol) in dry DMF (5 mL) and the stirring continued for 18 h. The resulting mixture was poured into water (30 mL) and extracted with ethyl acetate (3 × 50 mL). The organic phase was successively washed with 10% aqueous K_2_CO_3_ (2×10 mL) and dried over anhydrous Na_2_SO_4_, filtered, and concentrated in vacuo. The residue was suspended in diethyl ether and filtered, then dried under vacuum.

*1-methyl-3-(5-methyl-1,3-oxazol-2-yl)-5-(5-nitro-2-furoyl)-4,5,6,7-tetrahydro-1H-pyrazolo [4,3-c]pyridine, LK01510* (**10a**). Yield 0.121 g (54%), brown solid, m.p. 245 ÷ 247 °C. ^1^H NMR (300 MHz, DMSO-*d*_6_) δ 7.79 (br.s, 1H), 7.34 (br.s, 1H), 6.95 (br.s, 1H), 5.02–4.66 (m, 2H), 3.95 (s, 2H), 3.79 (s, 3H), 2.92 (s, 2H), 2.35 (s, 3H); ^13^C NMR (75 MHz, DMSO-*d*_6_) δ 157.66, 155.34, 151.31, 148.14, 147.54, 138.55, 134.60, 123.80, 117.03, 112.94, 112.05, 43.35, 36.16, 21.95, 21.03, 10.51. HRMS (ESI), *m*/*z* calcd C_16_H_15_N_5_O_5_Na [M + Na^+^] 380.0966, found 380.0965.

*1-ethyl-3-(5-methyl-1,3-oxazol-2-yl)-5-(5-nitro-2-furoyl)-4,5,6,7-tetrahydro-1H-pyrazolo [4,3-c]pyridine, LK01511* (**10b**). Yield 0.124 g (52%), brown solid, m.p. 180 ÷ 181 °C. ^1^H NMR (300 MHz, DMSO-*d*_6_) δ 7.80 (d, *J* = 2.8 Hz, 1H), 7.36 (d, *J* = 2.8 Hz, 1H), 6.97 (s, 1H), 5.02–4.70 (m, 2H), 4.12 (q, *J* = 6.4 Hz, 2H), 3.96 (br.s, 2H), 2.97 (br.s, 2H), 2.36 (s, 3H), 1.37 (t, *J* = 5.8 Hz, 3H); ^13^C NMR (75 MHz, DMSO-*d*_6_) ^13^C NMR (75 MHz, DMSO) δ 157.68, 155.51, 151.36, 148.25, 147.63, 137.57, 134.78, 125.10, 118.25, 114.21, 111.75, 45.74, 43.91, 22.13, 20.23, 14.27, 9.71. HRMS (ESI), *m*/*z* calcd C_17_H_17_N_5_O_5_Na [M + Na^+^] 394.1122, found 394.1122.

*1-isopropyl-3-(5-methyl-1,3-oxazol-2-yl)-5-(5-nitro-2-furoyl)-4,5,6,7-tetrahydro-1H-pyrazolo [4,3-c]pyridine, LK01515* (**10c**). Yield 0.113 g (46%), brown solid, m.p. 194 ÷ 196 °C. ^1^H NMR (300 MHz, DMSO-*d*_6_) δ 7.81 (d, *J* = 3.8 Hz, 1H), 7.36 (d, *J* = 3.8 Hz, 1H), 6.98 (s, 1H), 5.01–4.70 (m, 2H), 4.60–4.47 (m, 1H), 3.96 (br.s, 2H), 3.07–2.84 (m, 2H), 2.37 (s, 3H), 1.42 (d, *J* = 6.2 Hz, 6H); ^13^C NMR (75 MHz, DMSO-*d*_6_) δ 157.62, 155.52, 151.31, 148.09, 147.62, 136.95, 134.68, 123.77, 116.94, 112.92, 111.72, 50.18, 43.97, 43.66, 22.24, 22.07, 20.76, 10.54. HRMS (ESI), *m*/*z* calcd C_18_H_19_N_5_O_5_Na [M + Na^+^] 408.1279, found 408.1278.

*1-isobutyl-3-(5-methyl-1,3-oxazol-2-yl)-5-(5-nitro-2-furoyl)-4,5,6,7-tetrahydro-1H-pyrazolo [4,3-c]pyridine, LK01516* (**10d**). Yield 0.130 g (51%), brown solid, m.p. 123 ÷ 124 °C. ^1^H NMR (300 MHz, DMSO-*d*_6_) δ 7.81 (d, *J* = 3.9 Hz, 1H), 7.37 (d, *J* = 3.9 Hz, 1H), 6.99 (s, 1H), 5.03–4.71 (m, 2H), 3.95 (t, *J* = 5.3 Hz, 2H), 3.90 (d, *J* = 6.9 Hz, 2H), 3.03–2.79 (m, 2H), 2.36 (s, 3H), 2.21–2.07 (m, 1H), 0.87 (d, *J* = 6.5 Hz, 6H); ^13^C NMR (75 MHz, DMSO-*d*_6_) δ 157.62, 155.37, 151.29, 148.15, 147.63, 138.13, 134.87, 123.80, 117.06, 112.92, 111.64, 55.87, 43.69, 28.98, 22.36, 21.05, 19.67, 10.51. HRMS (ESI), *m*/*z* calcd C_19_H_21_N_5_O_5_Na [M + Na^+^] 422.1435, found 422.1435.

*1-(cyclopropylmethyl)-5-(5-nitro-2-furoyl)-3-(1,3-oxazol-5-yl)-4,5,6,7-tetrahydro-1H-pyrazolo [4,3-c]pyridine, LK01512* (**13e**). Yield 0.152 g (62%), brown solid, m.p. 144 ÷ 145 °C. ^1^H NMR (300 MHz, DMSO-*d*_6_) δ 8.43 (s, 1H), 7.79 (d, *J* = 3.6 Hz, 1H), 7.38 (s, 2H), 5.01–4.69 (m, 2H), 4.01–3.93 (m, 4H), 2.97 (s, 2H), 1.31–1.14 (m, 1H), 0.57–0.47 (m, 2H), 0.41–0.33 (m, 2H); ^13^C NMR (75 MHz, DMSO-*d*_6_) δ 157.60, 151.37, 147.55, 145.69, 137.38, 134.79, 121.65, 117.55, 117.05, 112.97, 110.15, 53.18, 43.77, 43.22, 22.36, 21.15, 11.33, 3.70. HRMS (ESI), *m*/*z* calcd C_18_H_17_N_5_O_5_Na [M + Na^+^] 406.1122, found 406.1130.

*5-(5-nitro-2-furoyl)-3-(1,3-oxazol-5-yl)-1-propyl-4,5,6,7-tetrahydro-1H-pyrazolo[4,3-c]pyridine, LK01513* (**13f**). Yield 0.137 g (58%), brown solid, m.p. 149 ÷ 150 °C. ^1^H NMR (300 MHz, DMSO-*d*_6_) δ 8.42 (s, 1H), 7.78 (s, 1H), 7.38 (s, 2H), 5.04–4.64 (m, 2H), 4.11–3.90 (m, 4H), 2.94 (br.s, 2H), 1.78 (br.s, 2H), 0.86 (br.s, 3H); ^13^C NMR (75 MHz, DMSO-*d*_6_) δ 157.65, 151.34, 147.53, 145.62, 137.75, 134.75, 121.61, 117.58, 117.06, 112.94, 109.91, 50.21, 43.74, 22.94, 22.23, 21.01, 11.00. HRMS (ESI), *m*/*z* calcd C_17_H_17_N_5_O_5_Na [M + Na^+^] 394.1122, found 394.1128.

*1-(2-methoxyethyl)-5-(5-nitro-2-furoyl)-3-(1,3-oxazol-5-yl)-4,5,6,7-tetrahydro-1H-pyrazolo [4,3-c]pyridine, LK01509* (**13g**). Yield 0.104 g (42%), brown solid, m.p. 130 ÷ 131 °C. ^1^H NMR (300 MHz, DMSO-*d*_6_) δ 8.43 (s, 1H), 7.78 (d, *J* = 3.7 Hz, 1H), 7.38 (s, 2H), 5.03–4.66 (m, 2H), 4.24 (t, *J* = 4.4 Hz, 2H), 3.95 (br.s, 2H), 3.68 (t, *J* = 4.5 Hz, 2H), 3.23 (s, 3H), 2.95 (br.s, 2H); ^13^C NMR (75 MHz, DMSO-*d*_6_) δ 157.68, 151.38, 147.55, 145.61, 138.58, 135.09, 121.73, 117.61, 117.08, 112.94, 109.95, 70.70, 58.16, 48.75, 43.71, 22.36, 21.13. HRMS (ESI), *m*/*z* calcd C_17_H_17_N_5_O_6_ Na [M + Na^+^] 410.1072, found 410.1071.

*1-(3-methoxypropyl)-5-(5-nitro-2-furoyl)-3-(1,3-oxazol-5-yl)-4,5,6,7-tetrahydro-1H-pyrazolo [4,3-c]pyridine, LK01514* (**13h**). Yield 0.100 g (39%), brown solid, m.p. 97÷98 °C. ^1^H NMR (300 MHz, DMSO-*d*_6_) δ 8.42 (s, 1H), 7.78 (d, *J* = 3.3 Hz, 1H), 7.38 (s, 2H), 5.00–4.66 (m, 2H), 4.11 (t, *J* = 5.8 Hz, 2H), 3.96 (br.s, 2H), 3.28 (t, *J* = 6.0 Hz, 2H), 3.23 (s, 3H), 2.91 (br.s, 2H), 2.06–1.92 (m, 2H).; ^13^C NMR (75 MHz, DMSO-*d*_6_) δ 157.63, 151.34, 147.55, 145.66, 137.99, 135.00, 121.67, 117.55, 117.05, 112.91, 109.91, 68.55, 57.92, 45.65, 43.71, 29.53, 22.13, 20.81. HRMS (ESI), *m*/*z* calcd C_18_H_19_N_5_O_6_Na [M + Na^+^] 424.1228, found 424.1228.

### 3.2. Biological Activity Evaluation

Testing was conducted against the following microorganisms: *Enterococcus faecalis* (ATCC 29812), *Staphylococcus aureus* (ATCC 25912), *Klebsiella pneumoniae* (ATCC 19882), *Acinetobacter baumannii* (948^®^, patient-derived strain from the Pasteur Institute’s own collection), *Pseudomonas aeruginosa* (ATCC 27853), and *Enterobacter cloacae* (ATCC 13047) for compounds **10a**–**d**, **13e**–**h**, nitrofurantoin, and ciprofloxacin (employed as a positive control) using the Kirby–Bauer disk diffusion test [24] under the Standard Operating Procedure of The European Committee on Antimicrobial Susceptibility Testing (EUCAST) [25]. Paper disks bearing 5 mg of the tested compounds were used. Solutions of the tested compounds made up in DMSO (1 mg/10 mL) were prepared and diluted to a total volume of 1 mL with deionized water. Aliquots of the resulting solutions (5 µL each) were added to a Petri dish containing Muller–Hilton agar that was inoculated with a bacterial suspension (McFarland OD 1/4 0.5). After the compound solution dried off, the Petri dish was incubated at 37 °C for 18 h. The bacterial growth inhibition zone diameter around the disc with ciprofloxacin or the compounds’ dried solution circular spot indicated the general susceptibility to a drug being assessed. Thereupon, minimum inhibitory concentrations (MIC, µg/mL) were determined using serial broth dilutions [26]. All measurements were carried out in triplicate.

### 3.3. In Silico Studies

#### 3.3.1. Target Selection

As the potential targets for the observed nitrofuran derivatives **13a**–**m**, the following two proteins were selected: *Escherichia coli* nitroreductase NfsB (PDB id: 1YKI) and oxygen insensitive NADPH nitroreductase NfsA (PDB id: 7NB9). These proteins have been investigated as molecular targets for nitrofuran-based drugs. The main reference interactants in this case are nitrofurazone [27] or nitrofurantoin [28].

#### 3.3.2. Protein and Ligand Structure Preparation

All proteins were downloaded from the RCSB protein data bank and prepared using the Schrodinger Protein prepwizard. This phase corrects invalid bond ordering, protonation, missing residues, and residue sidechains.

The geometry of ligands was created by the LigPrep module. All molecular modifications were carried out in the OPLS4 forcefield [29]. Schrodinger Suite 2022-4 was used for calculations.

#### 3.3.3. Docking of Molecules

The docking grid box was calculated on the basis of reference ligand positioning and size (grid placement on complexed ligand centroid, maximum grid side size is 12 Å). The flexibility of amino acids is considered within a radius of 5 Å from the ligand atoms (not the centroid!). The best-fitting pose was chosen manually by assessing the repeatability of contacts of the nitrofuran moiety in the reference ligand in the protein active site (present in the PDB files).

#### 3.3.4. Molecular Mechanics with Generalized Born and Surface Area Solvation (MM-GBSA)

Gibbs free energy (ΔG) was estimated for each ligand binding pose in the presence of an implicit solvent. Calculations were carried out using the MM-GBSA method. The values shown in Figure 2 are associated with a green-to-red gradient molecular coloring, where green represents the minimum and red represents the maximum per-atom ligand strain value. Color ramping is adaptive for each structure (shown in the figures).

## 4. Conclusions

A series of nitrofuran derivatives has been prepared with excellent regioselectivity. The testing of these compounds against pathogens of the ESKAPE panel showed a good activity of lead compound 1-(2-methoxyethyl)-5-(5-nitro-2-furoyl)-3-(1,3-oxazol-5-yl)-4,5,6,7-tetrahydro-1*H*-pyrazolo[4,3-c] pyridine (13 g), which potentially can be useful as antibiotics of the next generation. These results confirmed the benefit of combining a THPP scaffold with a nitrofuran warhead. Certain structure–activity relationships were established in the course of this study which were rationalized by the induced-fit docking experiments in silico.

## Figures and Tables

**Table 1:** Antibacterial activity (disk diffusion method inhibition zone (IZ, mm) and minimal inhibitory concentration (MIC, µg/mL) of compounds **10a**–**d** and **13e**–**h**, ciprofloxacin, and nitrofurantoin (positive controls) against the ESKAPE panel of pathogens; nt—not tested. The active compounds are highlighted by green. The MIC values are means from three different assays (errors were in the range of ±5–10% of the reported values).

Compound	*E. faecium*		*S. aureus*		*K. pneumoniae*		*A. baumannii*		*P. aeruginosa*		*E. cloacae*	
	IZ	MIC	IZ	MIC	IZ	MIC	IZ	MIC	IZ	MIC	IZ	MIC
**LK01510 (10a)**	0	nt	0	nt	0	nt	0	nt	0	nt	0	nt
**LK01511 (10b)**	0	nt	0	nt	7	>150	0	nt	0	nt	0	nt
**LK01515 (10c)**	2	0	0	nt	0	nt	0	nt	0	nt	0	nt
**LK01516 (10d)**	0	nt	13	>150	0	nt	0	nt	0	nt	0	nt
**LK01512 (13e)**	0	nt	11	>150	0	nt	0	nt	0	nt	0	nt
**LK01513 (13f)**	0	16	13	150	7	75	0	150	0	150	0	150
**LK01509 (13g)**	19	150	7	4	8	32	0	16	0	75	6	16
**LK01514 (13h)**	0	150	21	150	0	150	0	32	0	75	0	150
**nitrofurantoin**	15	8	21	4	12	64	0	nt	0	nt	24	4
**ciprofloxacin**	17	1.25	17	1.25	13	0.6	14	2.5	9	0.6	21	3

**Table 2:** Docking and MM-GBSA Gibbs free energy values of observed compounds, docked into their potential targets; the best compound is highlighted by green.

Compound	System Energy (kcal/mol)		MM-GBSA ΔG (kcal/mol)		MM-GBSA Strain Energy (kcal/mol)		Glide Score (kcal/mol)	
	1YKI	7NB9	1YKI	7NB9	1YKI	7NB9	1YKI	7NB9
**Reference** **(nitrofurazone)**	−17,665.90	−19,712.29	−75.40	−47.54	3.63	1.23	−5.18	−5.58
**LK01513 (13f)**	−17,638.69	−19,776.54	−74.08	−40.29	5.63	8.47	−8.27	−5.29
**LK01509 (13g)**	−17,718.99	−19,808.87	−67.75	−53.65	2.46	5.39	−6.69	−5.52
**LK01514 (13h)**	−17,704.12	−19,768.31	−77.66	−36.85	4.10	9.07	−7.85	−5.22

## Data Availability

Not applicable.

## Supplementary Materials

The following supporting information can be downloaded at: https://www.mdpi.com/article/10.3390/molecules28186491/s1, Copies of ^1^H and ^13^C NMR spectra.

## Sample Availability

Samples of the compounds are available from the authors.
