# Supplementary material for: 5-Nitrofuran-Tagged Oxazolyl Pyrazolopiperidines: Synthesis and Activity against ESKAPE Pathogens
Source: Molecules. 2023 Sep 7;28(18):6491. doi: 10.3390/molecules28186491 (PMC10537382; doi:10.3390/molecules28186491)

Supporting information

5-Nitrofuran-Tagged Oxazolyl Pyrazolopiperidines: Synthesis and Activity against ESKAPE Pathogens

Elizaveta Rogacheva [1], Lyudmila Kraeva [1], Alexey Lukin [2], Lyubov Vinogradova [2], Kristina Komarova [2], Mikhail Chudinov [2,*], Maxim Gureev [3,4], and Evgeny Chupakhin [5]

[1] Pasteur Institute of Epidemiology and Microbiology, Saint Petersburg 197101, Russia
[2] Lomonosov Institute of Fine Chemical Technologies, MIREA—Russian Technological University, Moscow 119454, Russia
[3] Laboratory of Bio- and chemoinformatics, I. M. Sechenov First Moscow State Medical University, Moscow 119991, Russia
[4] Molecular modeling lab, HSE University, Saint-Petersburg, 190121, Russia
[5] Immanuel Kant Baltic Federal University, Kaliningrad 236041, Russia.
* Correspondence: chudinov@mirea.ru

NMR spectra of synthesized compounds

5-tert-butyl 3-ethyl 1-methyl-1,4,6,7-tetrahydro-5H-pyrazolo[4,3-c]pyridine-3,5- dicarboxylate (6a) 3

5-tert-butyl 3-ethyl 1-ethyl-1,4,6,7-tetrahydro-5H-pyrazolo[4,3-c]pyridine-3,5-dicarboxylate (6b) 4

5-tert-butyl-3-ethyl-1-(propan-2-yl)-1,4,6,7-tetrahydro-5H-pyrazolo[4,3-c]pyridine-3,5- dicarboxylate (6c)..........5

5-tert-butyl-3-ethyl-1-(2-methylpropyl)-1,4,6,7-tetrahydro-5H-pyrazolo[4,3-c]pyridine-3,5- dicarboxylate (6d) 6

5-tert-butyl-3-ethyl-1-(cyclopropylmethyl)-1,4,6,7-tetrahydro-5H-pyrazolo[4,3-c]pyridine-3,5-dicarboxylate (6e)..........7

5-tert-butyl-3-ethyl-1- propyl -1,4,6,7-tetrahydro-5H-pyrazolo[4,3-c]pyridine-3,5-dicarboxylate (6f) 8

5-tert-butyl-3-ethyl-1-(2-methoxyethyl)-1,4,6,7-tetrahydro-5H-pyrazolo[4,3-c]pyridine-3,5-dicarboxylate (6g)..........9

5-tert-butyl-3-ethyl-1-(2-methoxypropyl)-1,4,6,7-tetrahydro-5H-pyrazolo[4,3-c]pyridine-3,5-dicarboxylate (6h) 10

1-methyl-3-(5-methyl-1,3-oxazol-2-yl)-4,5,6,7-tetrahydro-1H-pyrazolo[4,3-c]pyridine hydrochloride .... (9a)11

1-ethyl-3-(5-methyl-1,3-oxazol-2-yl)-4,5,6,7-tetrahydro-1H-pyrazolo[4,3-c]pyridine hydrochloride (9b) 12

3-(5-methyl-1,3-oxazol-2-yl)-1-(propan-2-yl)-4,5,6,7-tetrahydro-1H-pyrazolo[4,3-c]pyridine hydrochloride (9c) 13

3-(5-methyl-1,3-oxazol-2-yl)-1-(2-methylpropyl)-4,5,6,7-tetrahydro-1H-pyrazolo[4,3-c]pyridine hydrochloride (9d)..........14

tert-butyl 1-(cyclopropylmethyl)-3-formyl-1,4,6,7-tetrahydro-5H-pyrazolo[4,3-c]pyridine-5- carboxylate (11e) 15

tert-butyl 3-formyl-1-propyl-1,4,6,7-tetrahydro-5H-pyrazolo[4,3-c]pyridine-5-carboxylate (11f) 16

tert-butyl 3-formyl-1-(2-methoxyethyl)-1,4,6,7-tetrahydro-5H-pyrazolo[4,3-c]pyridine-5-carboxylate (11g) 17

tert-butyl 3-formyl-1-(3-methoxypropyl)-1,4,6,7-tetrahydro-5H-pyrazolo[4,3-c]pyridine-5-carboxylate (11h) 18

1-(cyclopropylmethyl)-3-(1,3-oxazol-5-yl)-4,5,6,7-tetrahydro-1H-pyrazolo[4,3-c]pyridine hydrochloride (12e) 19

3-(1,3-oxazol-5-yl)-1-propyl-4,5,6,7-tetrahydro-1H-pyrazolo[4,3-c]pyridine hydrochloride (12f) 20

1-(2-methoxyethyl)-3-(1,3-oxazol-5-yl)-4,5,6,7-tetrahydro-1H-pyrazolo[4,3-c]pyridine hydrochloride (12g) 21

1-(3-methoxypropyl)-3-(1,3-oxazol-5-yl)-4,5,6,7-tetrahydro-1H-pyrazolo[4,3-c]pyridine hydrochloride (12h) 22

1-methyl-3-(5-methyl-1,3-oxazol-2-yl)-5-(5-nitro-2-furoyl)-4,5,6,7-tetrahydro-1H-pyrazolo [4,3-c]pyridine, LK01510 (10a) 23

1-ethyl-3-(5-methyl-1,3-oxazol-2-yl)-5-(5-nitro-2-furoyl)-4,5,6,7-tetrahydro-1H-pyrazolo [4,3-c]pyridine, LK01511 (10b) 24

1-isopropyl-3-(5-methyl-1,3-oxazol-2-yl)-5-(5-nitro-2-furoyl)-4,5,6,7-tetrahydro-1H-pyrazolo [4,3-c]pyridine, LK01515 (10c) 25

1-isobutyl-3-(5-methyl-1,3-oxazol-2-yl)-5-(5-nitro-2-furoyl)-4,5,6,7-tetrahydro-1H-pyrazolo [4,3-c]pyridine, LK01516 (10d) 26

1-(cyclopropylmethyl)-5-(5-nitro-2-furoyl)-3-(1,3-oxazol-5-yl)-4,5,6,7-tetrahydro-1H-pyrazolo [4,3-c]pyridine, LK01512 (13e) 27

5-(5-nitro-2-furoyl)-3-(1,3-oxazol-5-yl)-1-propyl-4,5,6,7-tetrahydro-1H-pyrazolo[4,3-c]pyridine, LK01513 (13f) 28

1-(2-methoxyethyl)-5-(5-nitro-2-furoyl)-3-(1,3-oxazol-5-yl)-4,5,6,7-tetrahydro-1H-pyrazolo [4,3-c]pyridine, LK01509 (13g) 29

1-(3-methoxypropyl)-5-(5-nitro-2-furoyl)-3-(1,3-oxazol-5-yl)-4,5,6,7-tetrahydro-1H-pyrazolo [4,3-c]pyridine, LK01514 (13h) 30

5-tert-butyl 3-ethyl 1-methyl-1,4,6,7-tetrahydro-5H-pyrazolo[4,3-c]pyridine-3,5- dicarboxylate (6a)

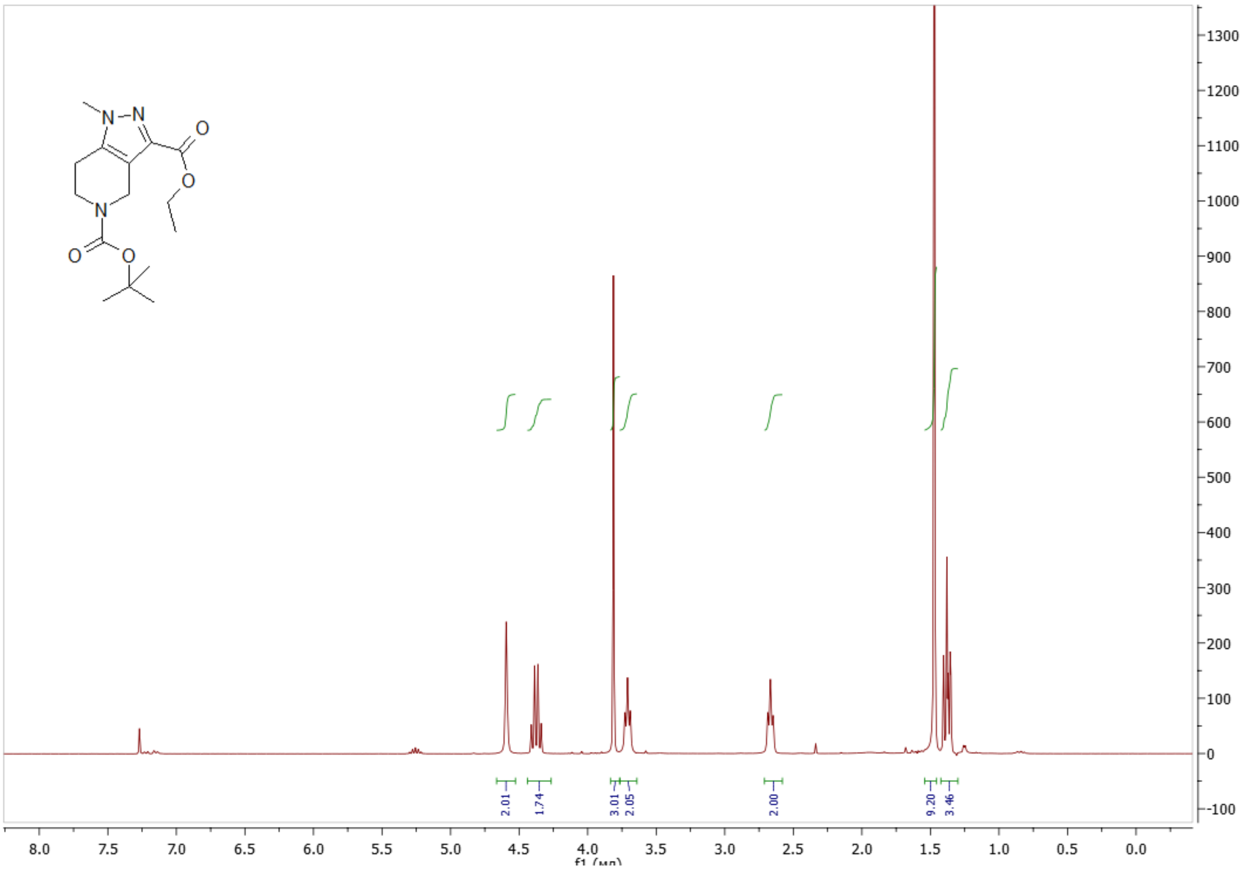

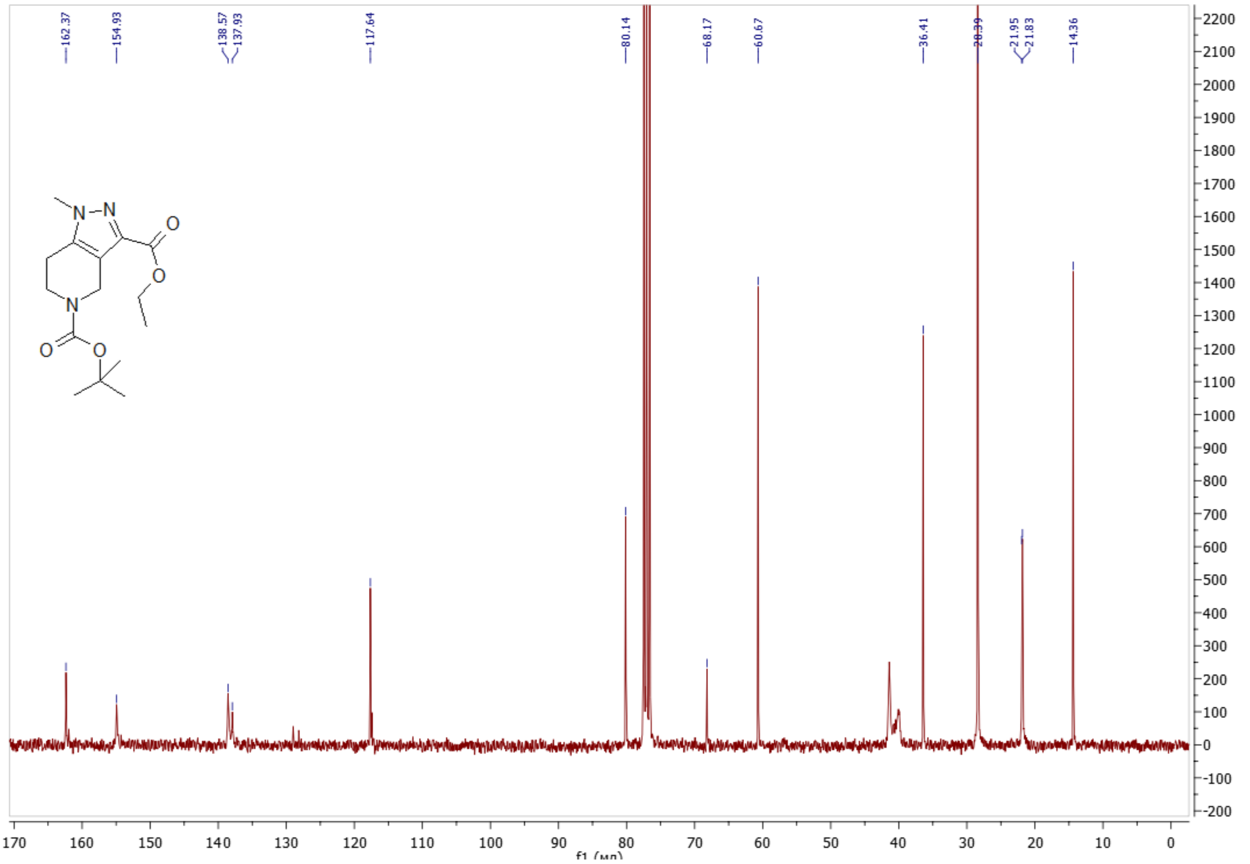

5-tert-butyl 3-ethyl 1-ethyl-1,4,6,7-tetrahydro-5H-pyrazolo[4,3-c]pyridine-3,5-dicarboxylate (6b)

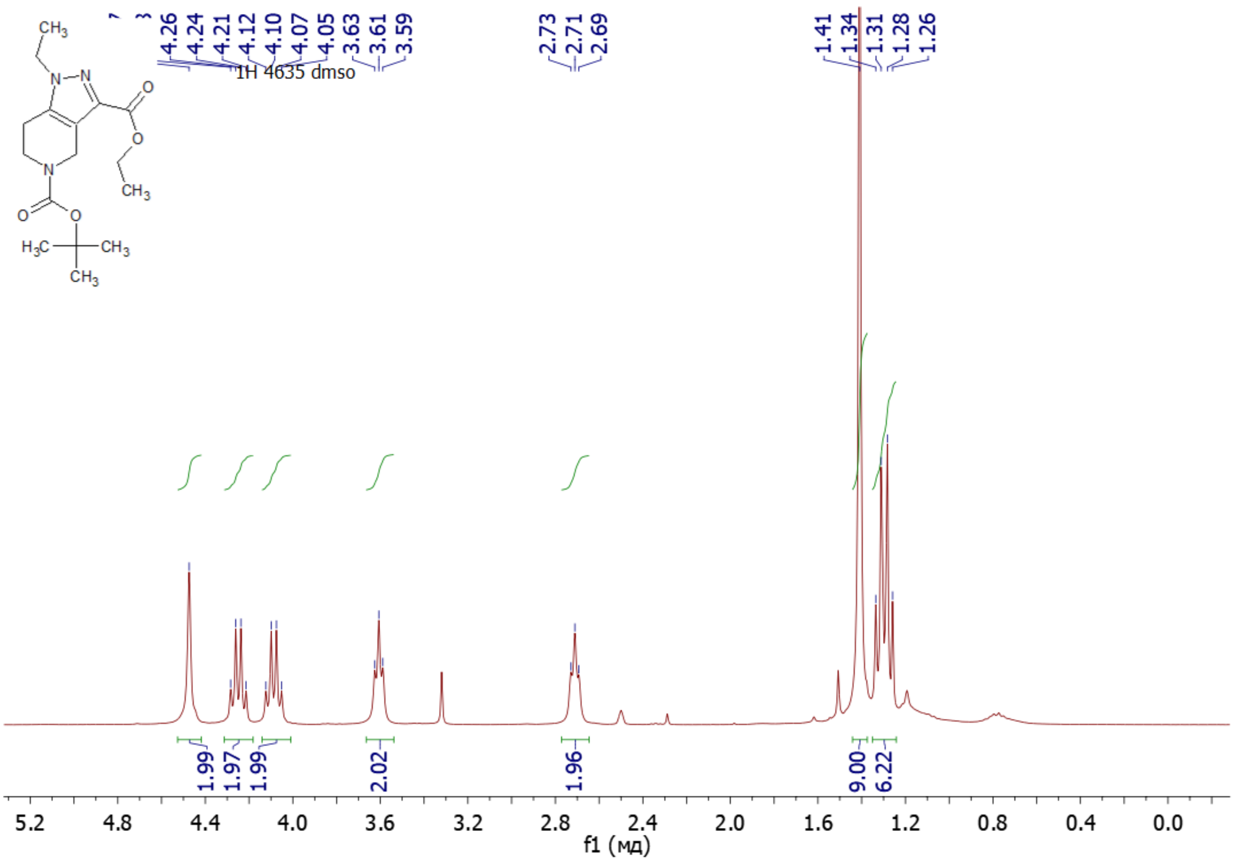

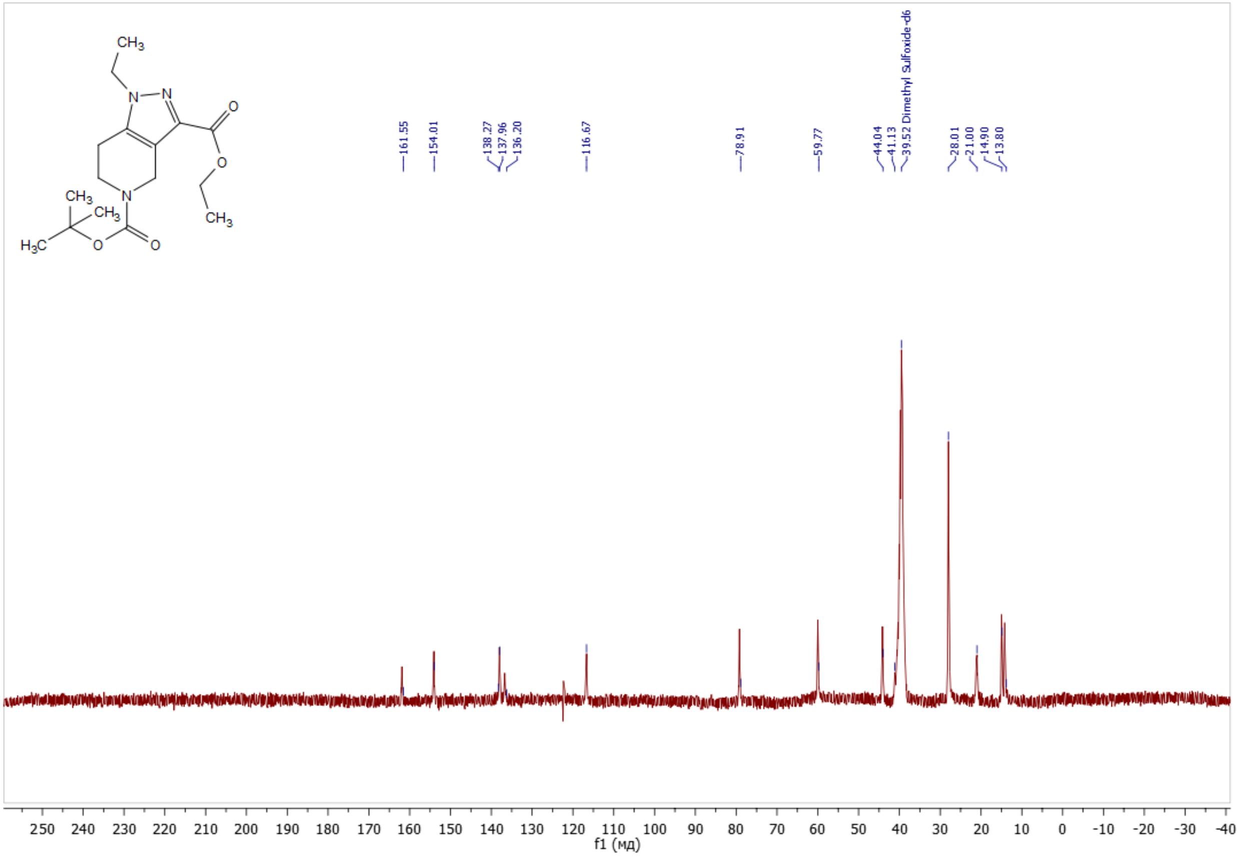

5-tert-butyl-3-ethyl-1-(propan-2-yl)-1,4,6,7-tetrahydro-5H-pyrazolo[4,3-c]pyridine-3,5- dicarboxylate (6c)

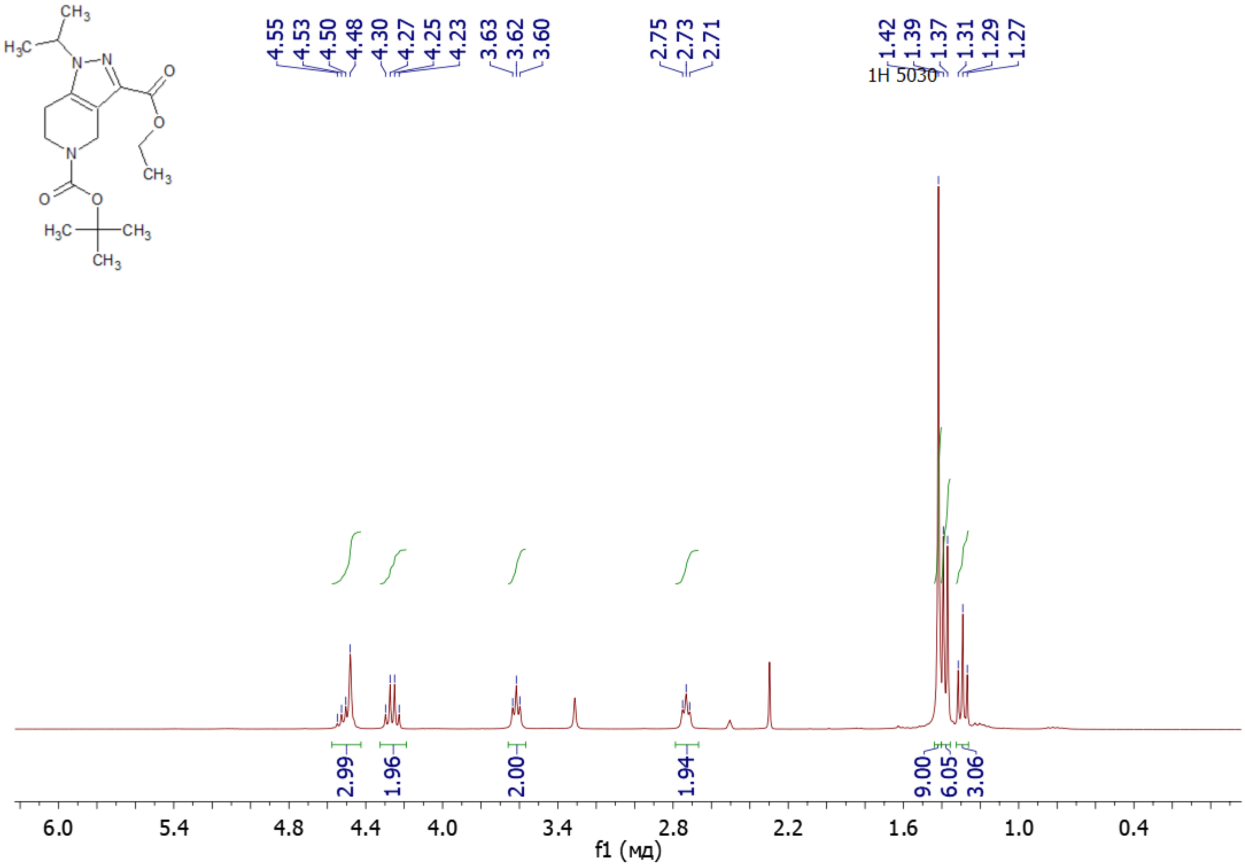

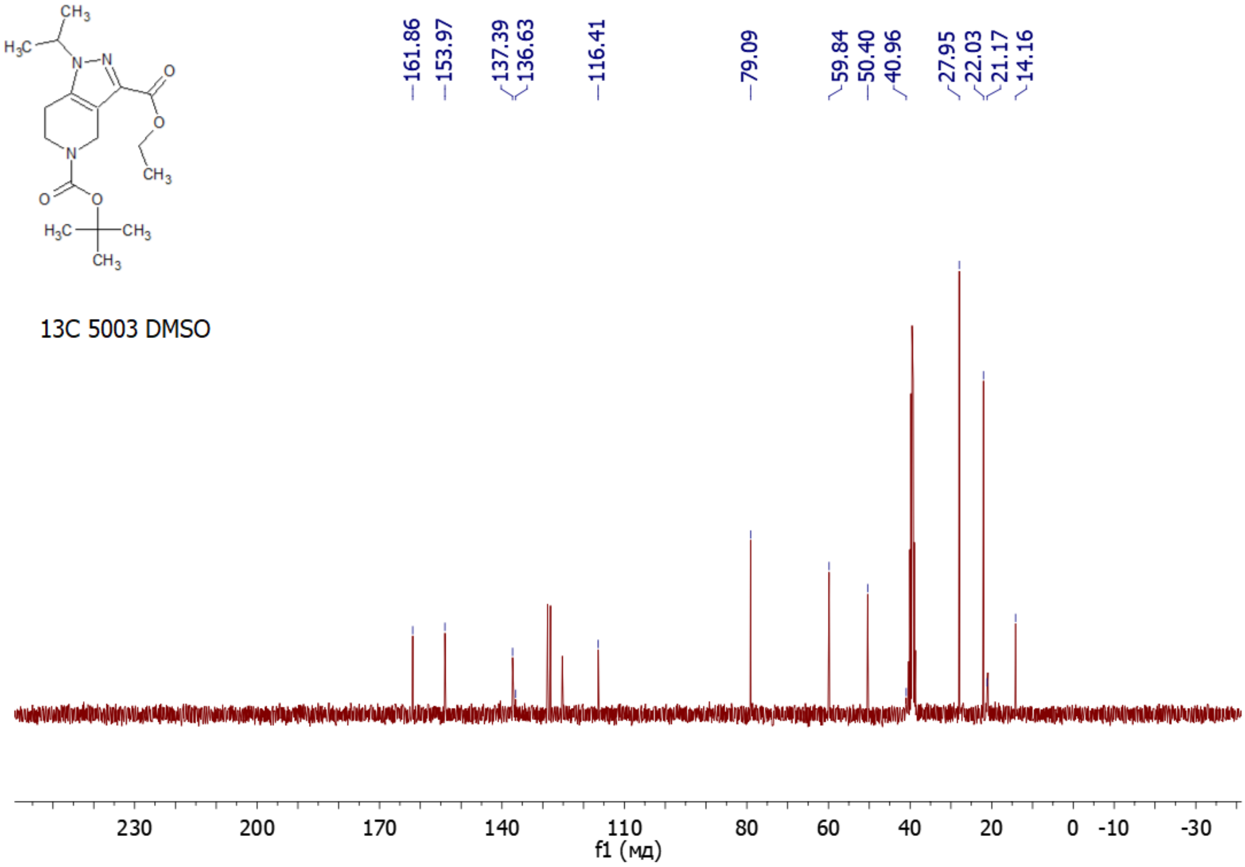

5-tert-butyl-3-ethyl-1-(2-methylpropyl)-1,4,6,7-tetrahydro-5H-pyrazolo[4,3-c]pyridine-3,5- dicarboxylate (6d)

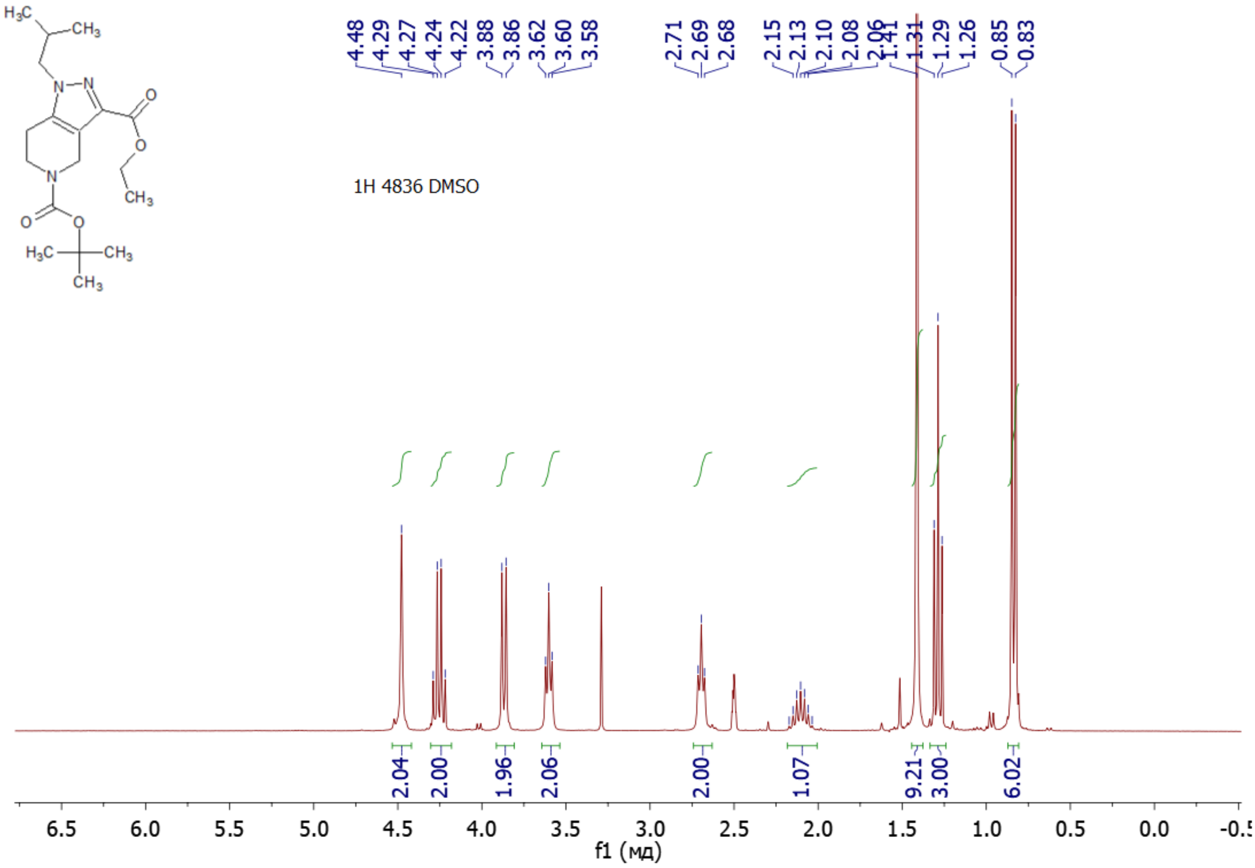

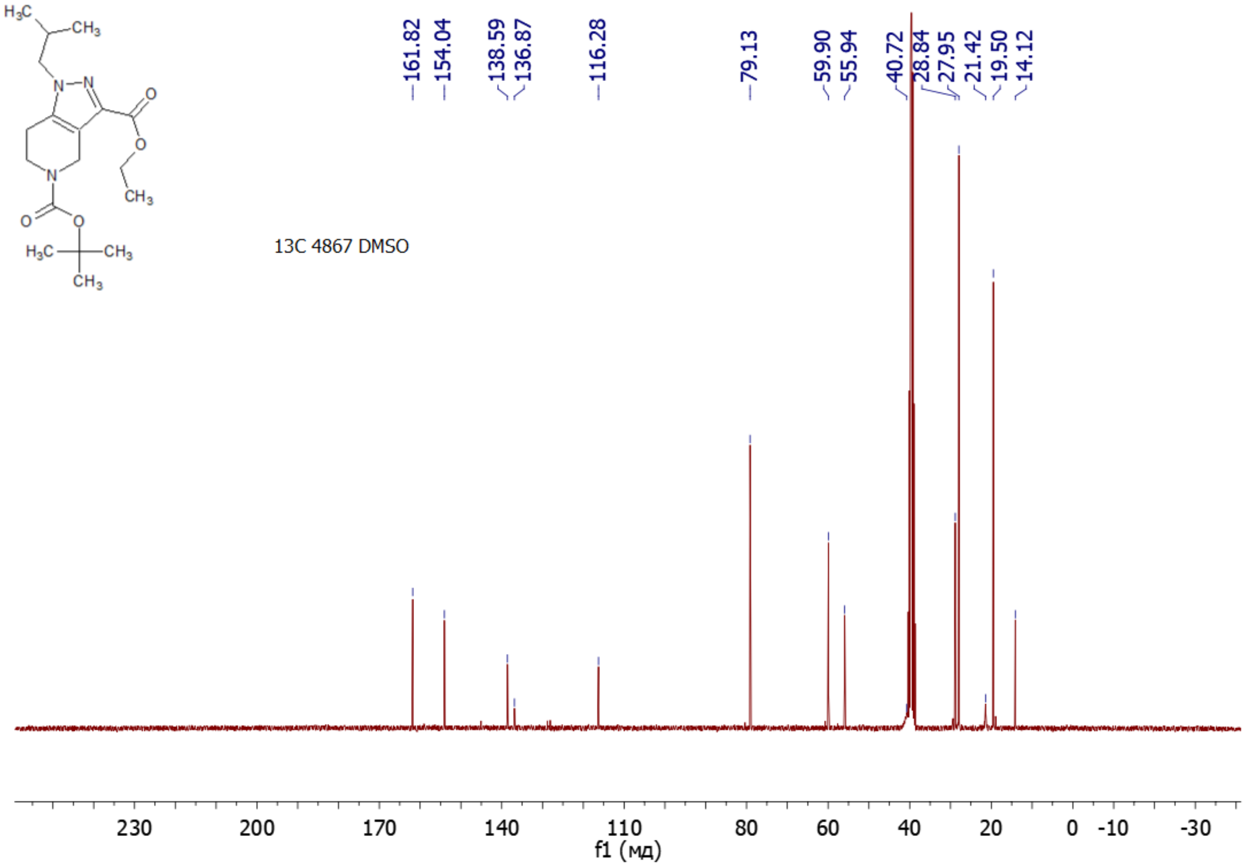

5-tert-butyl-3-ethyl-1-(cyclopropylmethyl)-1,4,6,7-tetrahydro-5H-pyrazolo[4,3-c]pyridine-3,5- dicarboxylate (6e)

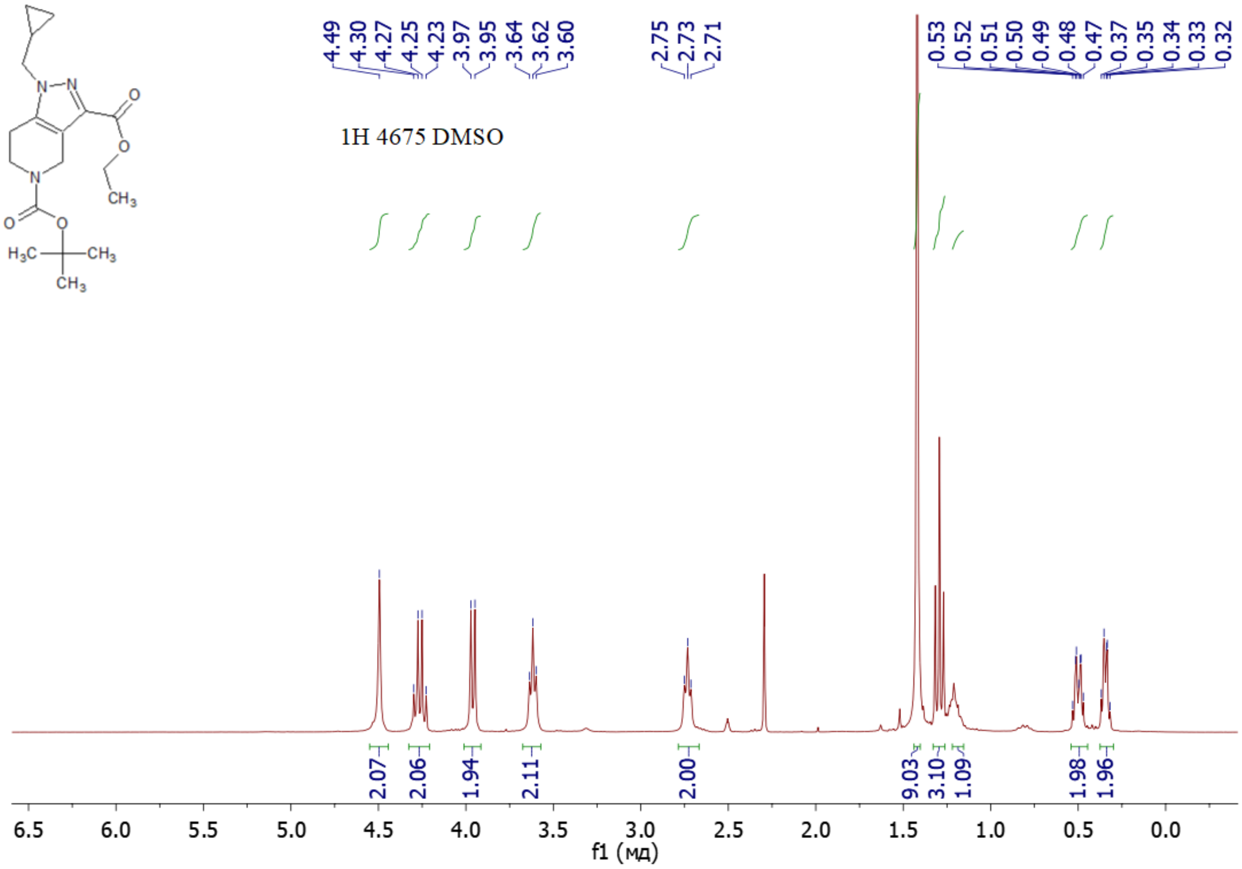

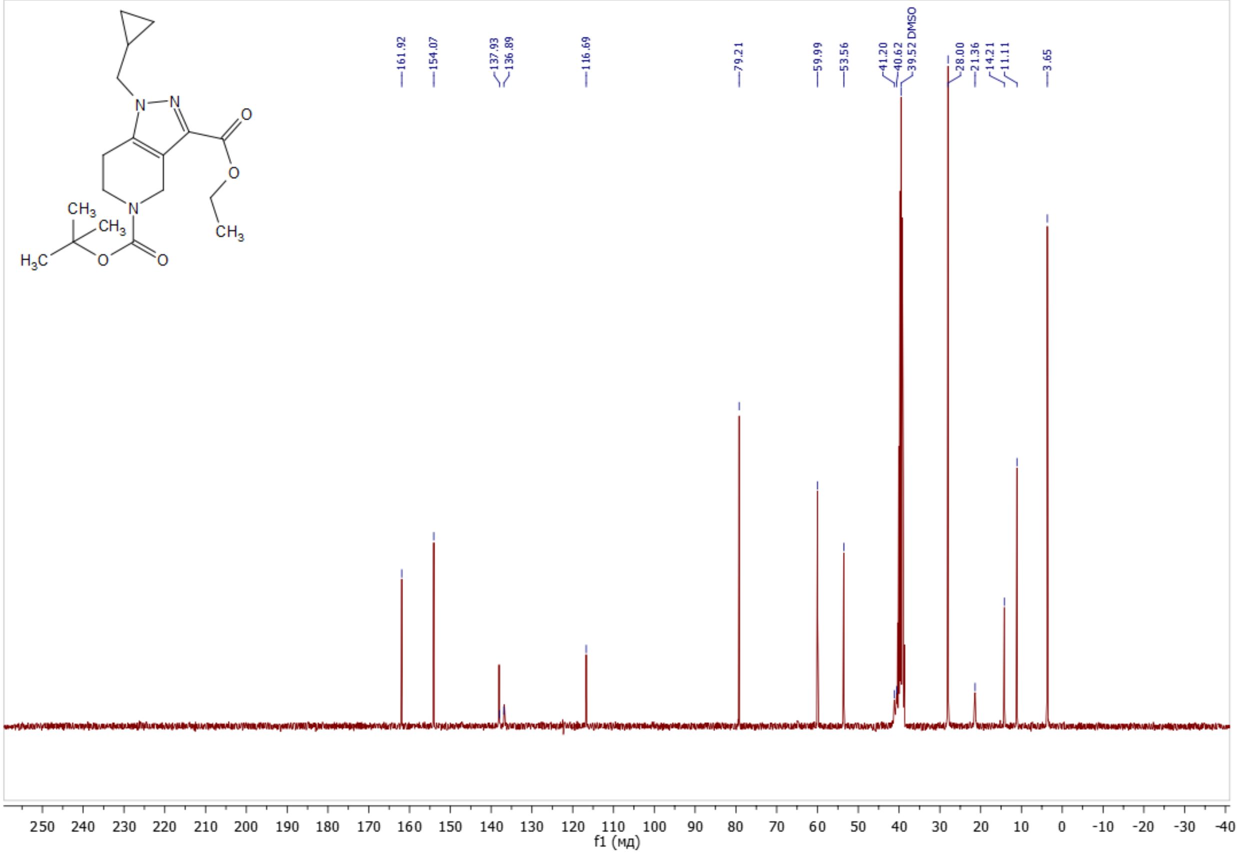

5-tert-butyl-3-ethyl-1- propyl -1,4,6,7-tetrahydro-5H-pyrazolo[4,3-c]pyridine-3,5-dicarboxylate (6f)

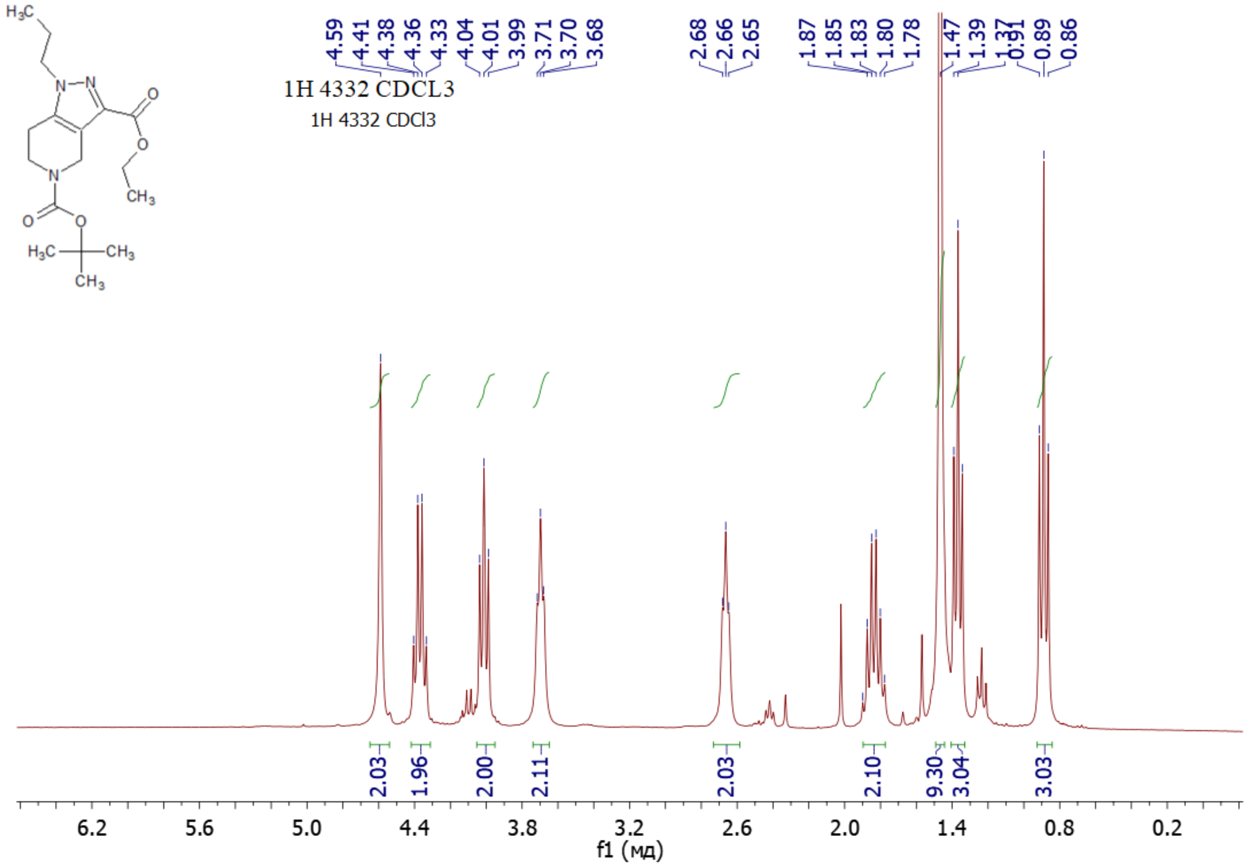

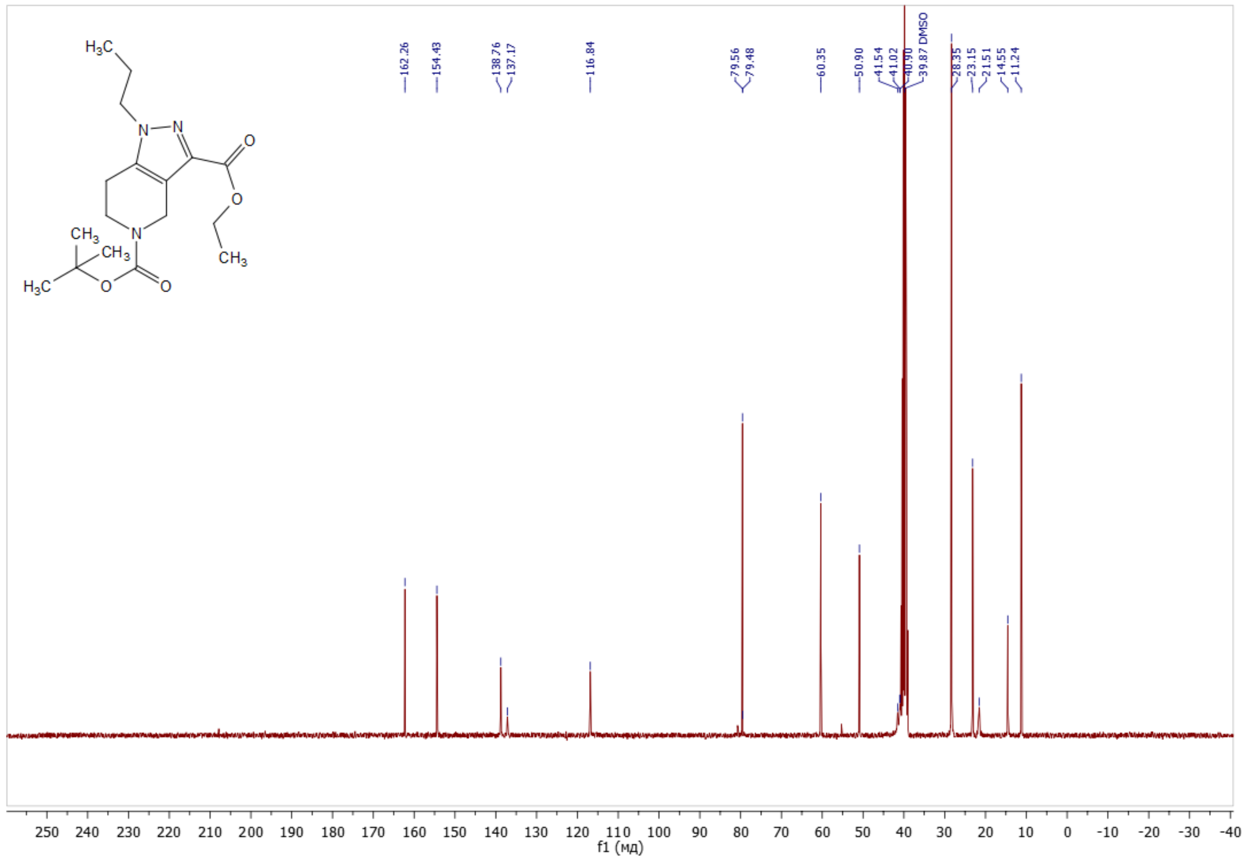

5-tert-butyl-3-ethyl-1-(2-methoxyethyl)-1,4,6,7-tetrahydro-5H-pyrazolo[4,3-c]pyridine-3,5- dicarboxylate (6g)

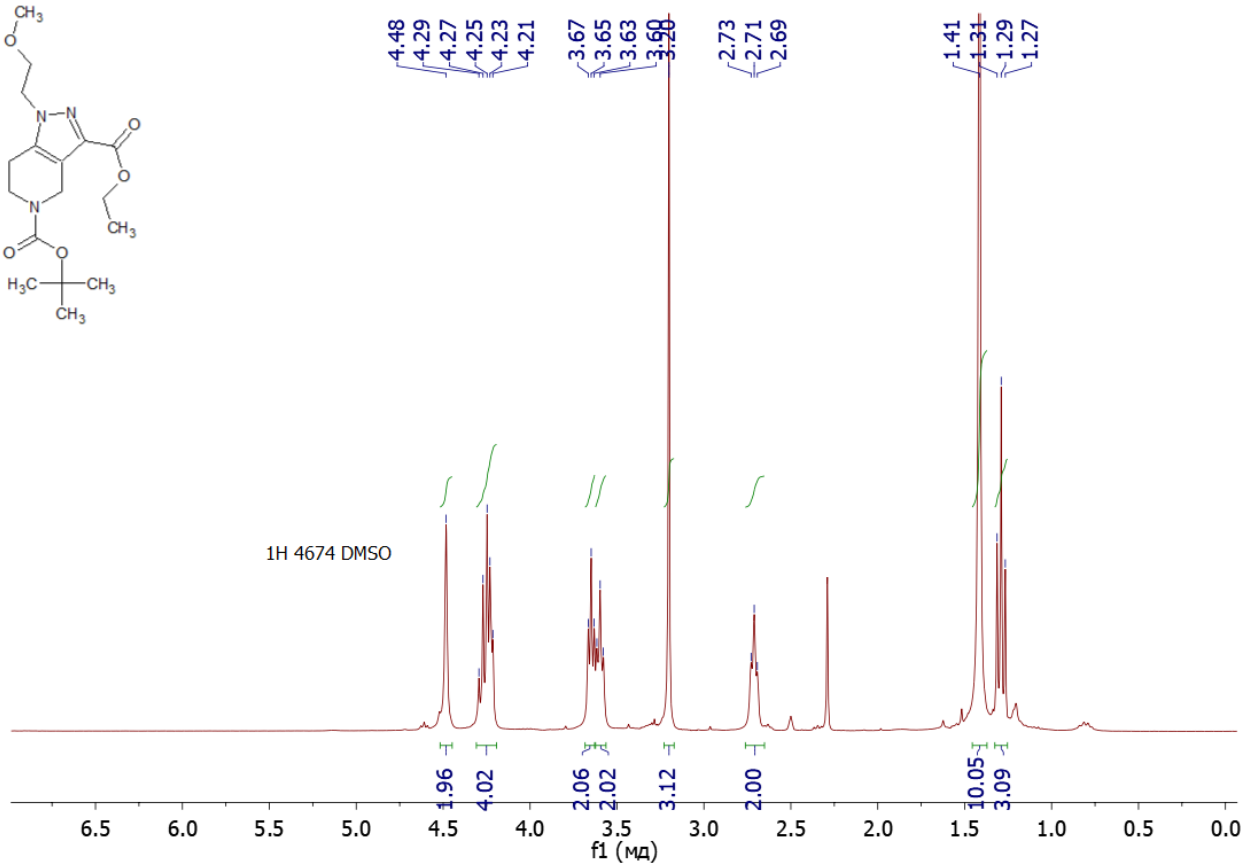

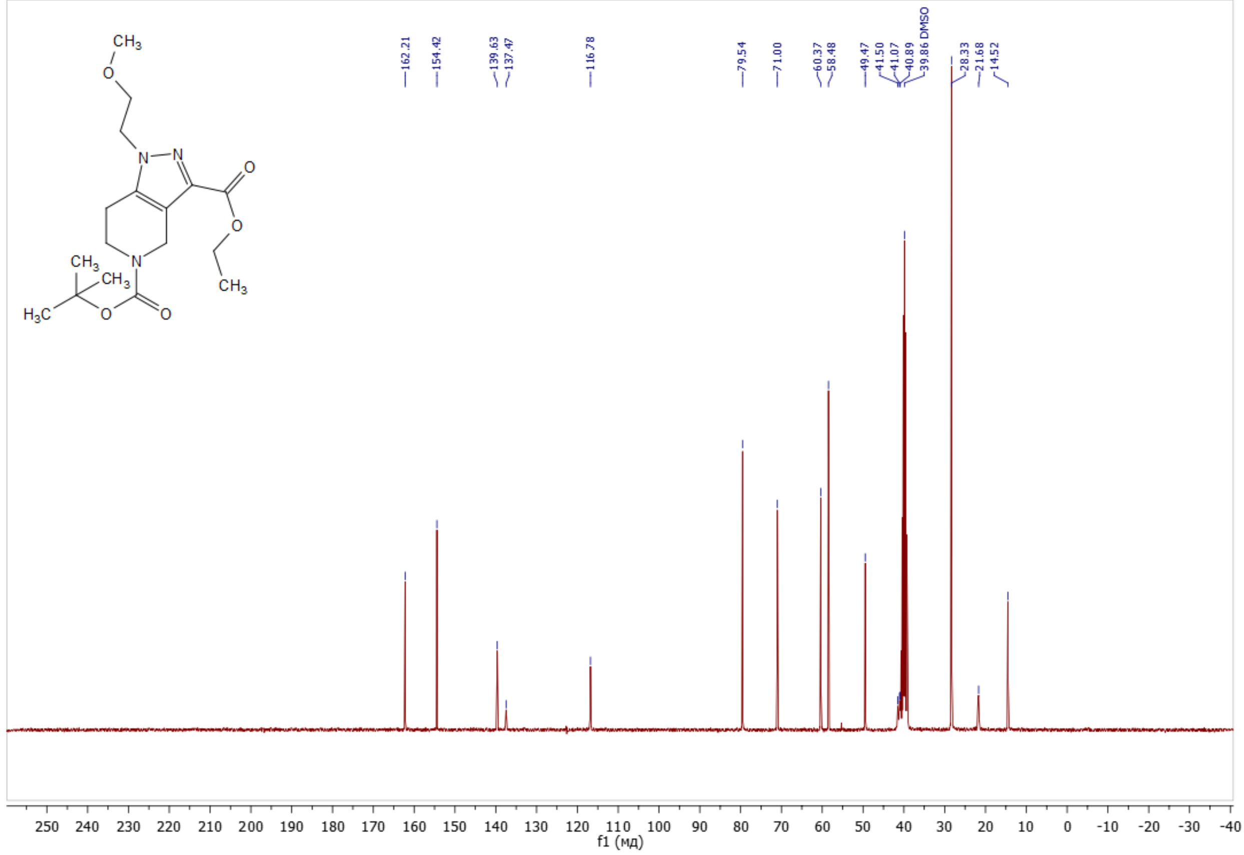

5-tert-butyl-3-ethyl-1-(2-methoxypropyl)-1,4,6,7-tetrahydro-5H-pyrazolo[4,3-c]pyridine-3,5- dicarboxylate (6h)

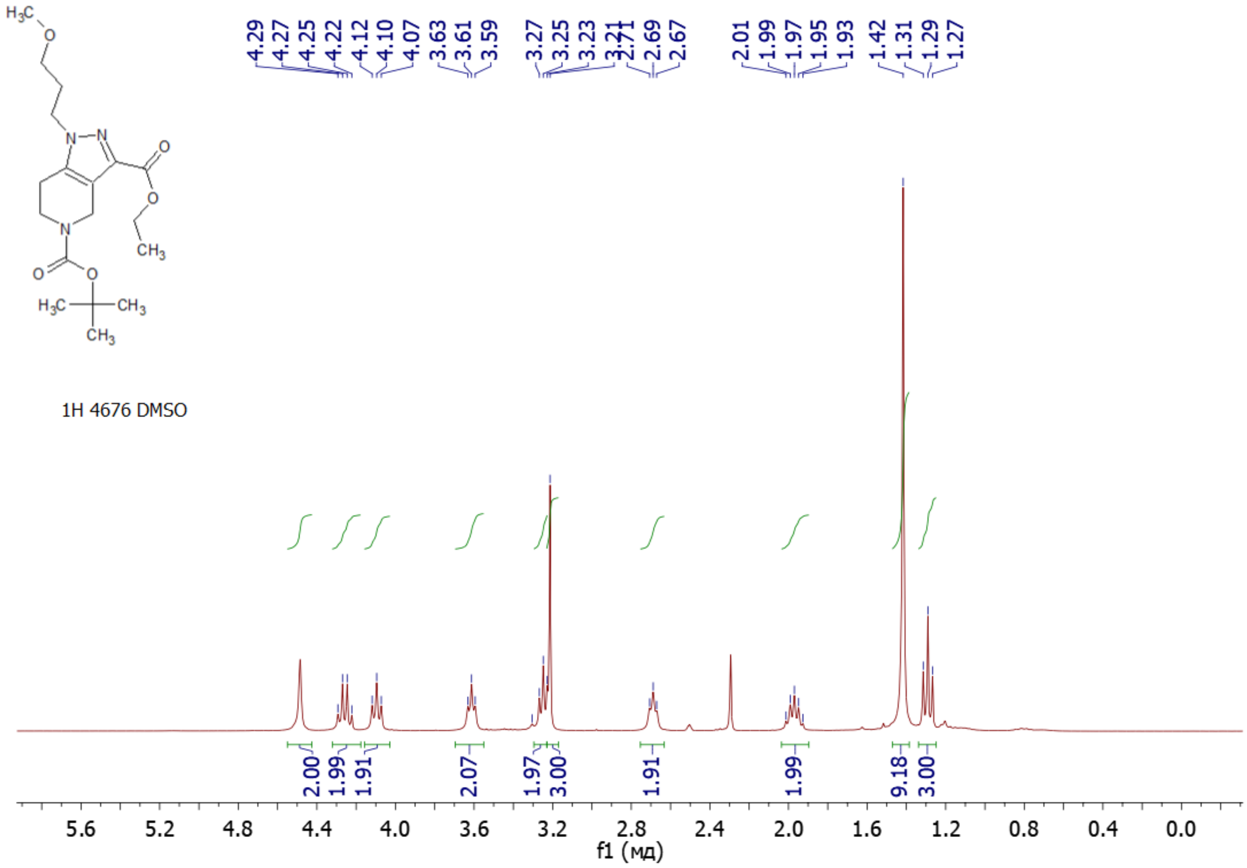

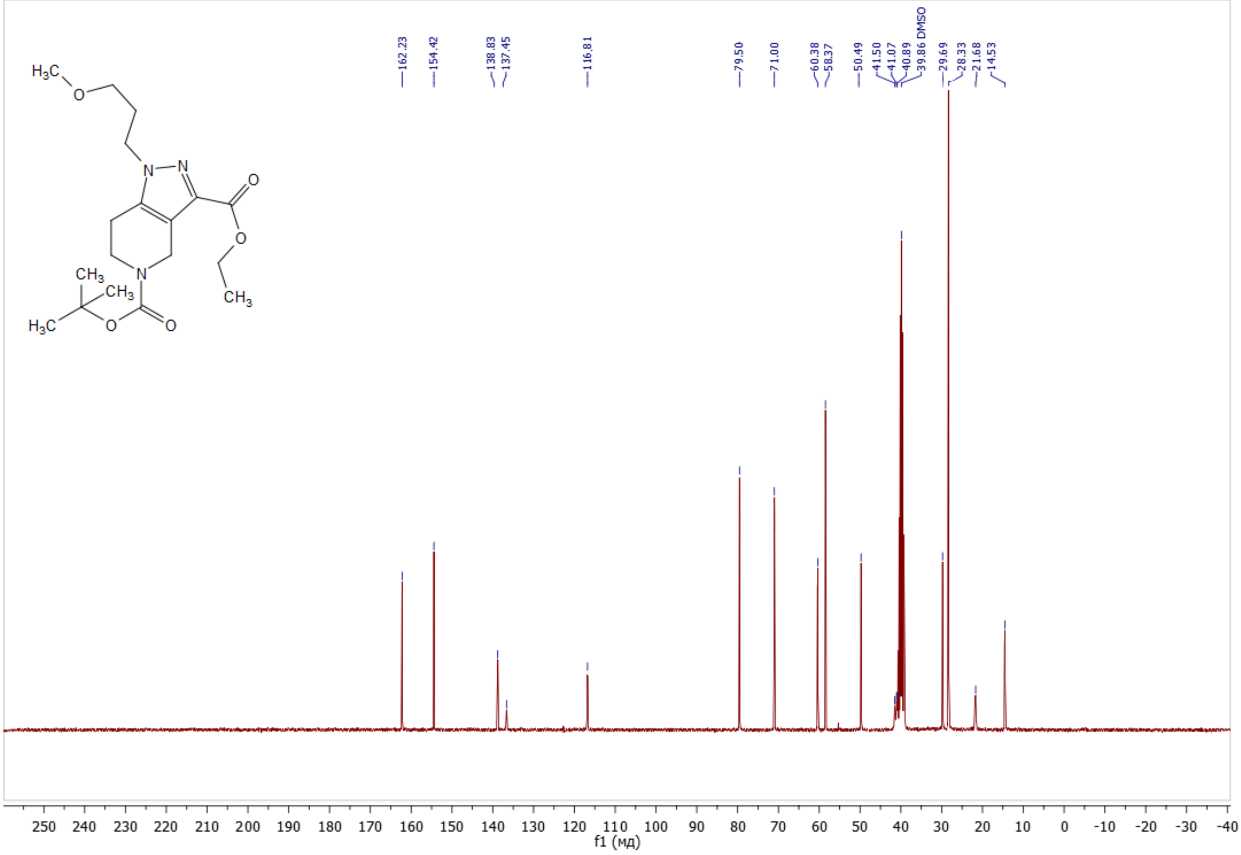

1-methyl-3-(5-methyl-1,3-oxazol-2-yl)-4,5,6,7-tetrahydro-1H-pyrazolo[4,3-c]pyridine hydrochloride (9a)

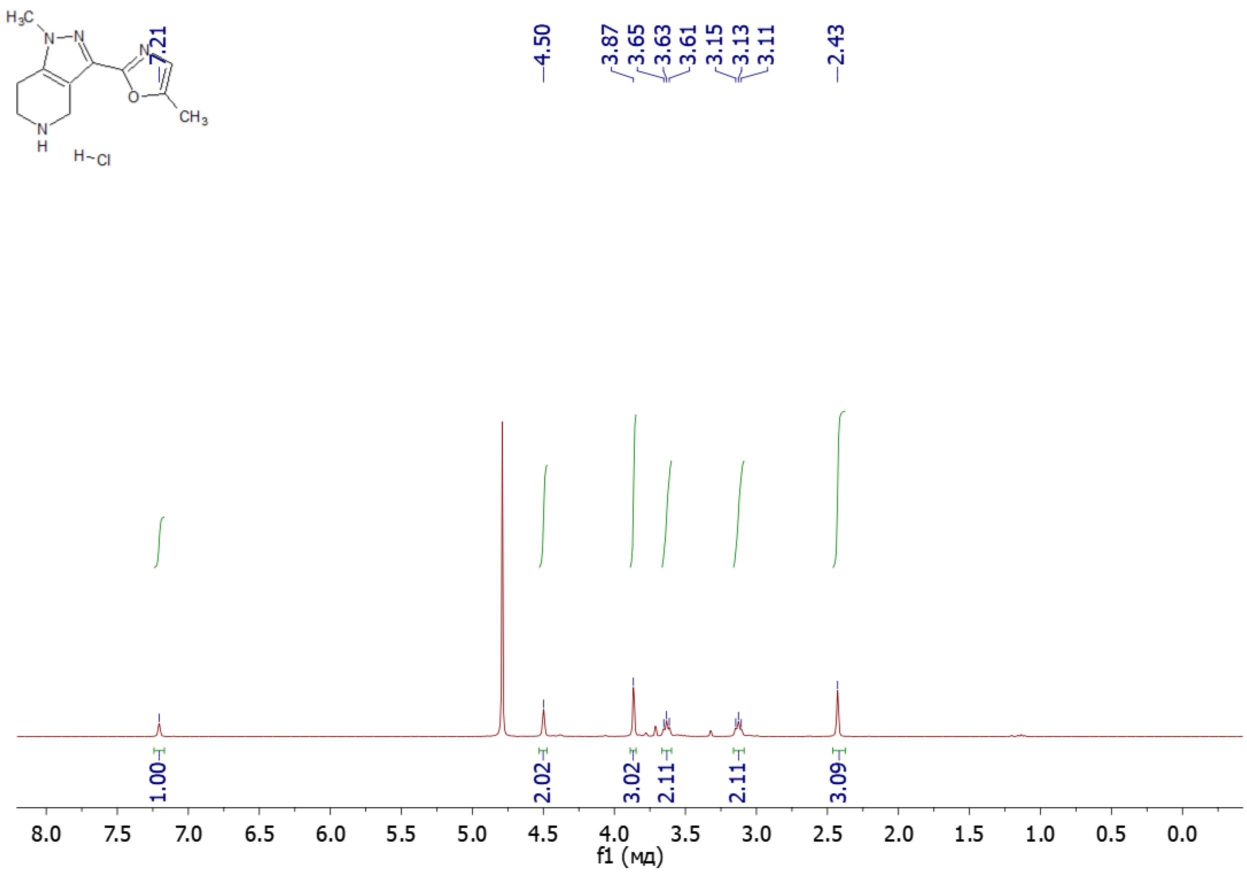

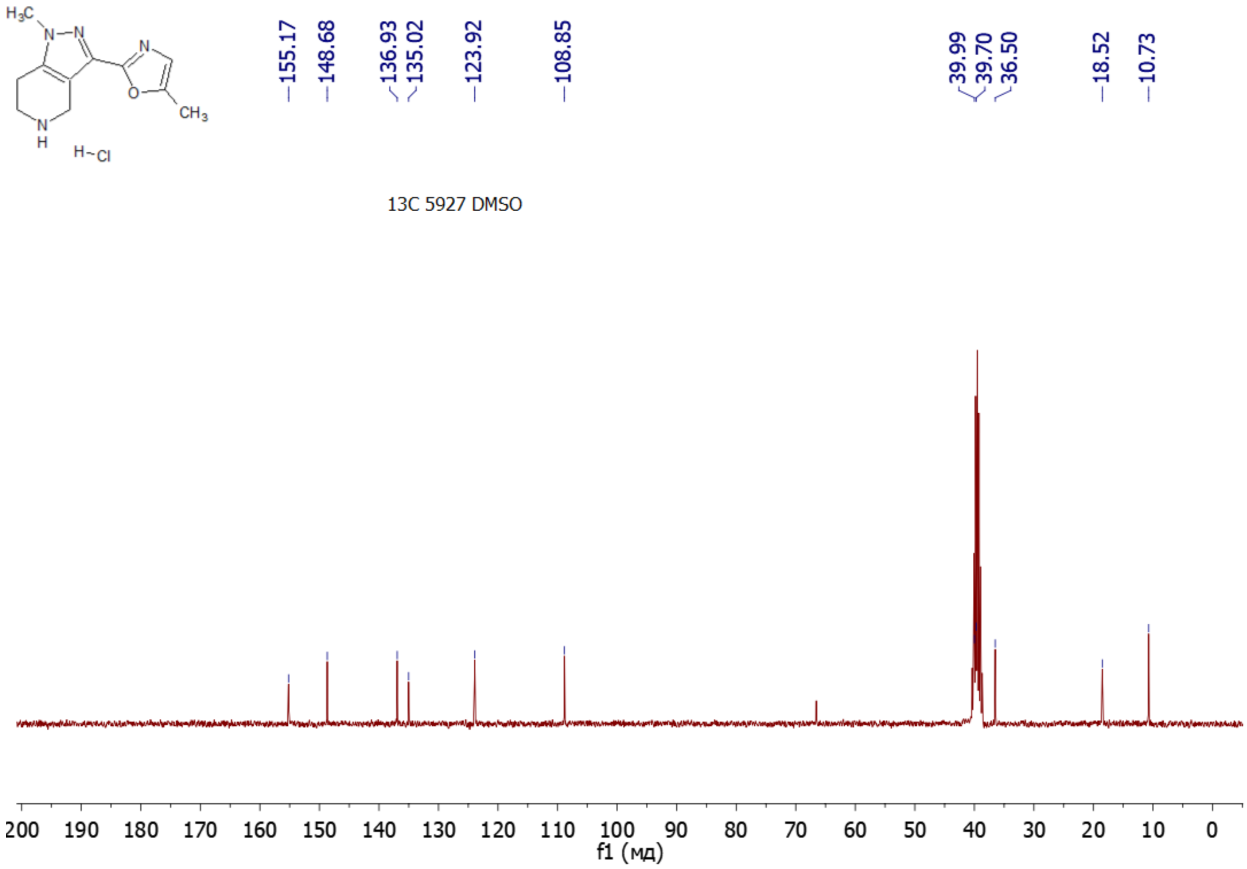

1-ethyl-3-(5-methyl-1,3-oxazol-2-yl)-4,5,6,7-tetrahydro-1H-pyrazolo[4,3-c]pyridine hydrochloride (9b)

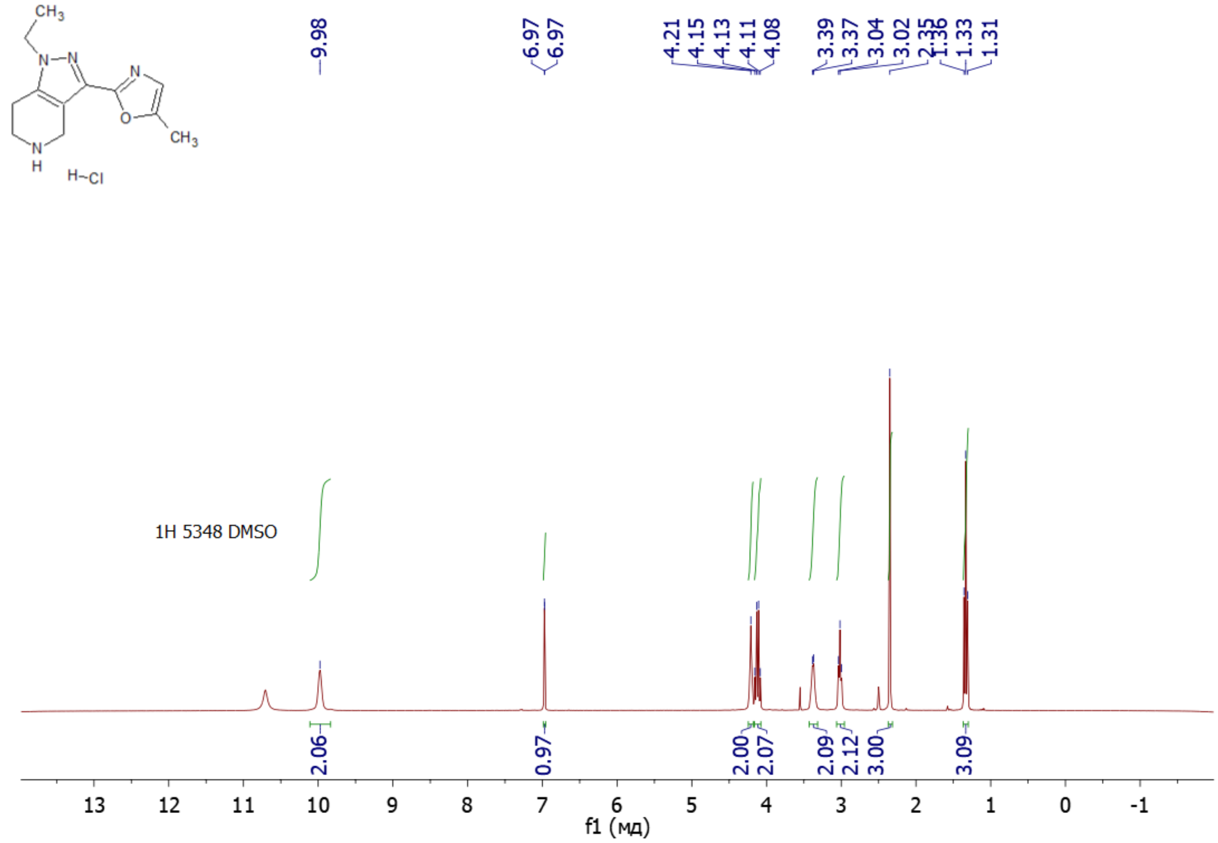

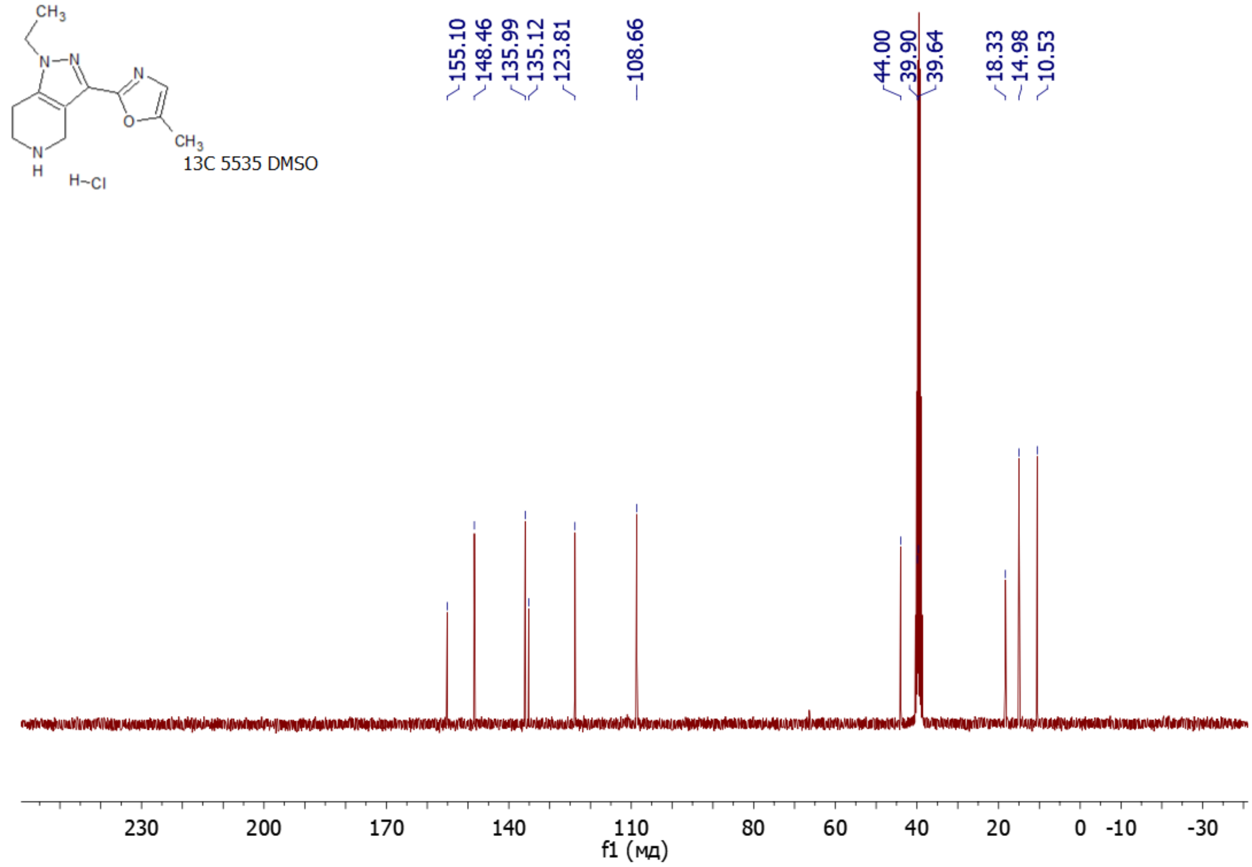

3-(5-methyl-1,3-oxazol-2-yl)-1-(propan-2-yl)-4,5,6,7-tetrahydro-1H-pyrazolo[4,3-c]pyridine hydrochloride (9c)

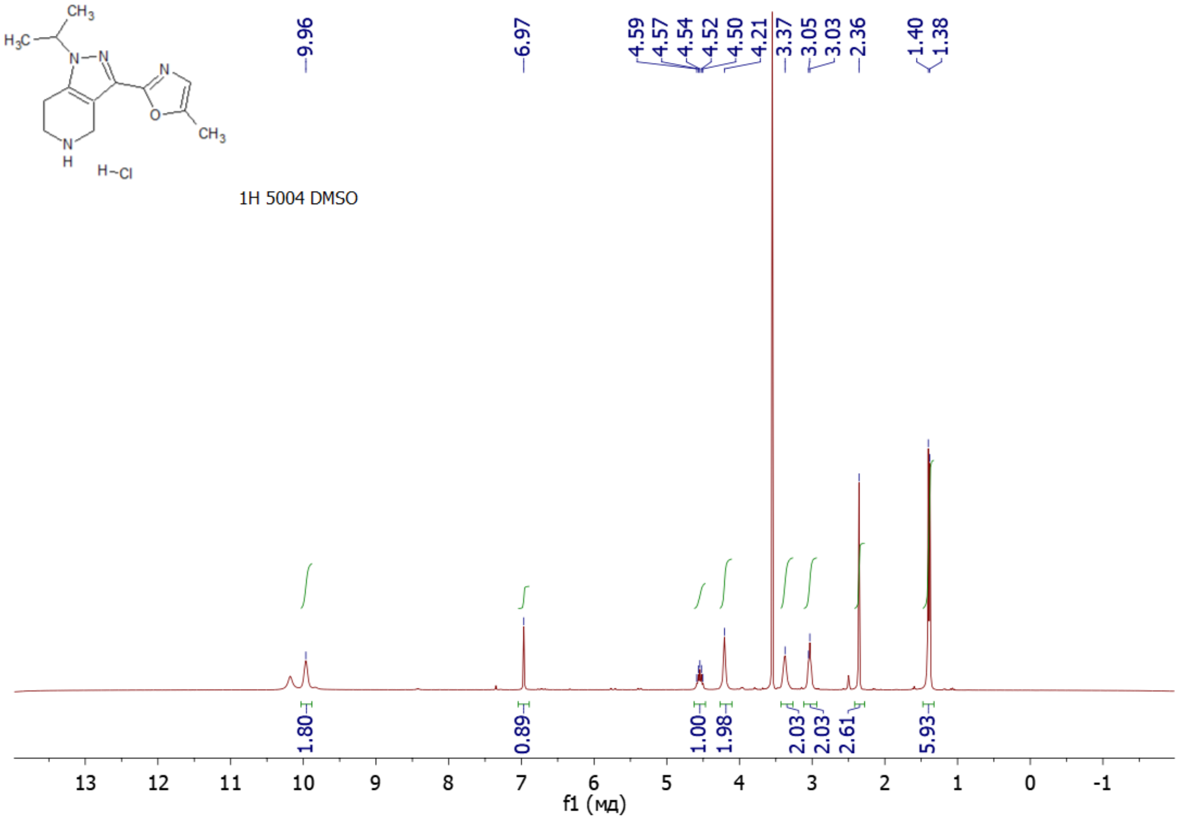

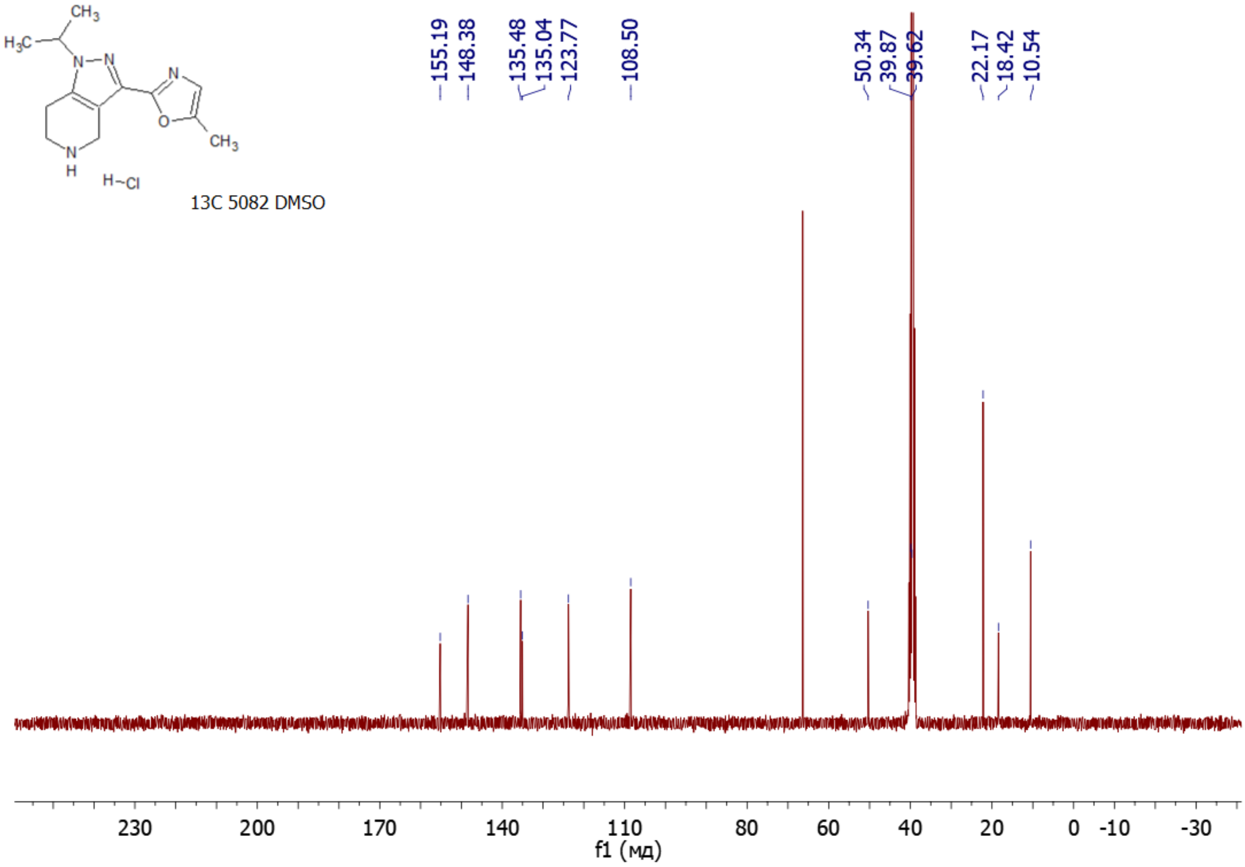

3-(5-methyl-1,3-oxazol-2-yl)-1-(2-methylpropyl)-4,5,6,7-tetrahydro-1H-pyrazolo[4,3-c]pyridine hydrochloride (9d)

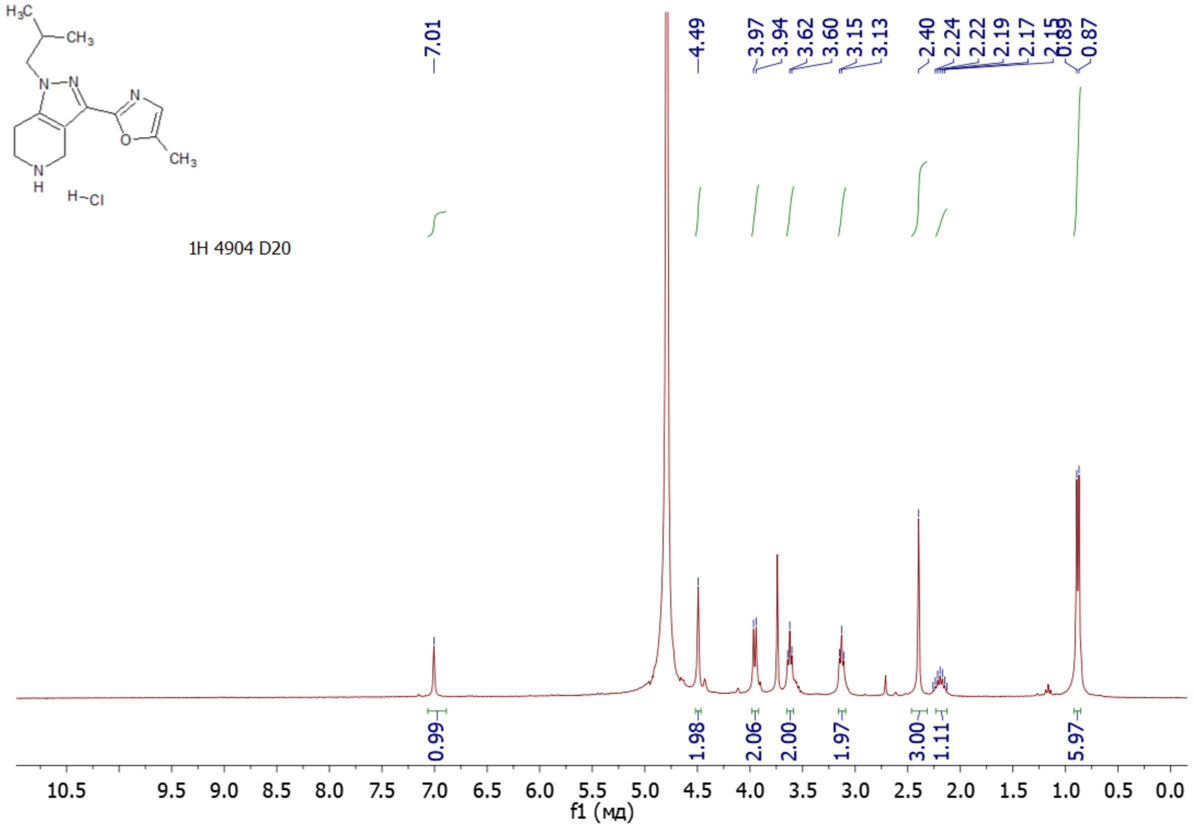

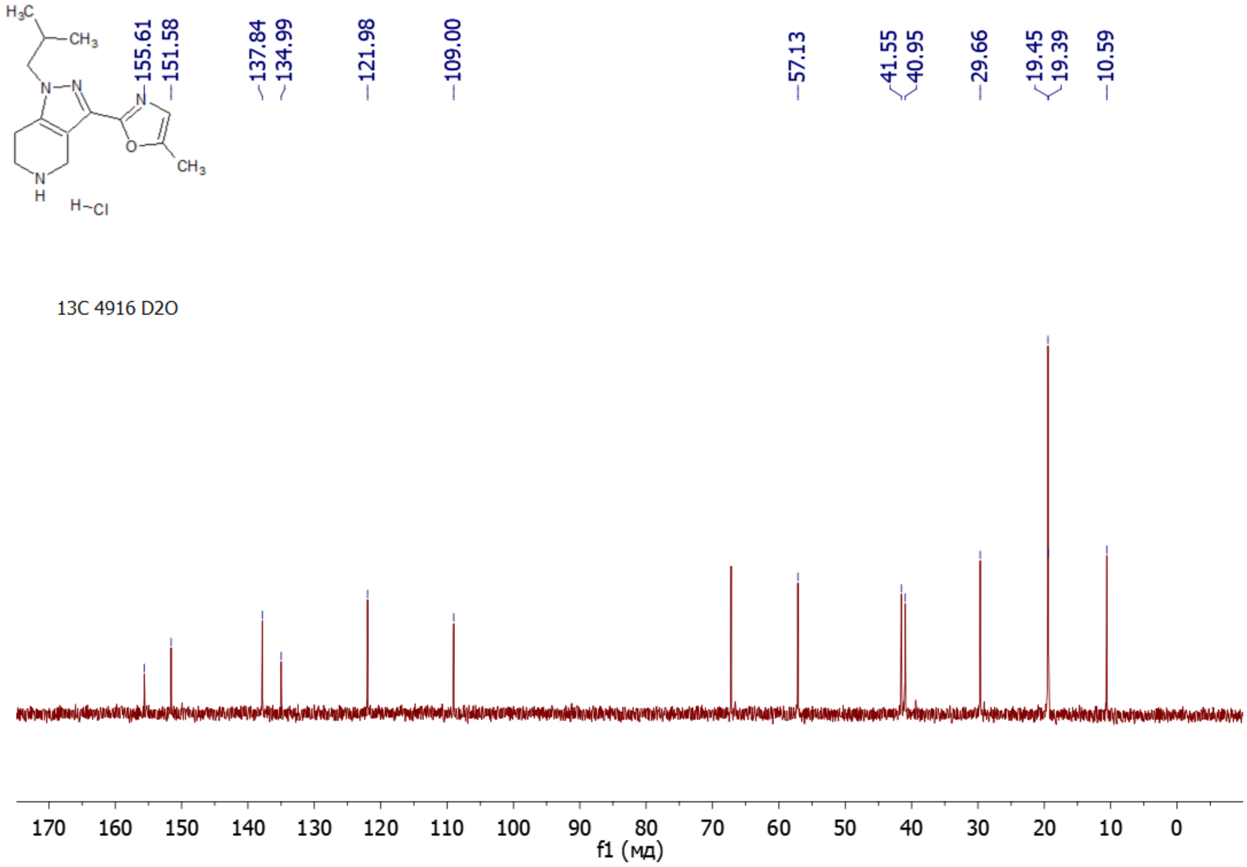

tert-butyl 1-(cyclopropylmethyl)-3-formyl-1,4,6,7-tetrahydro-5H-pyrazolo[4,3-c]pyridine-5- carboxylate (11e)

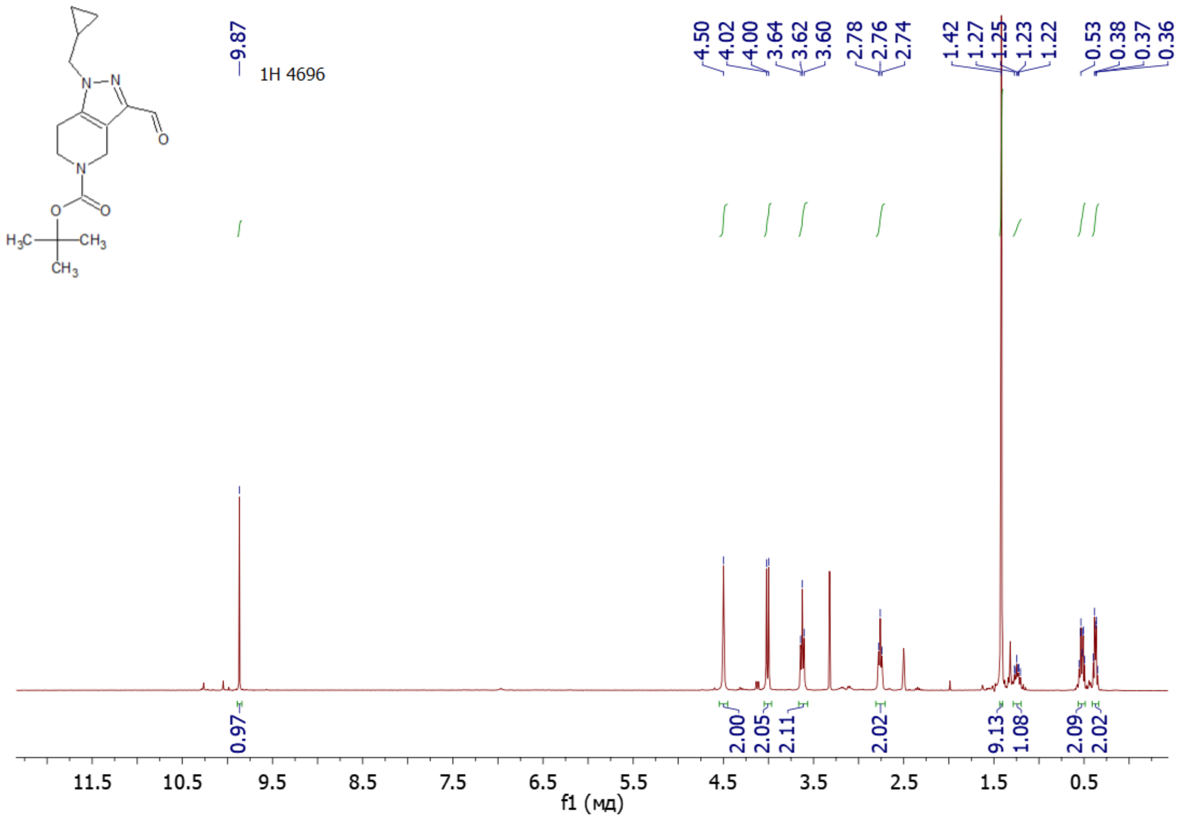

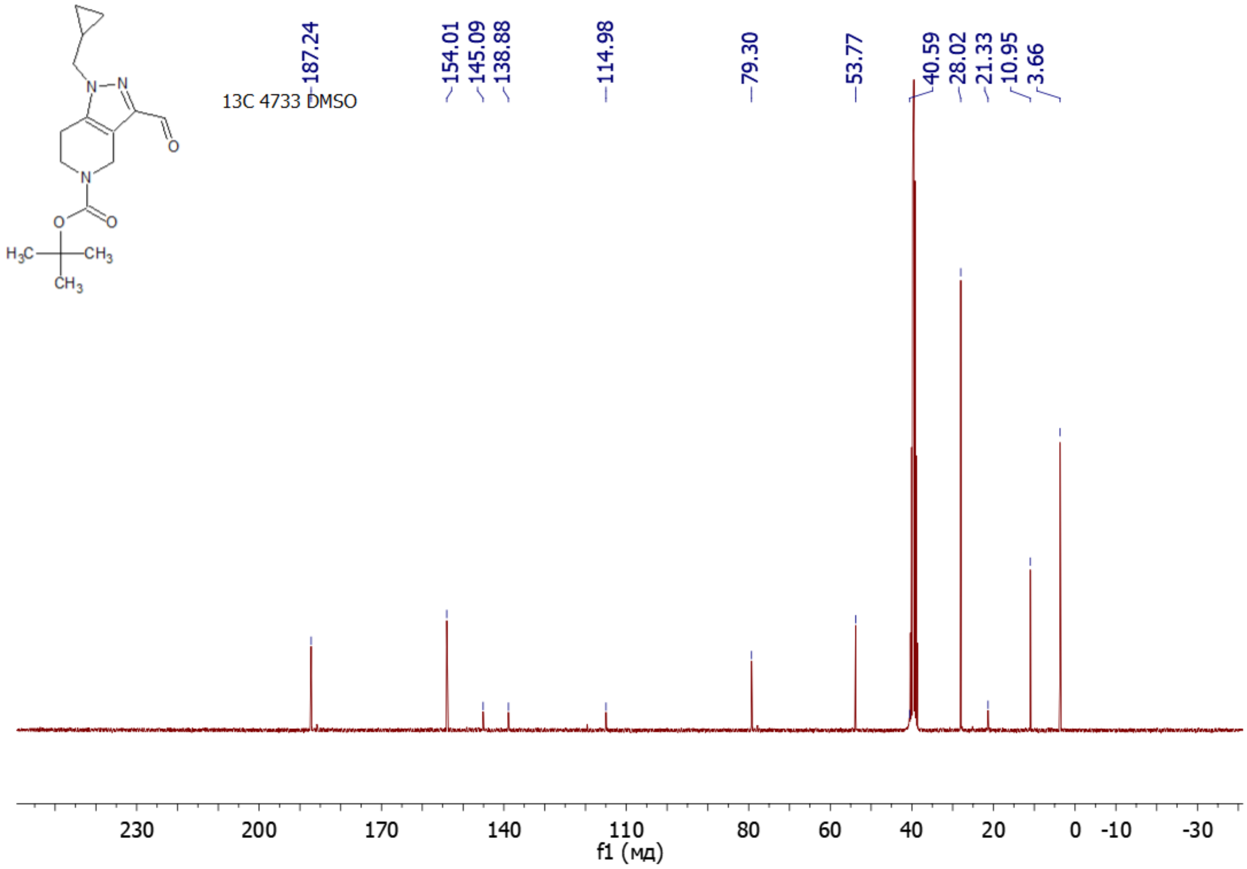

tert-butyl 3-formyl-1-propyl-1,4,6,7-tetrahydro-5H-pyrazolo[4,3-c]pyridine-5-carboxylate (11f)

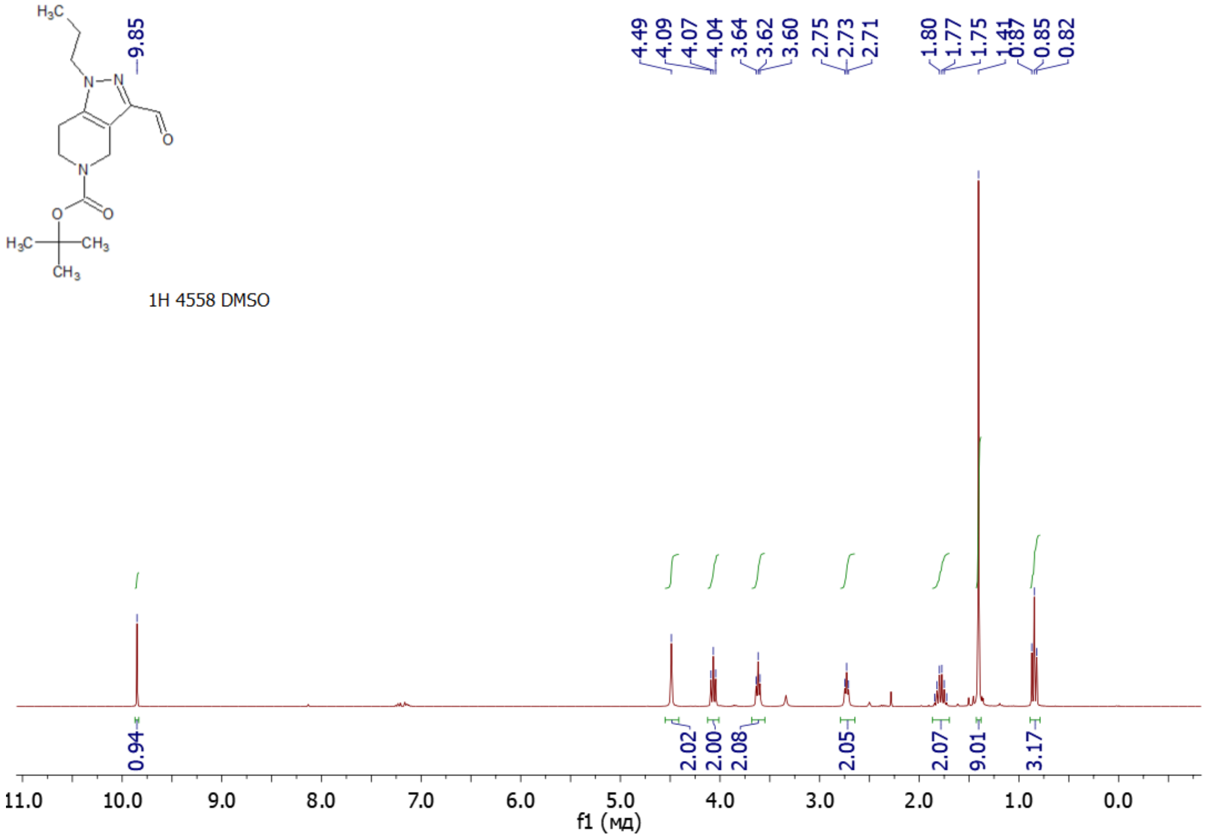

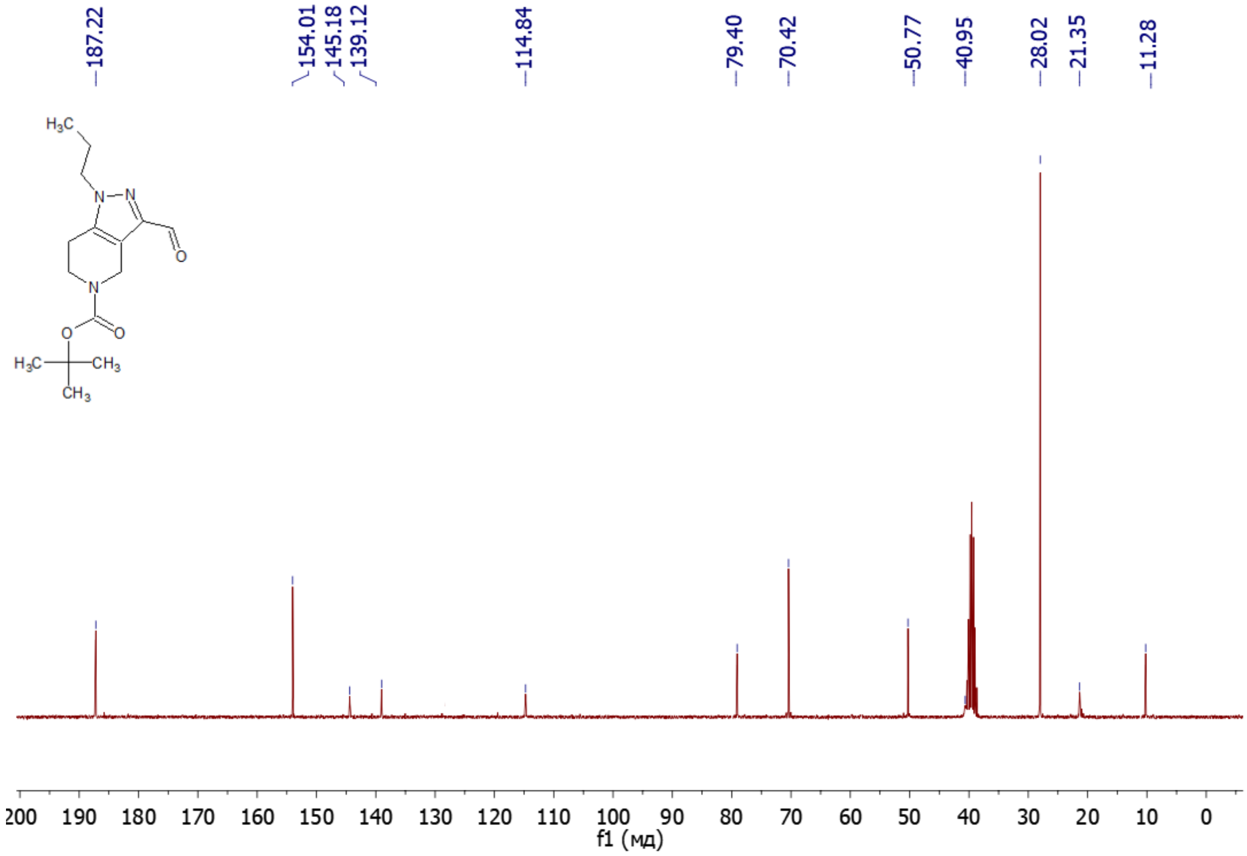

tert-butyl 3-formyl-1-(2-methoxyethyl)-1,4,6,7-tetrahydro-5H-pyrazolo[4,3-c]pyridine-5-carboxylate (11g)

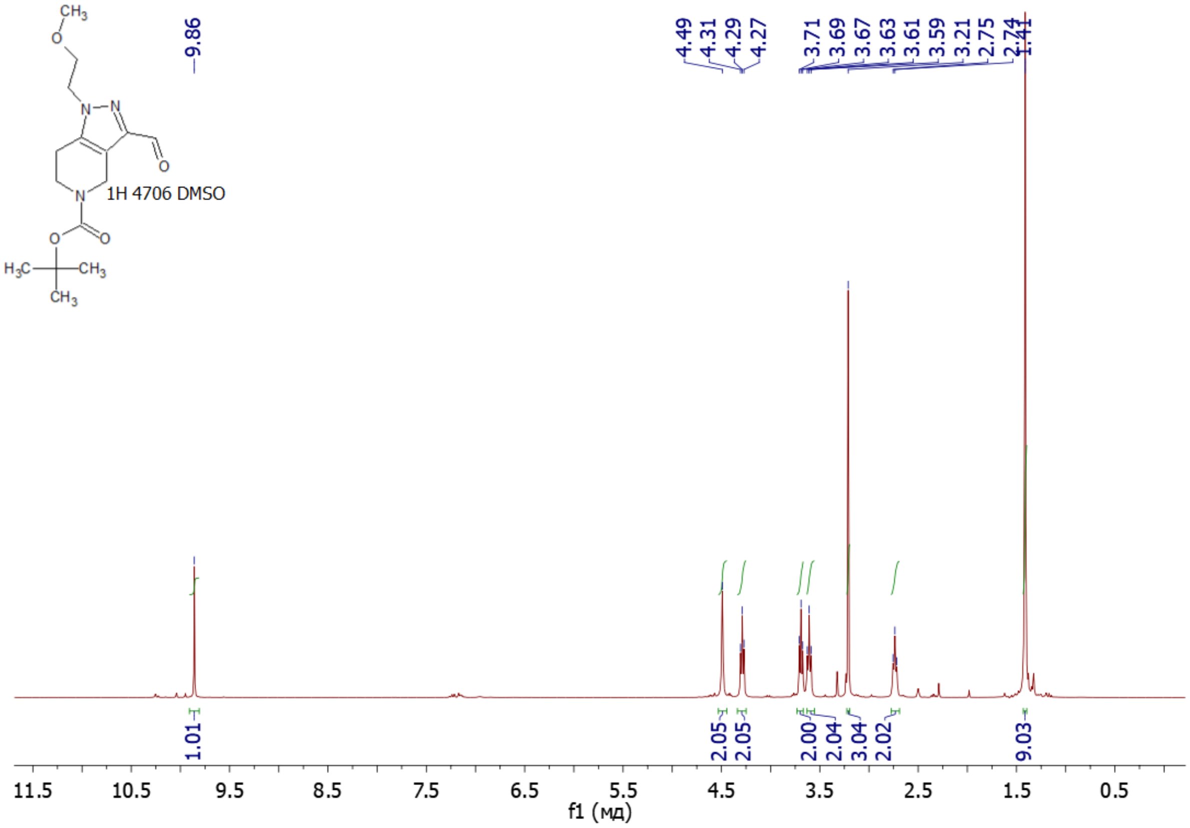

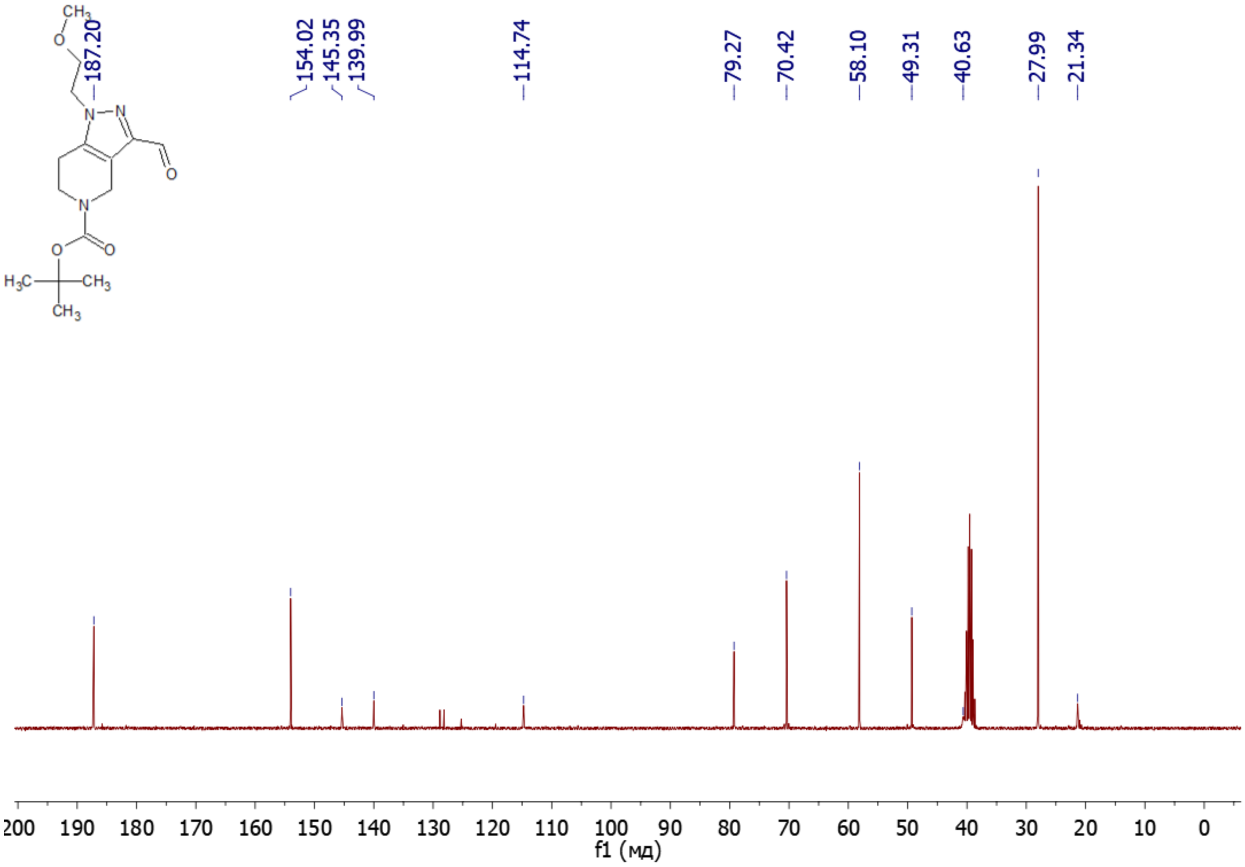

tert-butyl 3-formyl-1-(3-methoxypropyl)-1,4,6,7-tetrahydro-5H-pyrazolo[4,3-c]pyridine-5-carboxylate (11h)

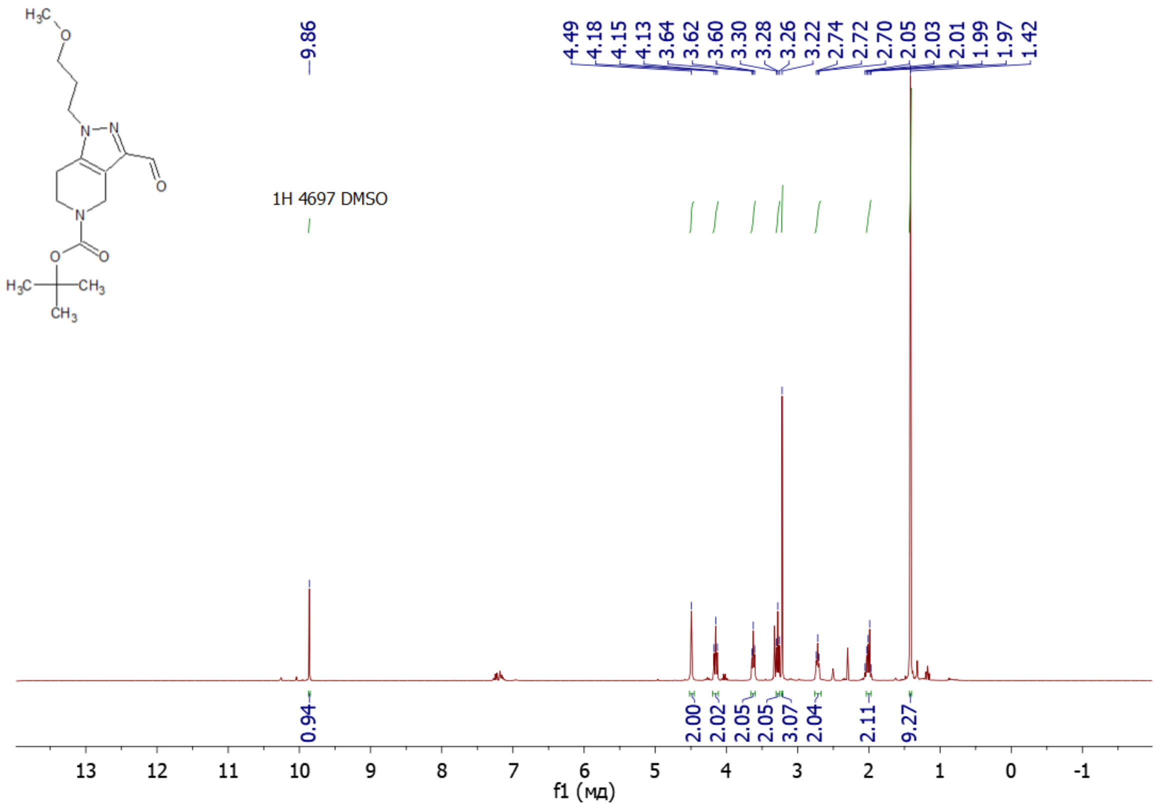

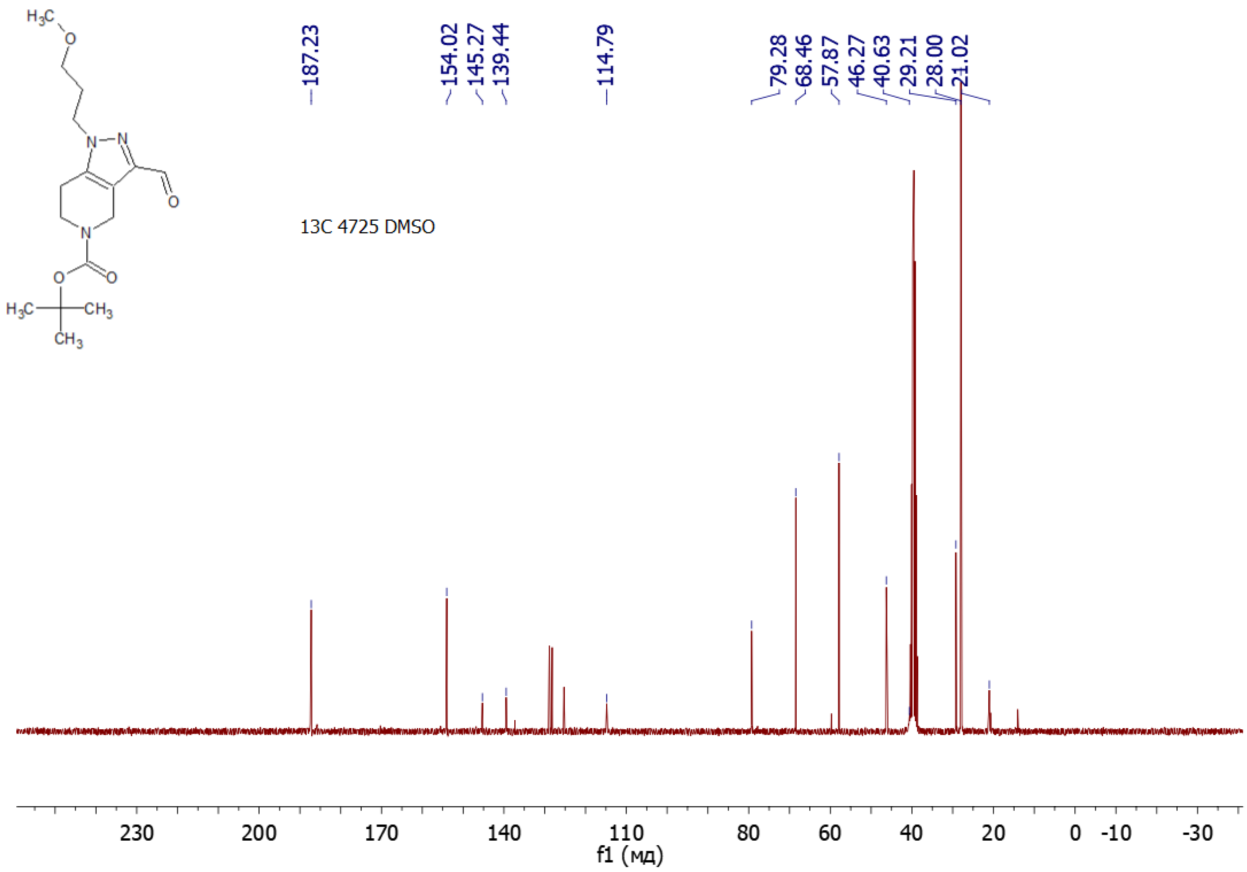

1-(cyclopropylmethyl)-3-(1,3-oxazol-5-yl)-4,5,6,7-tetrahydro-1H-pyrazolo[4,3-c]pyridine hydrochloride (12e)

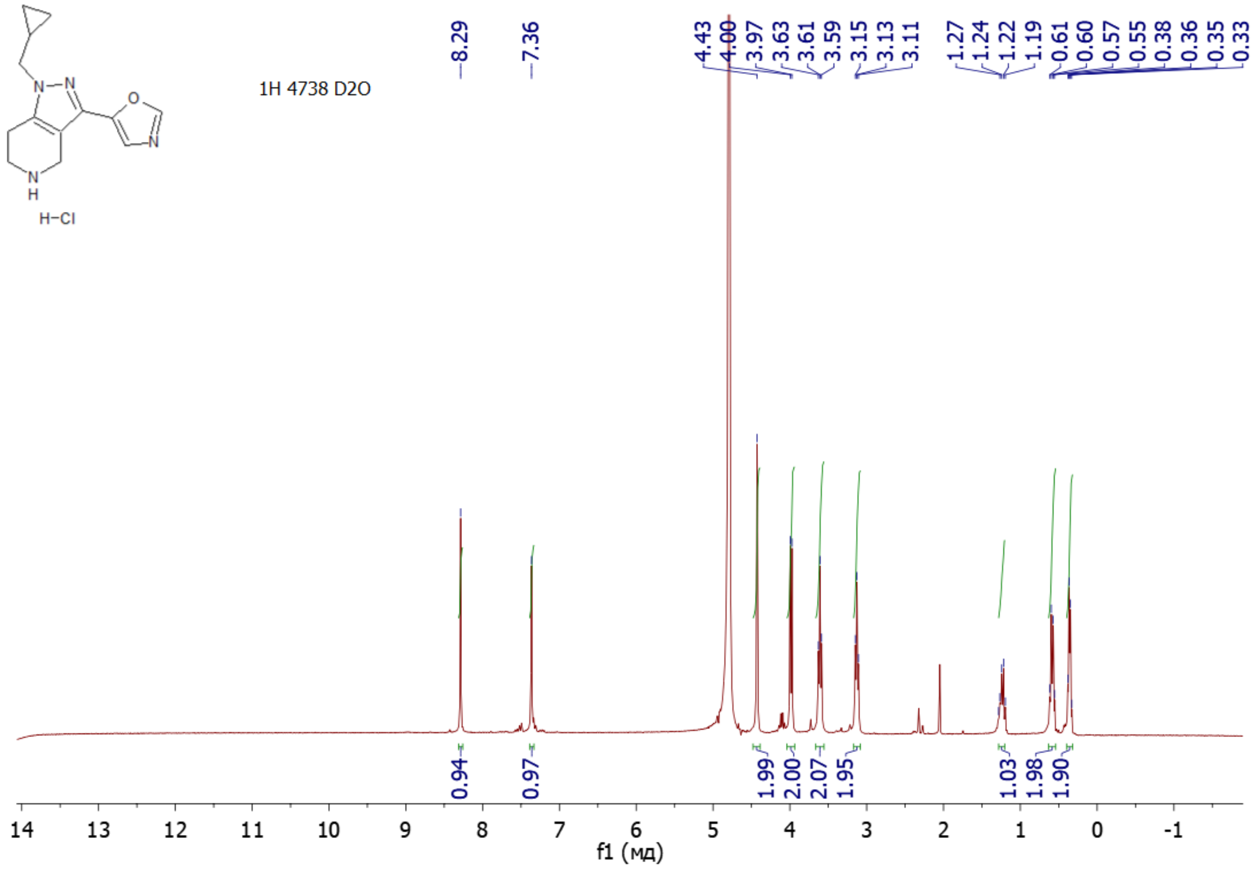

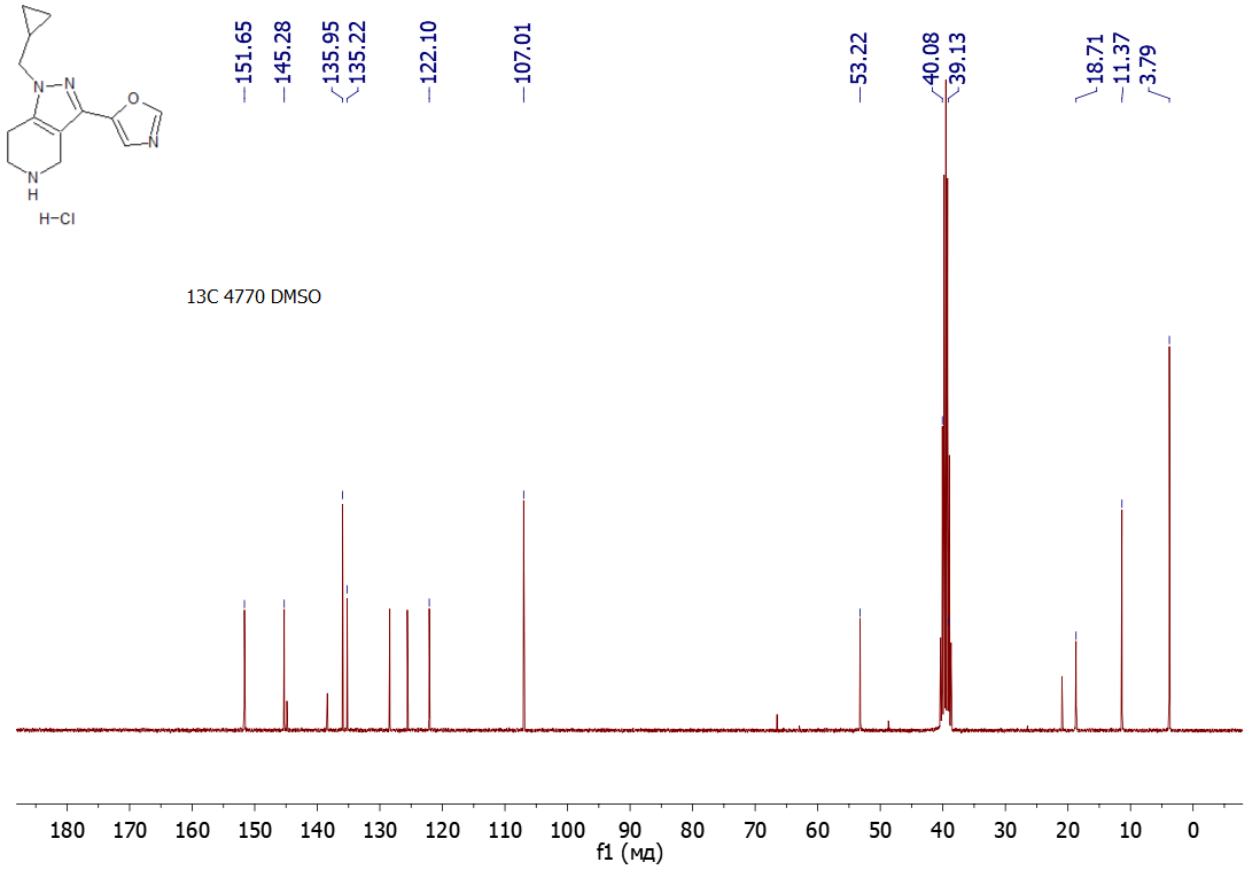

3-(1,3-oxazol-5-yl)-1-propyl-4,5,6,7-tetrahydro-1H-pyrazolo[4,3-c]pyridine hydrochloride (12f)

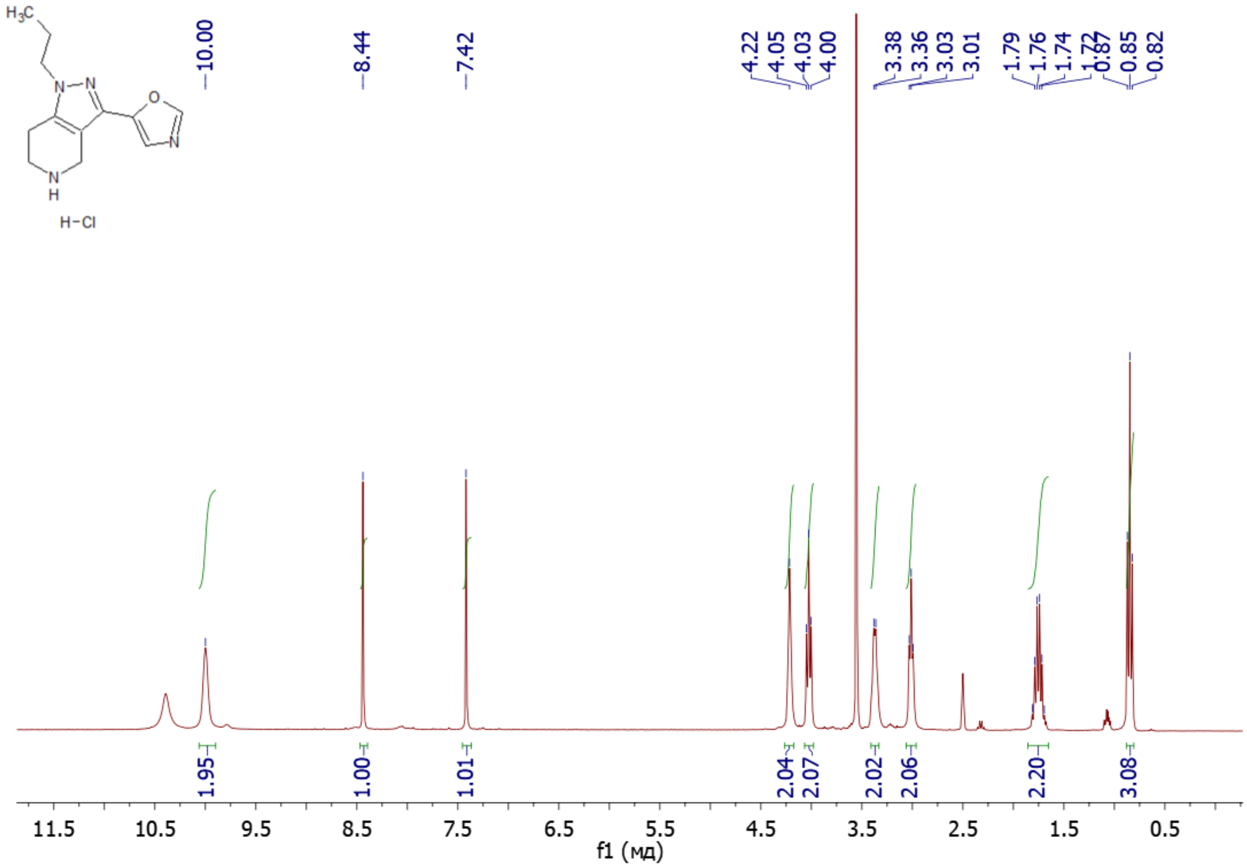

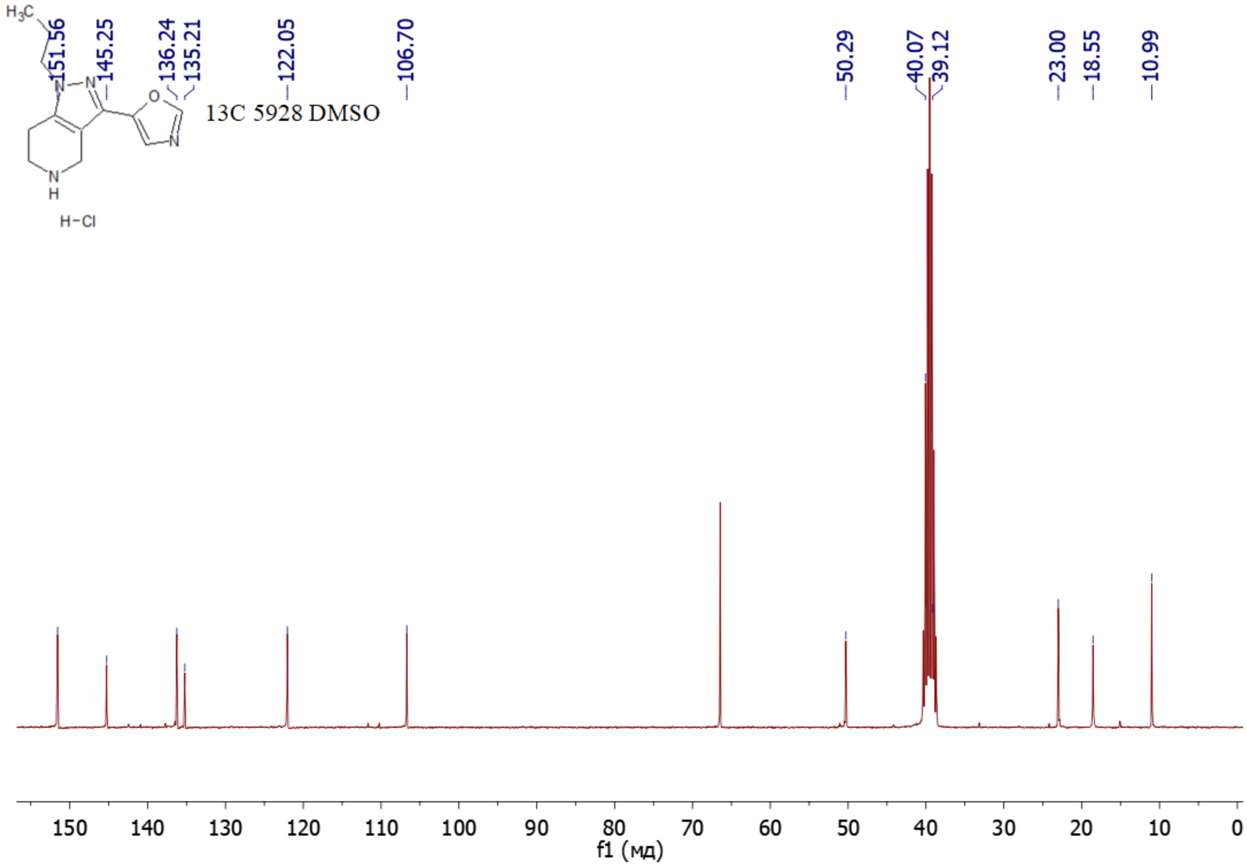

1-(2-methoxyethyl)-3-(1,3-oxazol-5-yl)-4,5,6,7-tetrahydro-1H-pyrazolo[4,3-c]pyridine hydrochloride (12g)

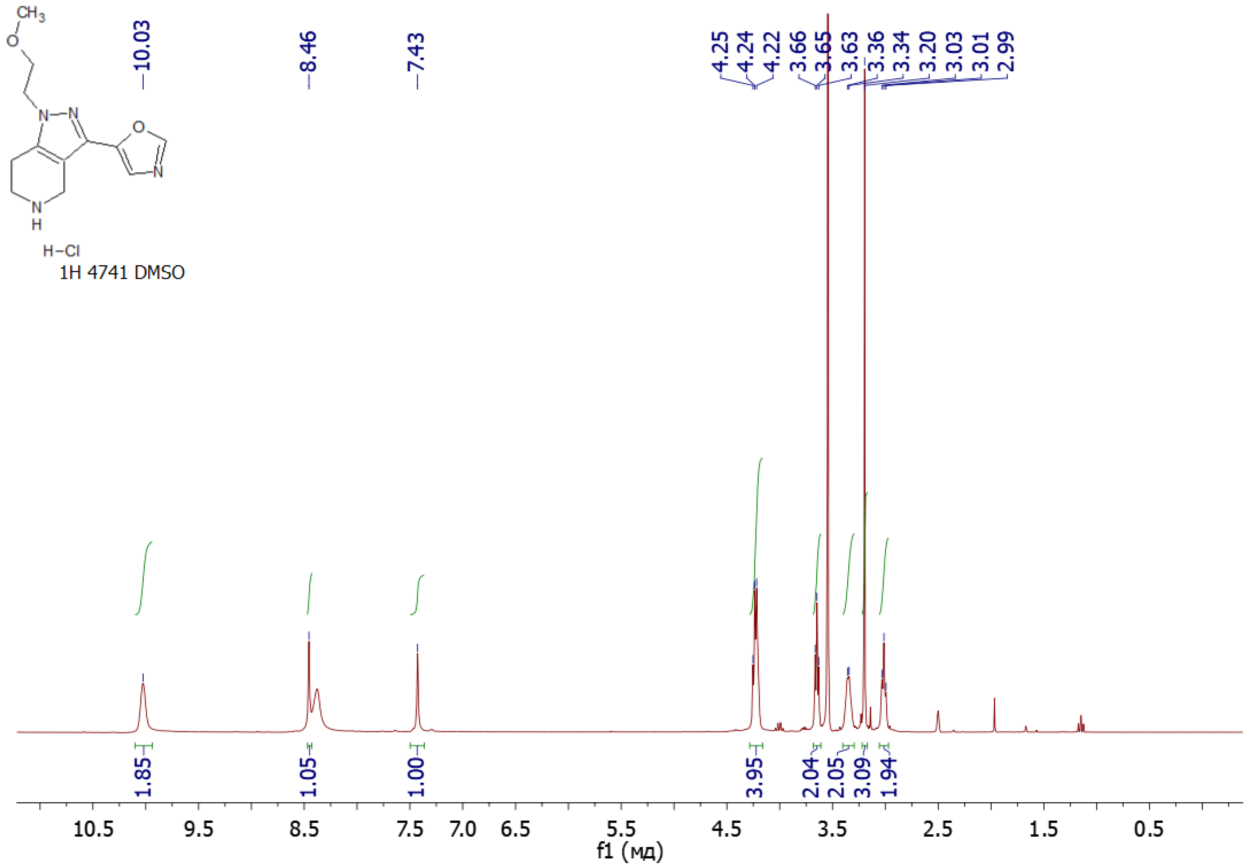

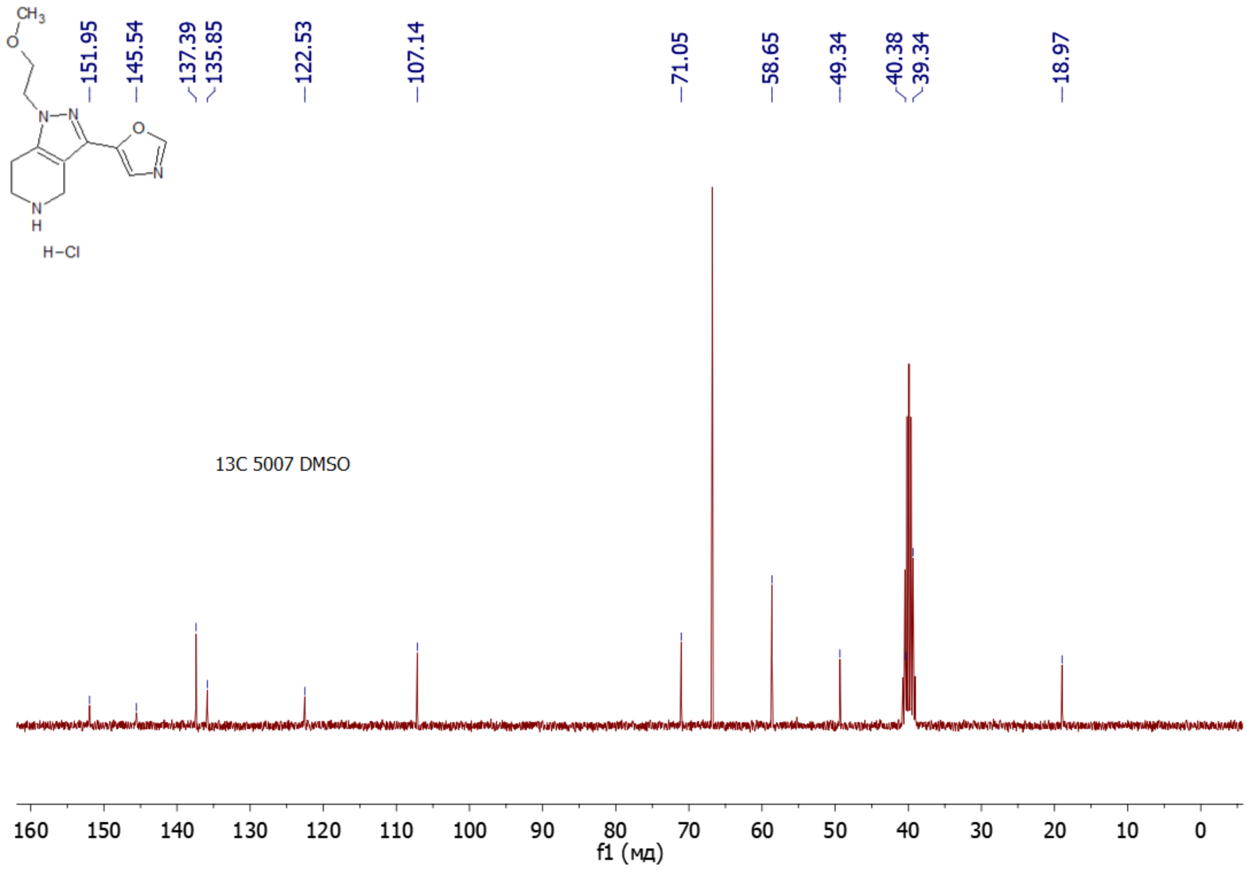

1-(3-methoxypropyl)-3-(1,3-oxazol-5-yl)-4,5,6,7-tetrahydro-1H-pyrazolo[4,3-c]pyridine hydrochloride (12h)

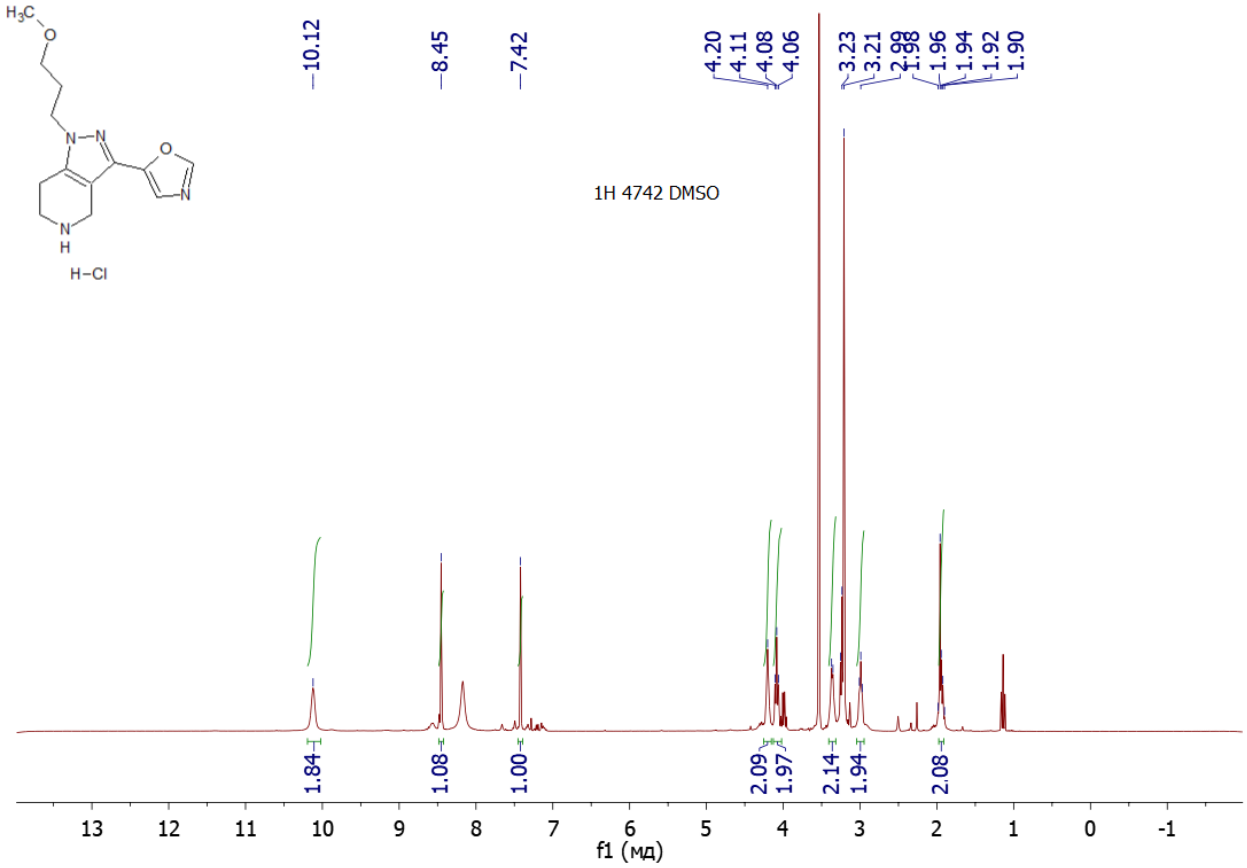

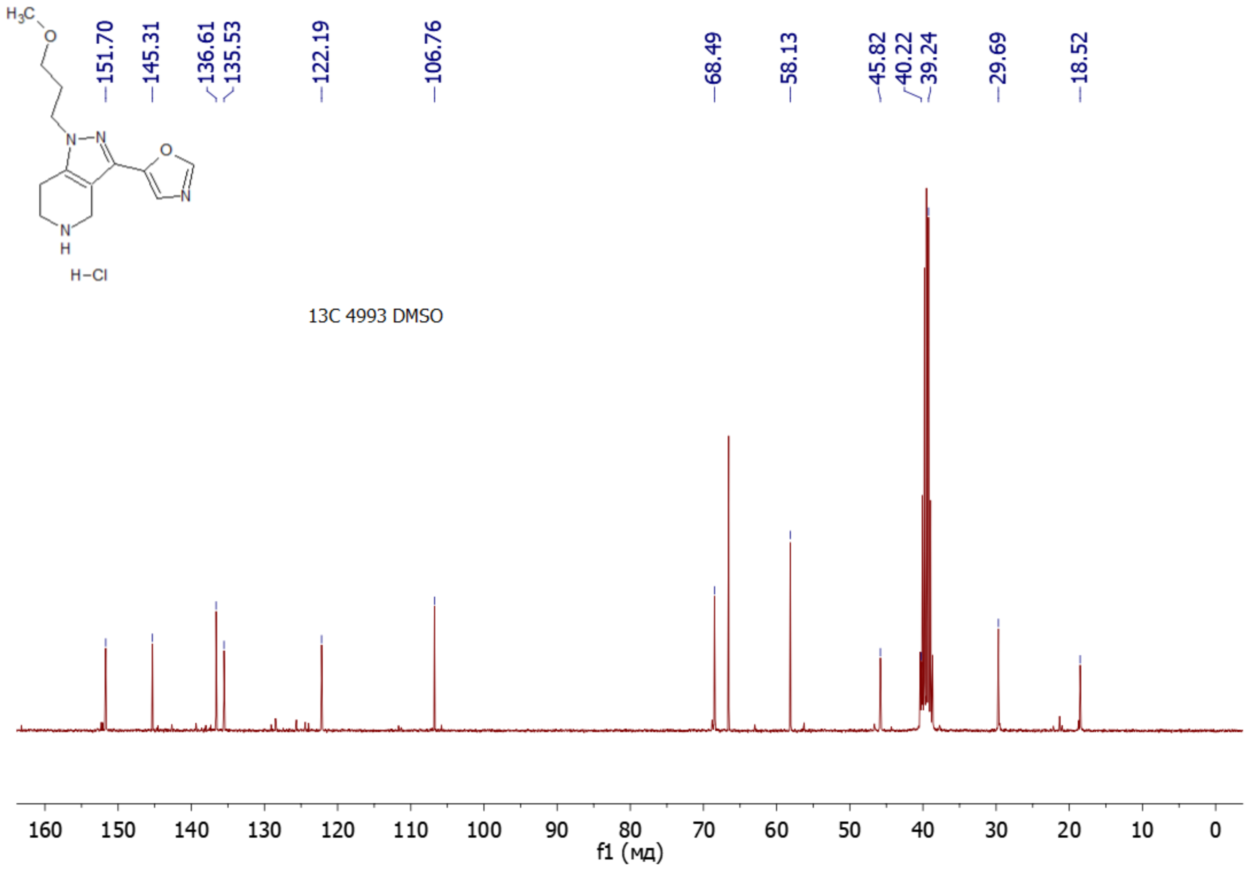

1-methyl-3-(5-methyl-1,3-oxazol-2-yl)-5-(5-nitro-2-furoyl)-4,5,6,7-tetrahydro-1H-pyrazolo [4,3-c]pyridine, LK01510 (10a)

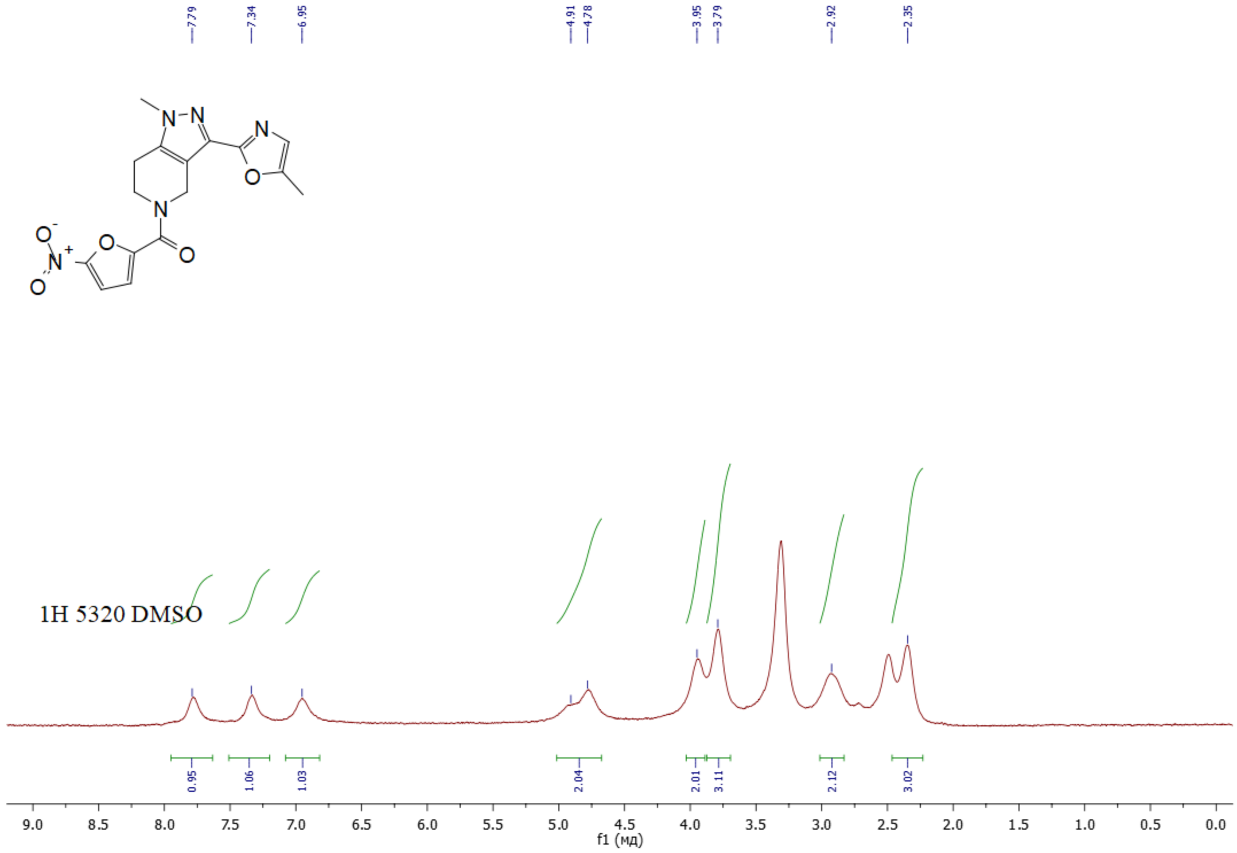

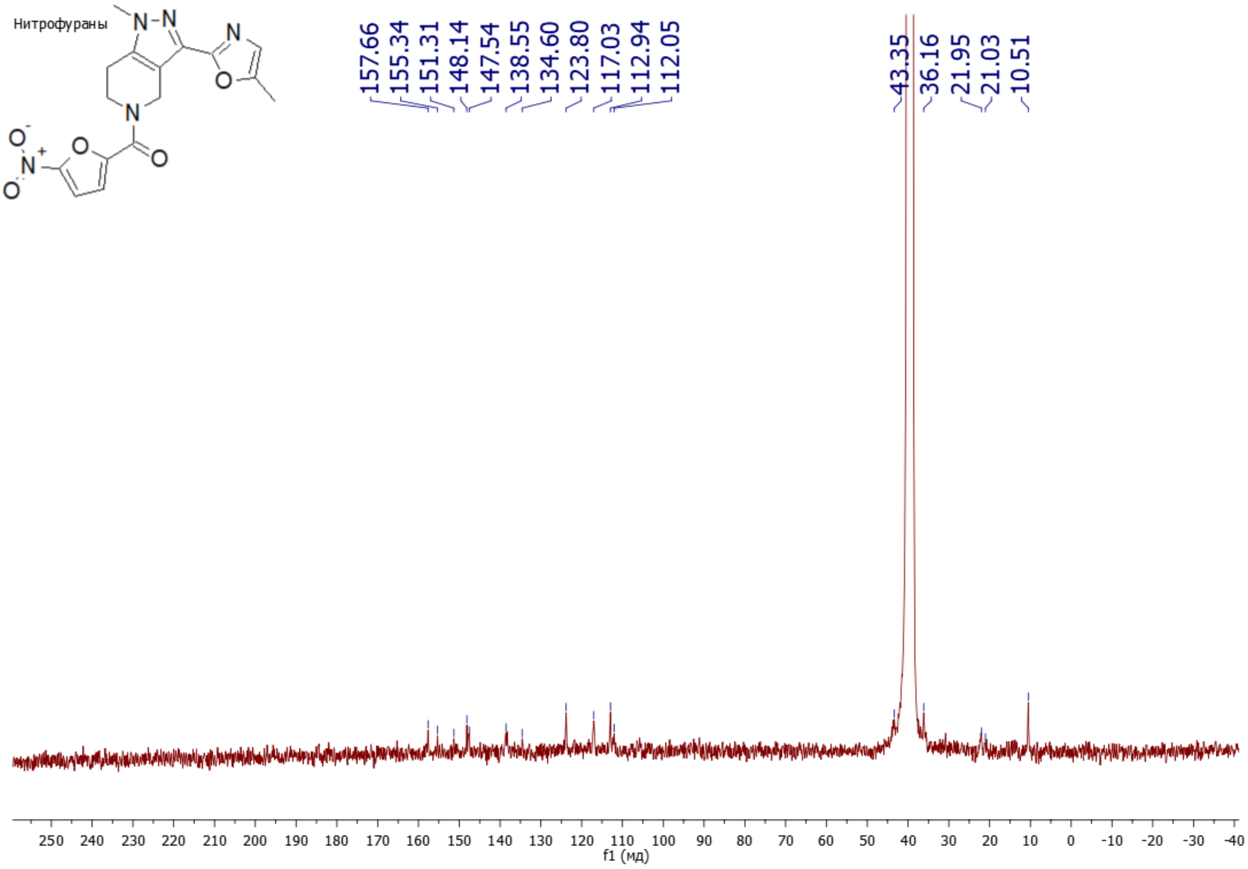

1-ethyl-3-(5-methyl-1,3-oxazol-2-yl)-5-(5-nitro-2-furoyl)-4,5,6,7-tetrahydro-1H-pyrazolo [4,3-c]pyridine, LK01511 (10b)

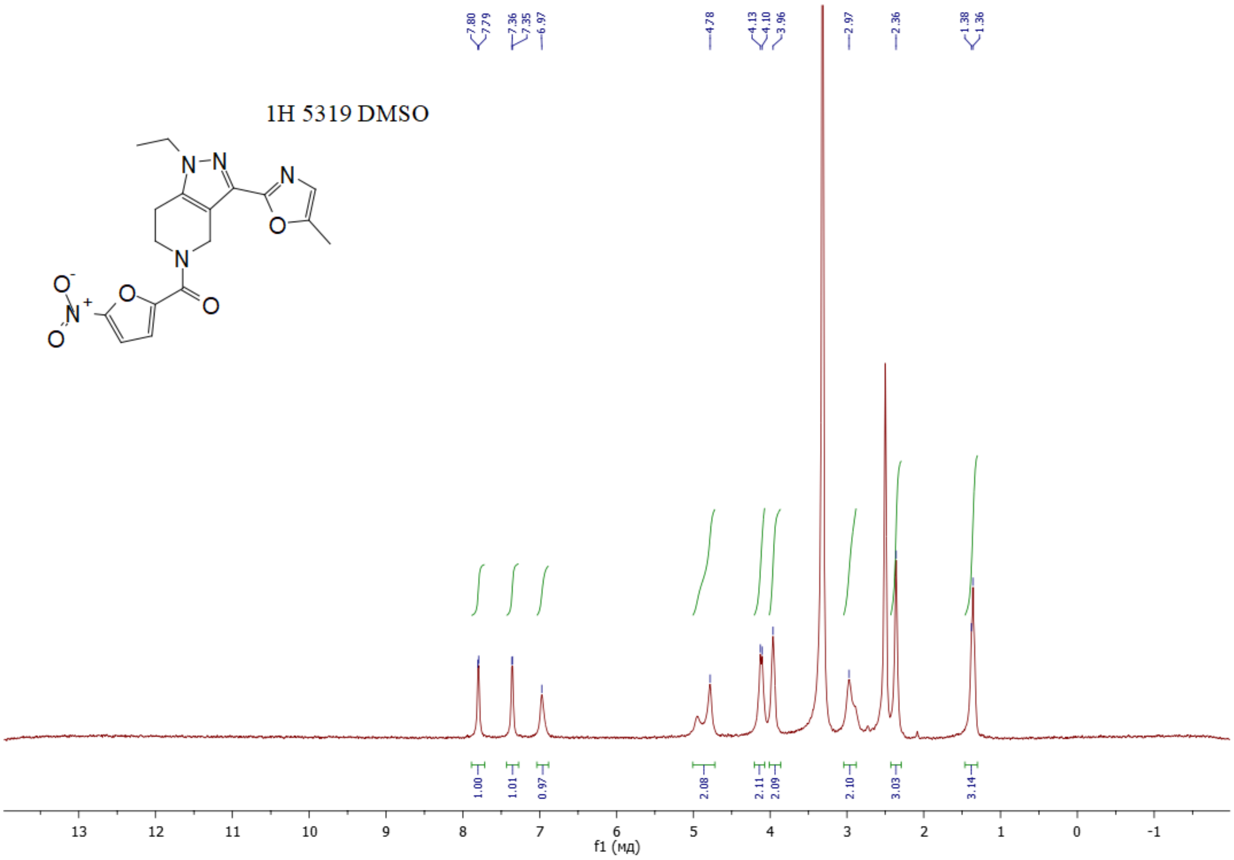

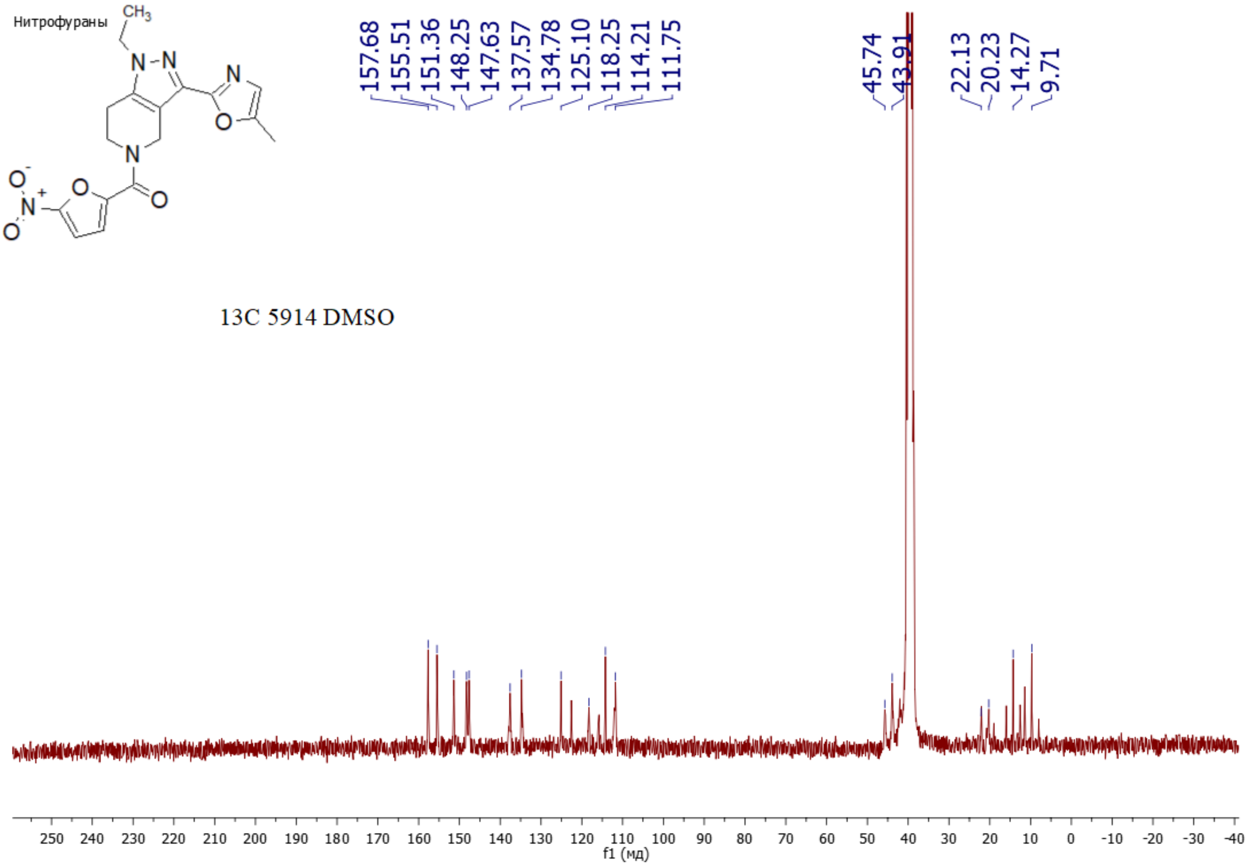

1-isopropyl-3-(5-methyl-1,3-oxazol-2-yl)-5-(5-nitro-2-furoyl)-4,5,6,7-tetrahydro-1H-pyrazolo [4,3-c]pyridine, LK01515 (10c)

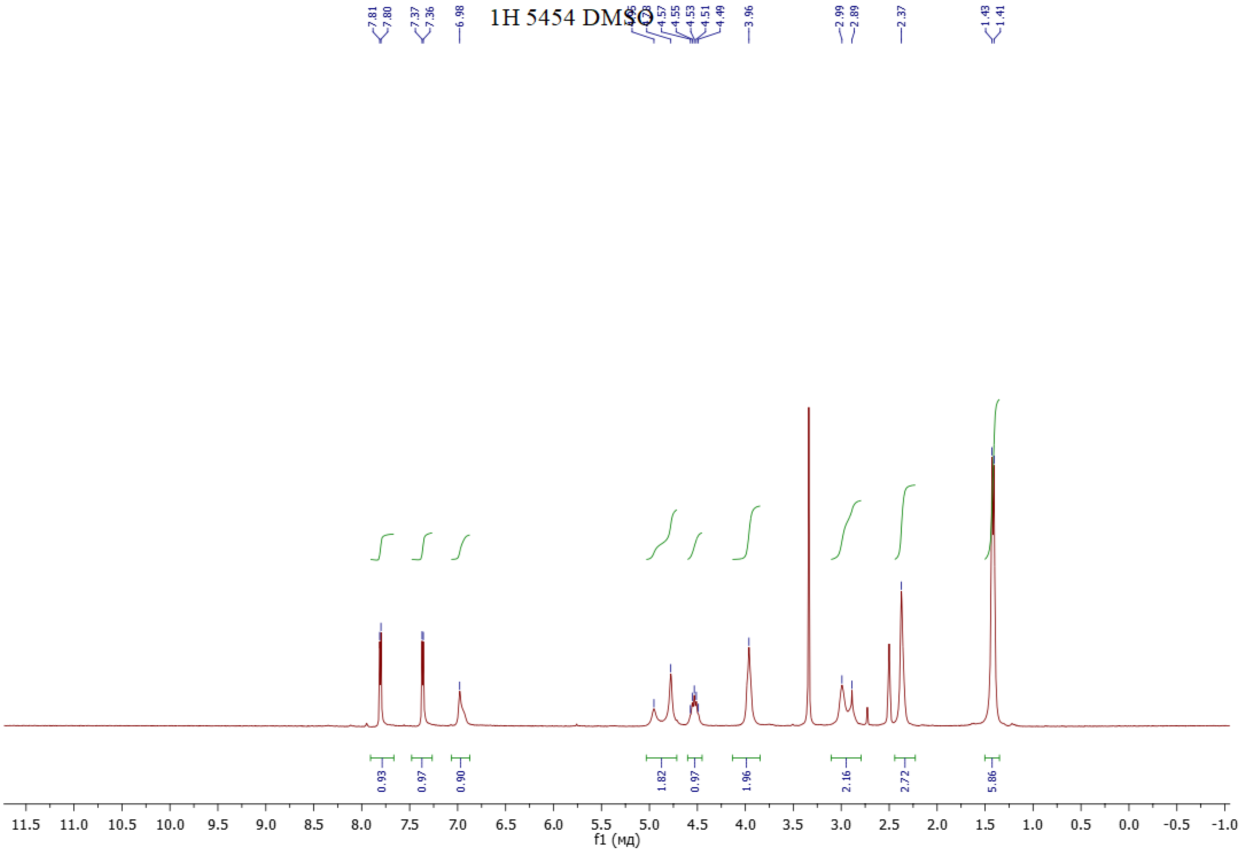

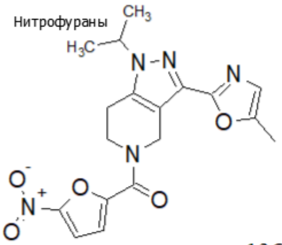

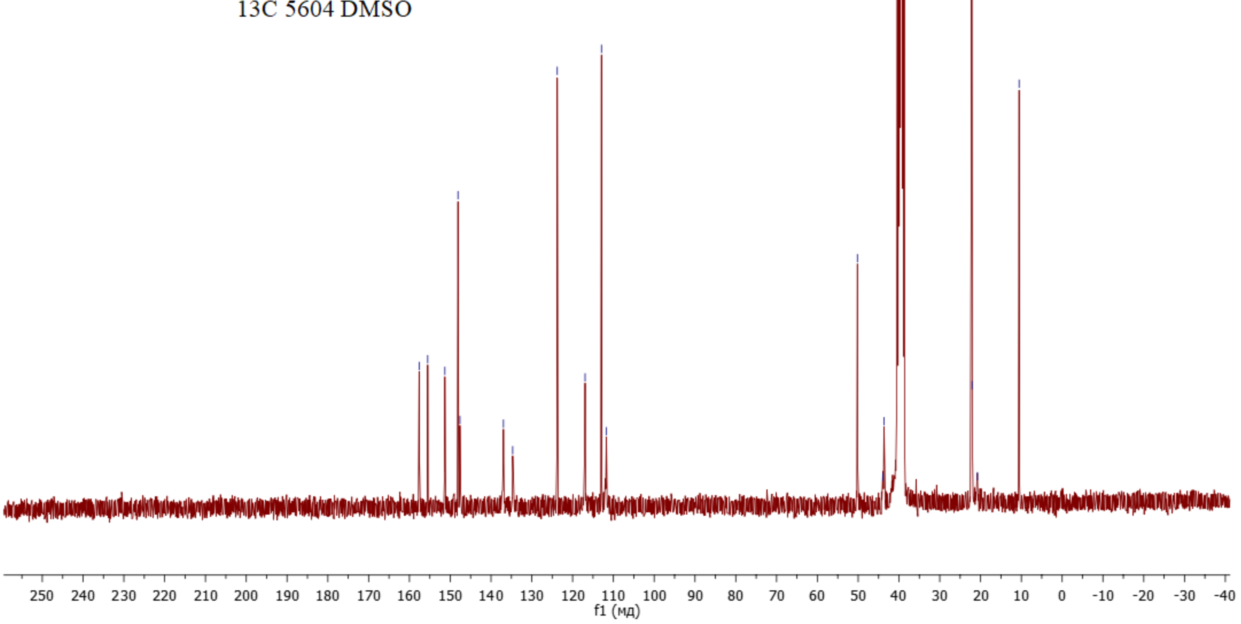

1-isobutyl-3-(5-methyl-1,3-oxazol-2-yl)-5-(5-nitro-2-furoyl)-4,5,6,7-tetrahydro-1H-pyrazolo [4,3-c]pyridine, LK01516 (10d)

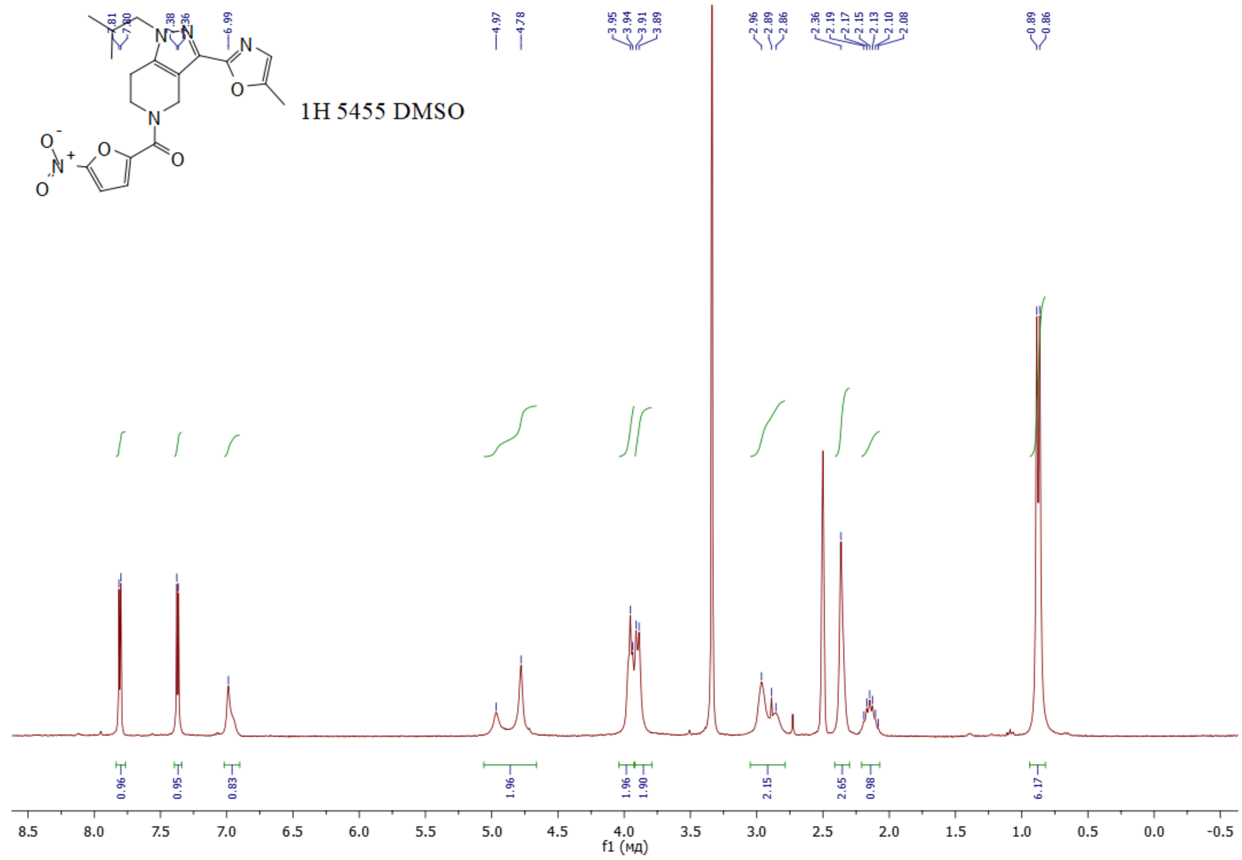

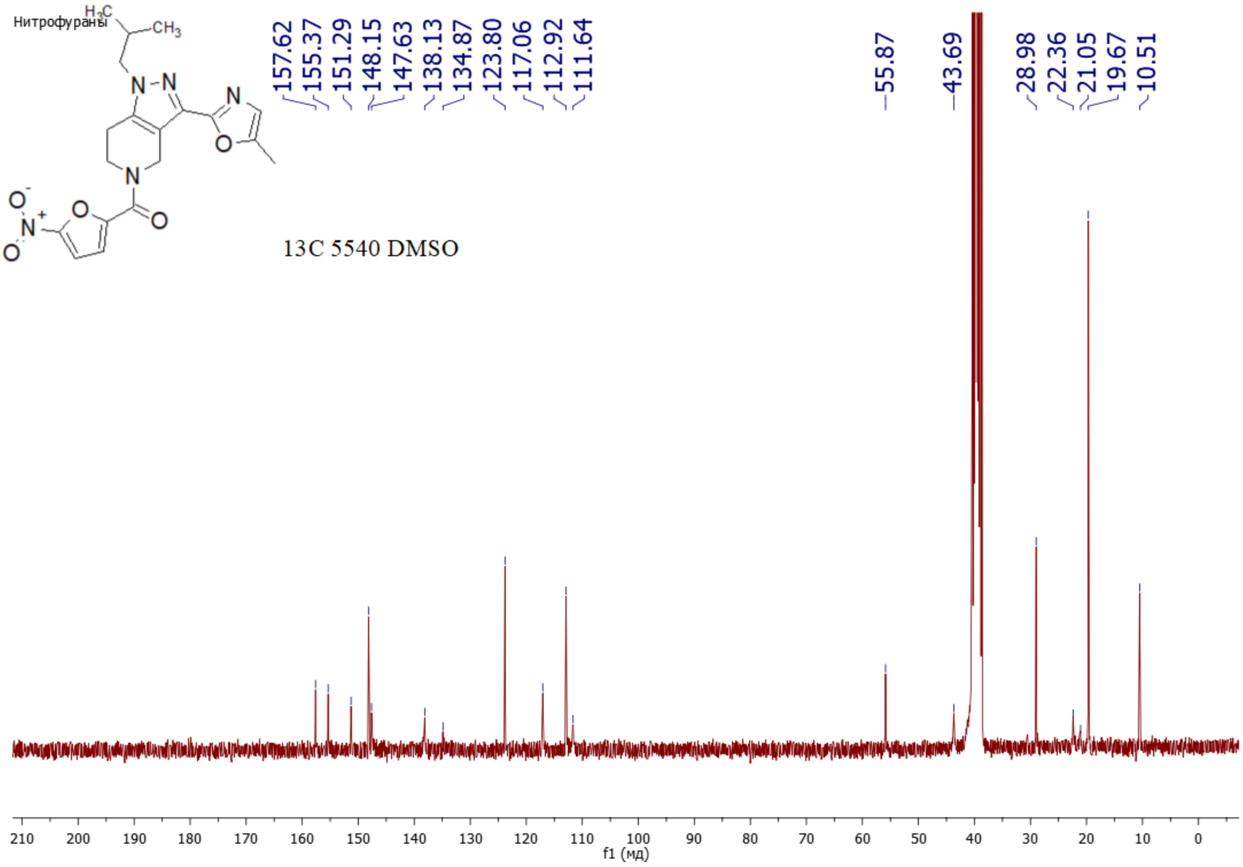

1-(cyclopropylmethyl)-5-(5-nitro-2-furoyl)-3-(1,3-oxazol-5-yl)-4,5,6,7-tetrahydro-1H-pyrazolo [4,3-c]pyridine, LK01512 (13e)

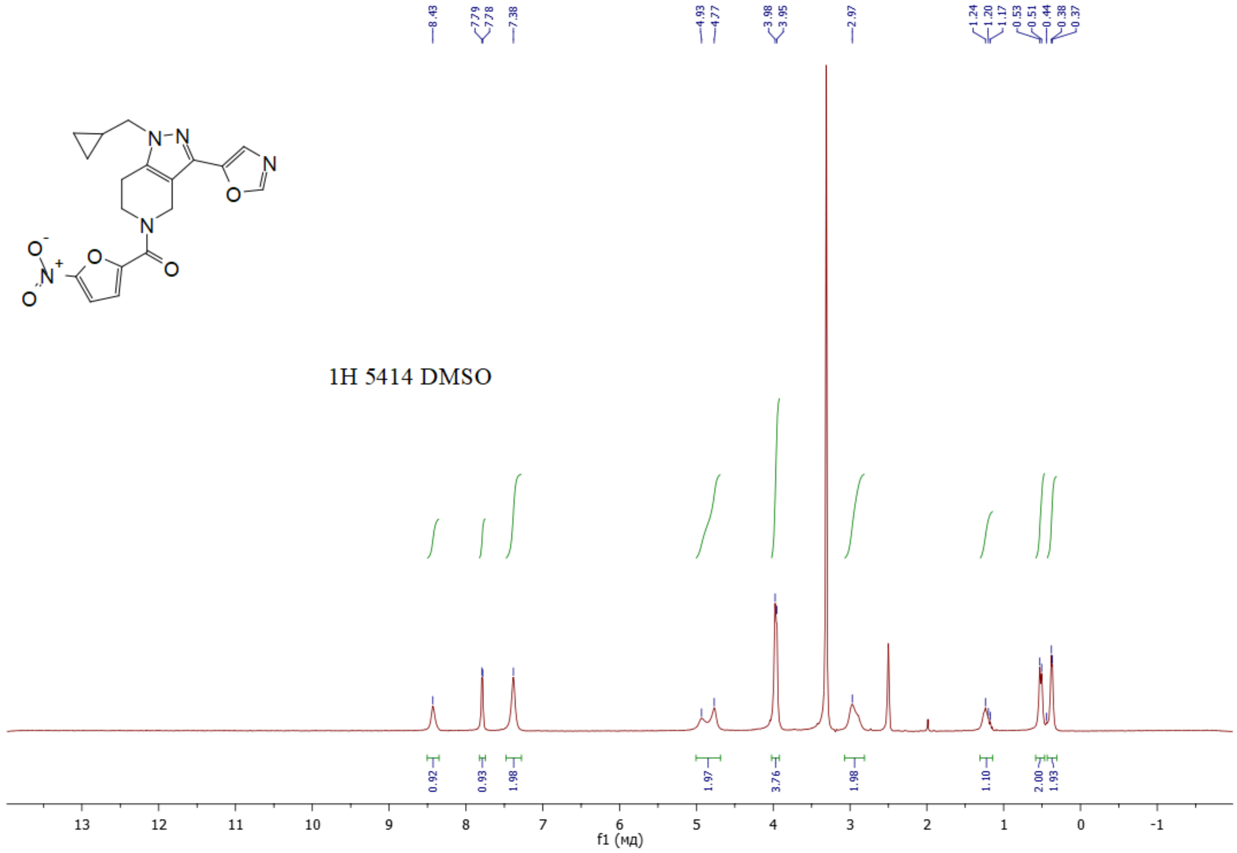

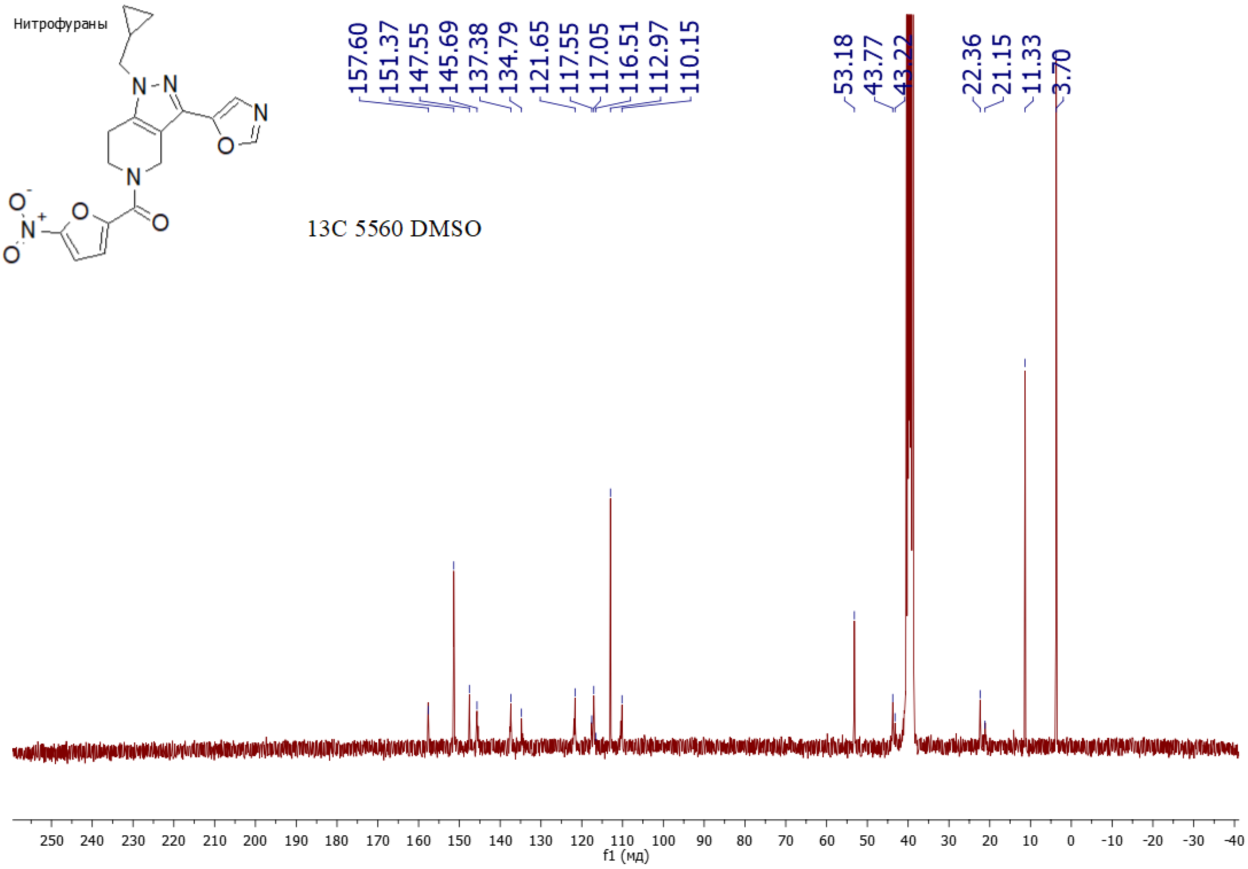

5-(5-nitro-2-furoyl)-3-(1,3-oxazol-5-yl)-1-propyl-4,5,6,7-tetrahydro-1H-pyrazolo[4,3-c]pyridine, LK01513 (13f)

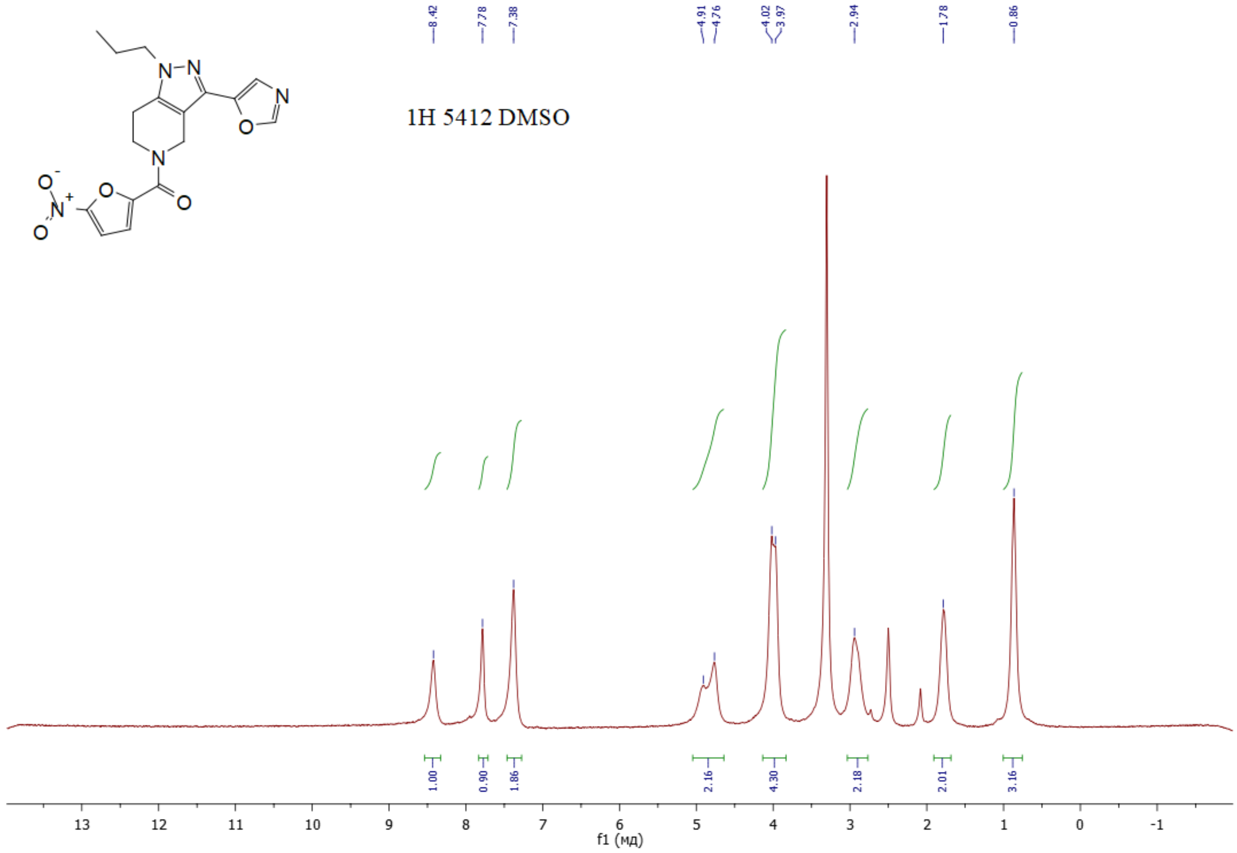

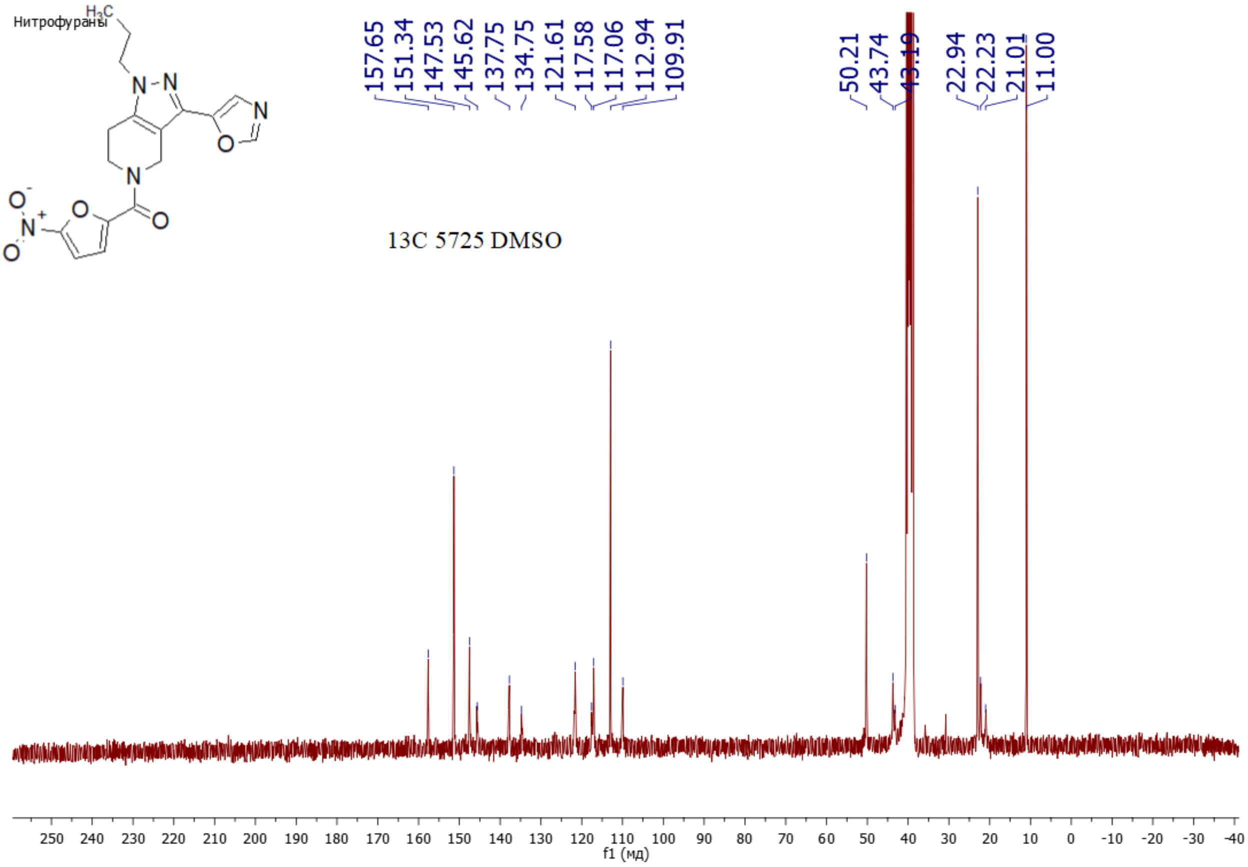

1-(2-methoxyethyl)-5-(5-nitro-2-furoyl)-3-(1,3-oxazol-5-yl)-4,5,6,7-tetrahydro-1H-pyrazolo [4,3-c]pyridine, LK01509 (13g)

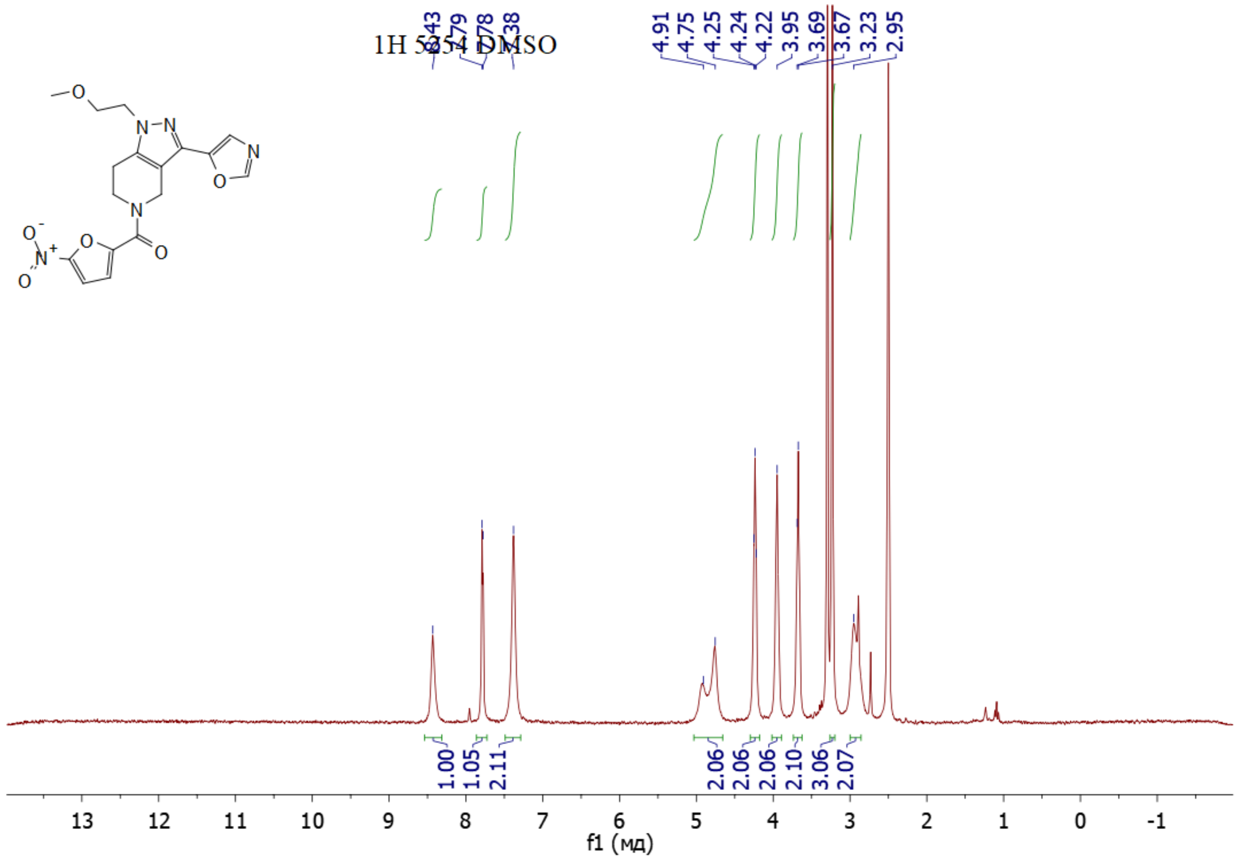

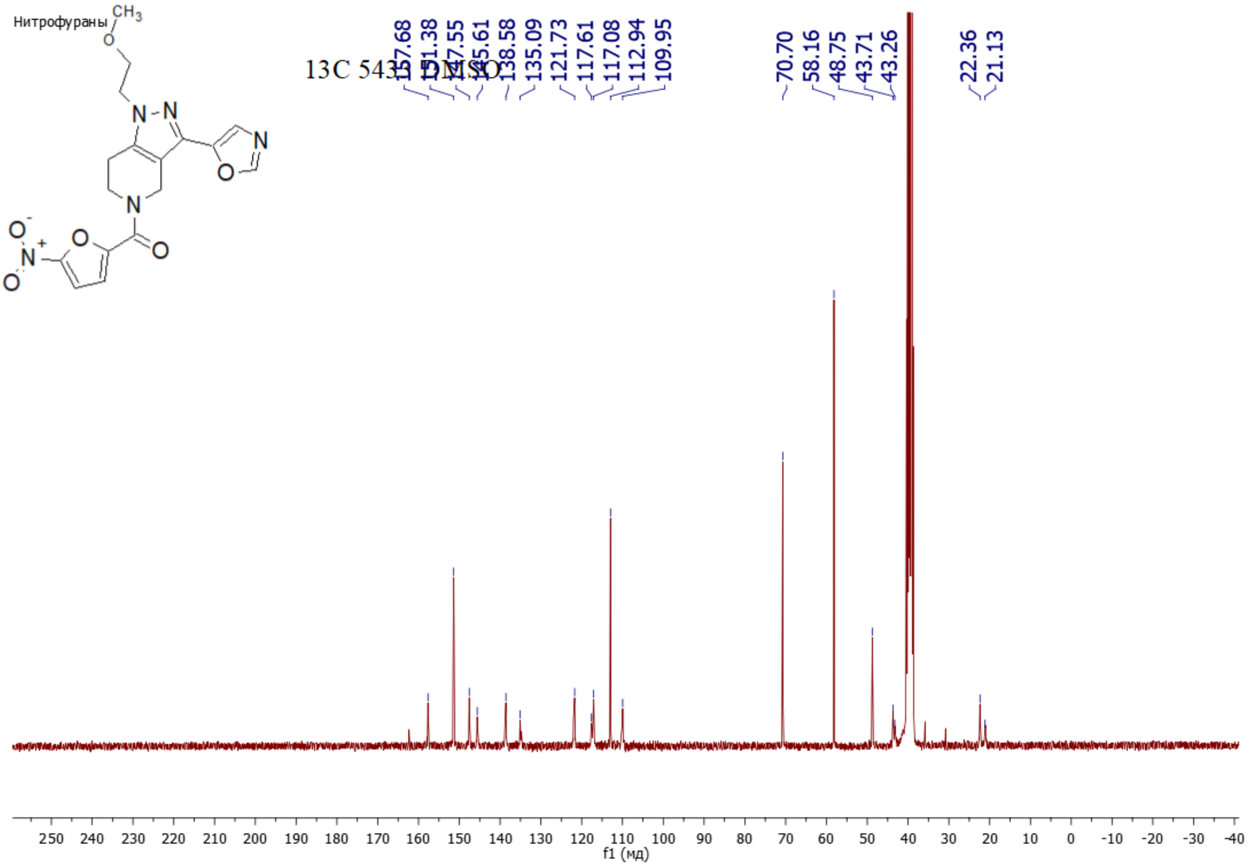

1-(3-methoxypropyl)-5-(5-nitro-2-furoyl)-3-(1,3-oxazol-5-yl)-4,5,6,7-tetrahydro-1H-pyrazolo [4,3-c]pyridine, LK01514 (13h)

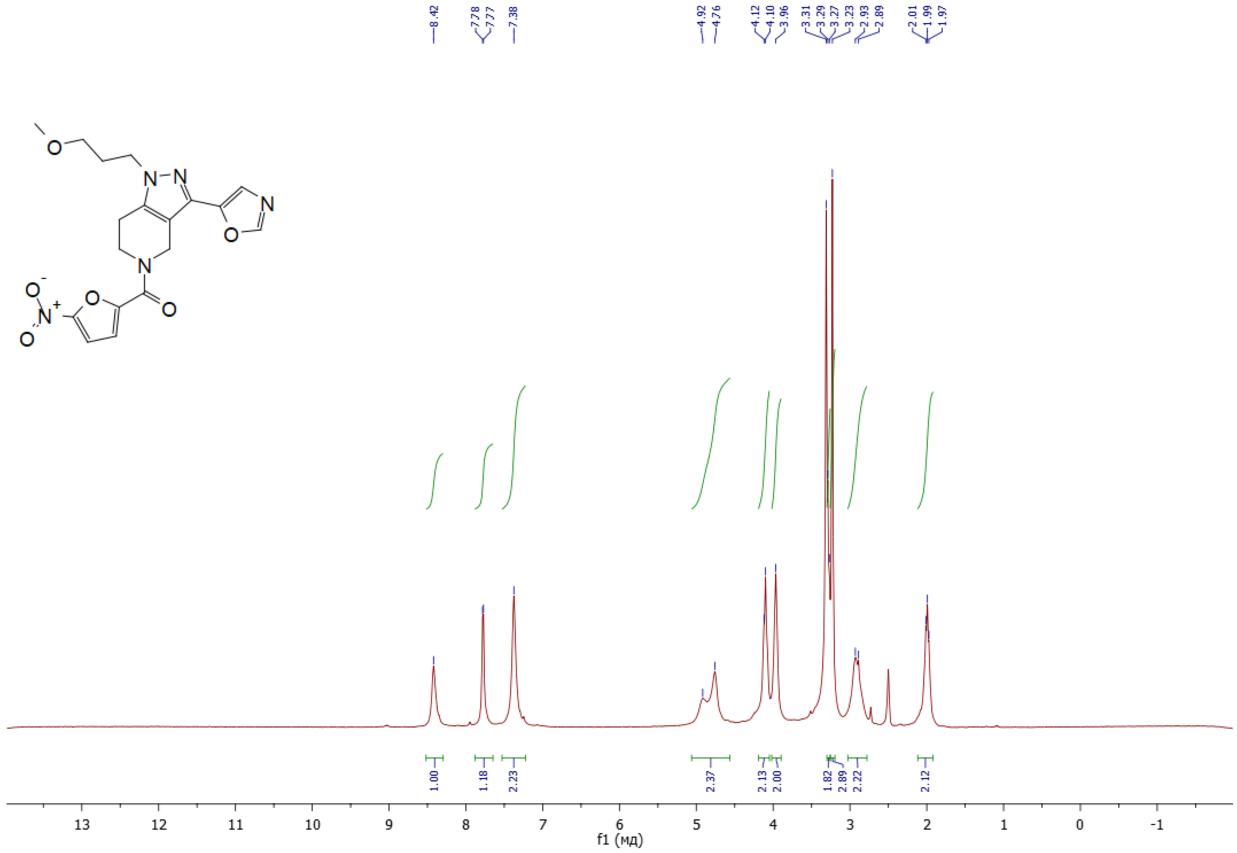

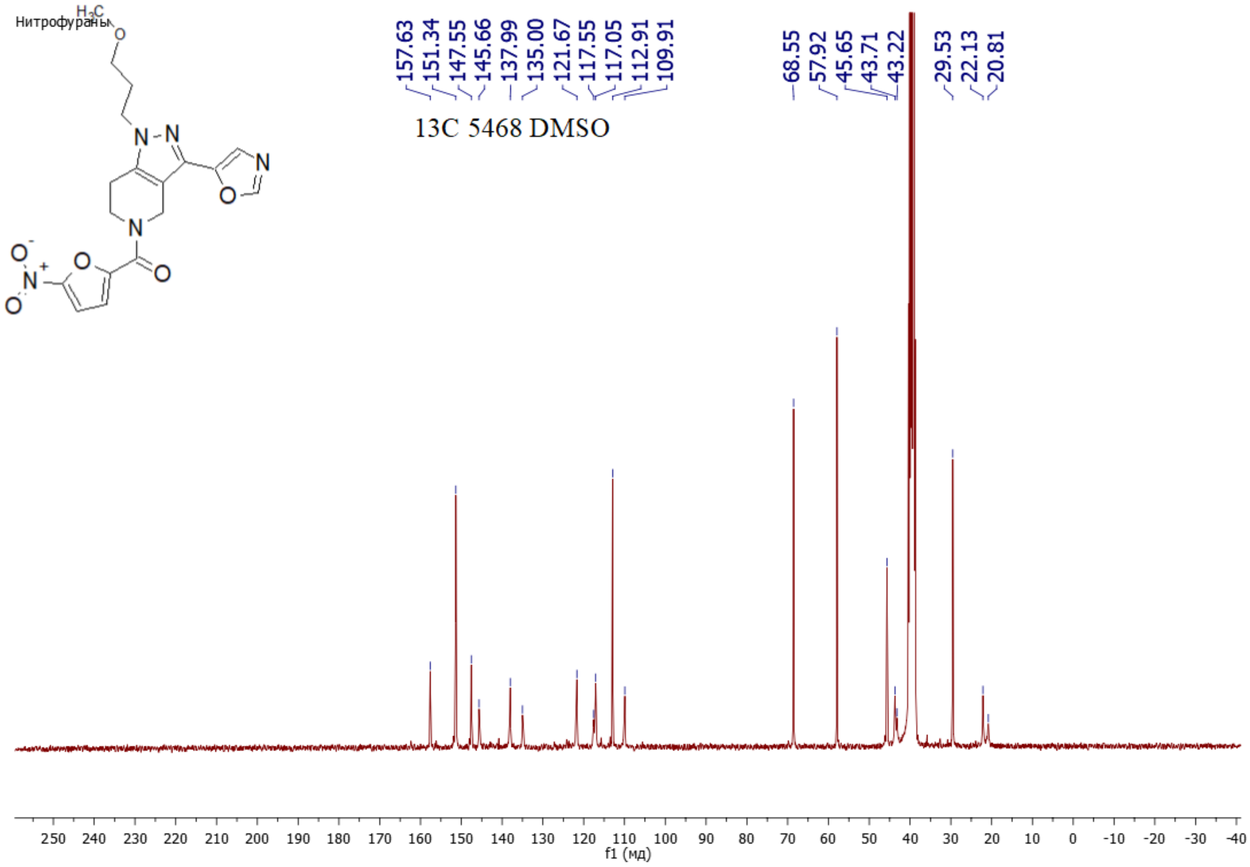

Supplement: Supplementary file 1 [file molecules-28-06491-s001.zip › molecules-2556239-supplementary.pdf]
